# Role of water in cyclooxygenase catalysis and design of anti-inflammatory agents targeting two sites of the enzyme

**DOI:** 10.1038/s41598-020-67655-6

**Published:** 2020-07-01

**Authors:** Manpreet Kaur, Baljit Kaur, Jagroop Kaur, Anudeep Kaur, Rajbir Bhatti, Palwinder Singh

**Affiliations:** 1 UGC Sponsored Centre for Advanced Studies, Department of Chemistry, Guru Nanak Dev University, Amritsar, 143005 India; 2 Department of Pharmaceutical Sciences, Guru Nanak Dev University, Amritsar, 143005 India

**Keywords:** Biocatalysis, Enzyme mechanisms, Structure-based drug design

## Abstract

While designing the anti-inflammatory agents targeting cyclooxygenase-2 (COX-2), we first identified a water loop around the heme playing critical role in the enzyme catalysis. The results of molecular dynamic studies supported by the strong hydrogen-bonding equilibria of the participating atoms, radical stabilization energies, the pK_a_ of the H-donor/acceptor sites and the cyclooxygenase activity of pertinent muCOX-2 ravelled the working of the water–peptide channel for coordinating the flow of H**·**/electron between the heme and Y385. Based on the working of H**·**/electron transfer channel between the 12.5 Å distant radical generation and the radical disposal sites, a series of molecules was designed and synthesized. Among this category of compounds, an appreciably potent anti-inflammatory agent exhibiting IC_50_ 0.06 μM against COX-2 and reversing the formalin induced analgesia and carageenan induced inflammation in mice by 90% was identified. Further it was revealed that, justifying its bidentate design, the compound targets water loop (heme bound site) and the arachidonic acid binding pockets of COX-2.

## Introduction

**Figure 1:**
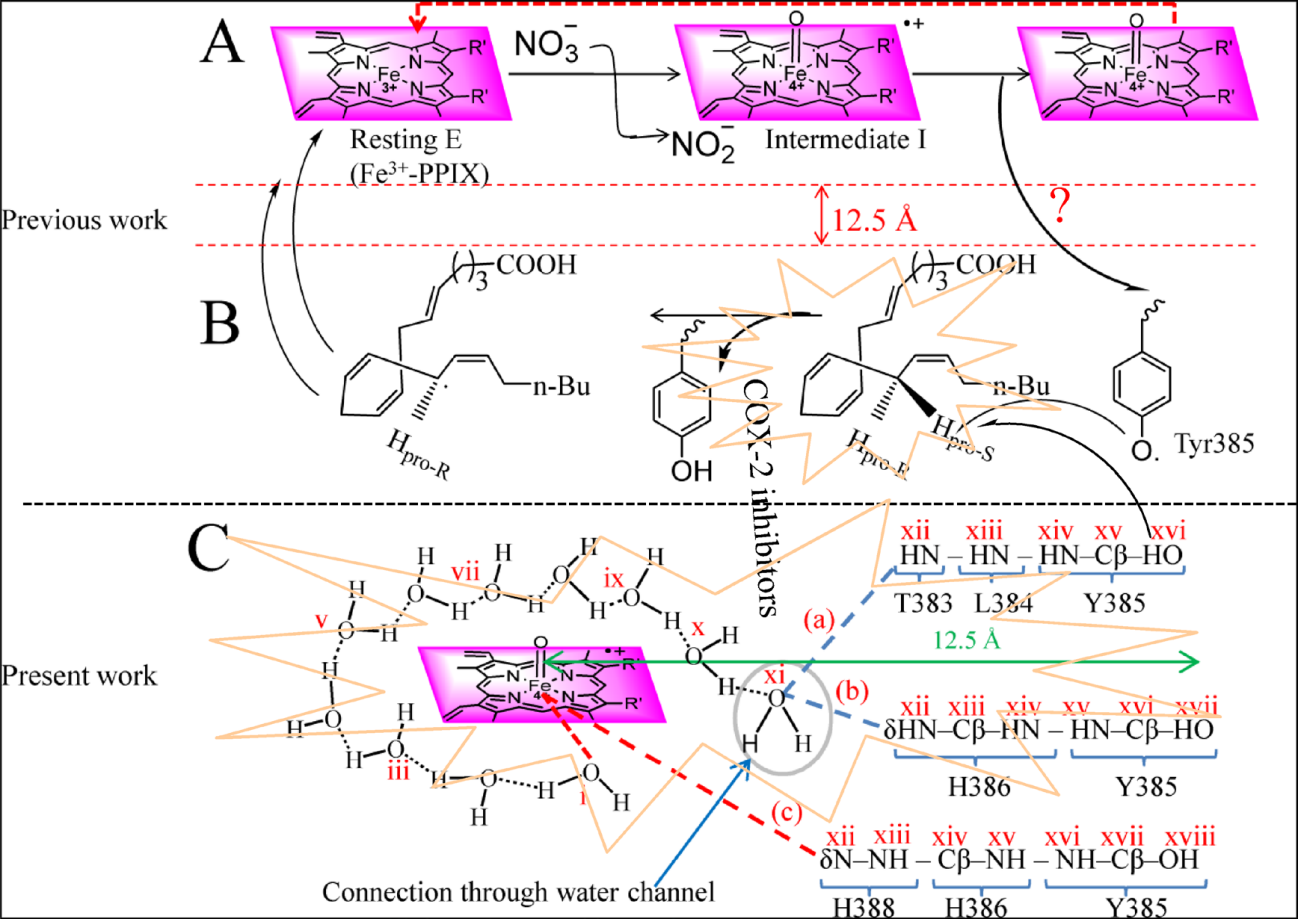
Cyclooxygenase catalysis: Reported working of COX-1/2 showing radical (**A**) at heme and its transfer (**B**) to Y385. (**C**) ‘a’, ‘b’: proposed electron transfer route (labelled i-xvi/xvii/xviii) through H-bonded water loop and the peptide; ‘c’: direct interaction of ferryl heme with the peptide. The stars represent the action of reported COX-2 inhibtors in (**B**) the arachidonic acid (AA) binding pocket and (**C**) the proposed plan for covering the heme and the AA binding pockets .

The cyclooxygenase-2 (COX-2) mediated metabolism of arachidonic acid (AA) synthesizing PGE_2_, PGD_2_ and PGF_2_ carry immense physiological significance due to the role of these metabolites in commencing inflammatory diseases including cardiac, cancer, arthritis, diabetes and neurological disorders^1–6^. Subsequent to the identification of the two isozymes of cyclooxygenase-COX-1 and COX-2, assigning house-keeping functions to the constitutive COX-1 and the role of inducible COX-2 in instigating inflammation, the arachidonic acid metabolism is profoundly explored in the last three decades^7–12^ leading to the switching of anti-inflammatory drugs from non-selective to selective non-steroidal COX-2 inhibitors^13–17^. Mechanistically, the COX-2 mediated metabolism of AA consisting jointly the peroxidase activity for the generation of radical at the prosthetic group heme and its transfer to the tyrosyl residue positioned in the vicinity of AA has been extensively investigated^18–25^. However, a major concern of the reported mechanism is the connecting path between the radical generation centre and the oxygenation site because the two events occur in separate pockets of COX-1/2 which, as per the crystal coordinates of COX-2—AA—heme complex, are 12.5 Å far from each other (Fig. 1A,B)^26^. Nonetheless, the tunnelling effect is taken into account for the distant interactions of the reactive sites in proteins through the intervening organic medium^27–32^ but all the radical carrier atoms for such a process in cyclooxygenase are not recognized. Hence, in the absence of clear picture of radical transfer channel, the COX-2 inhibitors reported so far were developed keeping in view their interaction only in the AA binding site of the enzyme. Bearing in mind the radical mechanism of AA metabolism^23^, here, we first explored the channel for the radical transfer from its generation site heme to the disposal site Y385 (Fig. 1C) and then tried to disturb this channel (in addition to the arachidonic acid binding site) for developing the anti-inflammatory agents.

## Results and discussion

### Molecular dynamics of COX-2—AA–heme complex for tracing the electron transfer channel

**Figure 2:**
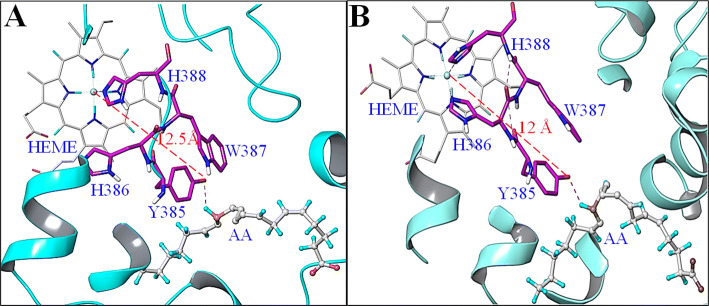
(**A**) Crystal coordinates of COX-2–AA as available in the protein data bank. The distance between the Fe and oxygen of Y385 is 12.5 Å. (**B**) A pose of MD trajectory of COX-2–AA complex showing no much change in relative placement of active site components.

Besides providing the required flexibility for the conformational changes in the protein, water plays specific role in enzyme catalysis^33–36^. While the participation of one or two water molecules in the catalytic domain of several enzymes is reported^37–44^, we checked if the randomly associated water molecules undergo certain reorganization to mediate H**·**/e transfer during the catalytic phase of the enzyme. Since the presence of heme is essentially required in the enzyme activity, the available wt COX-2—AA—heme crystal coordinates (pdb ID 3HS5)^26^ (Fig. 2A), best representing the physiological state of the enzyme (including heme as well as the substrate), were analyzed. The two distinct pockets of the enzyme, one carrying heme as the radical source and the second occupying Y385 and AA as the radical disposal end are 12.5 Å from each other leaving no possibility of their direct interaction. However, the limitation of the static crystal structure, not truly representing the reactive coordinates of the enzyme, incited us to investigate the dynamics of COX-2—AA—heme complex if somehow the conformational changes bring ferryl heme and Y385 close to each other. In this context, molecular dynamics (MD) of COX-2—AA—heme complex in aqueous medium over 50 ns was performed, capturing and analyzing 5,000 frames during the whole process (Figure S1–S4). While significant conformational changes in the structure of COX-2 as well as AA were observed no much perturbation was noticed in the active site pocket of the enzyme hosting AA. We did not find a single event where ferryl heme to Y385 distance was < 12 Å (Fig. 2B) and hence, the possibility of direct interaction of the heme and Y385 for the transfer of H^**·**^ may not subsist.

**Figure 3:**
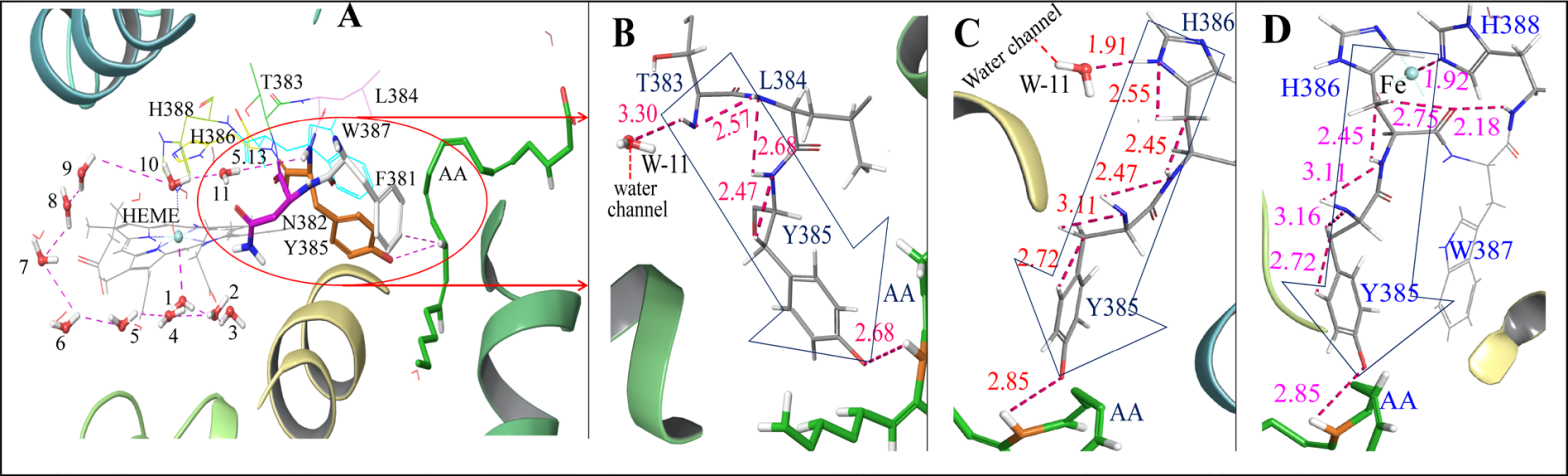
MD studies: (**A**) COX-2–AA–heme complex showing loop of water molecules (1–11) connecting Fe to the peptide through (**B**) T383 (**C**) H386, corresponding to channel ‘a’ and ‘b’, respectively in Fig. 1C for the radical transfer from heme to Y385; (**D**) Connection of Fe with Y385 through H388-H386 corresponding to channel ‘c’ in Fig. 1C.

Further analysis of the MD data exposed a very ordered channel of water molecules (numbered 1–11, numbering in the crystal coordinates was different, Figure S5) in the active site pocket of the enzyme making a loop around the heme (Fig. 3A). Water molecule-1 (W-1) interacted with the ferryl heme at a distance 1.70 Å whereas the last molecule of the loop (W-11) was located in the H-bonding range with N**H** of T383 and δN**H** of H386 (Fig. 3B,C). While this channel was not seen in the static crystal coordinates of the COX-2—AA—heme complex, the molecular dynamics recorded its existence over a period of 100 ps. The H-bond distances in the range of 1.72–3.30 Å (Figure S6) between the water molecules of the loop and further between W-11 and N**H** of T383 and δN**H** of H386 confirmed their strong interactions making an intact path for the H**·**/e transfer. The only other interactive site near W-11 was N**H** of Y385 at 5.13 Å leaving very little chances of H-bonding. None of the other 10 water molecules of the loop made contact with the peptide chain. The NH and δNH of T383 and H386, respectively were further connected to Y385 through strong intra-peptide H-bonding (Fig. 3B,C). Therefore, besides the direct interaction between the heme and the protein through H388 (Fig. 3D), the connections through channels ‘a’ and ‘b’ were contemplated.

### Thermodynamics and kinetics of electron transfer over the three channels

**Figure 4:**
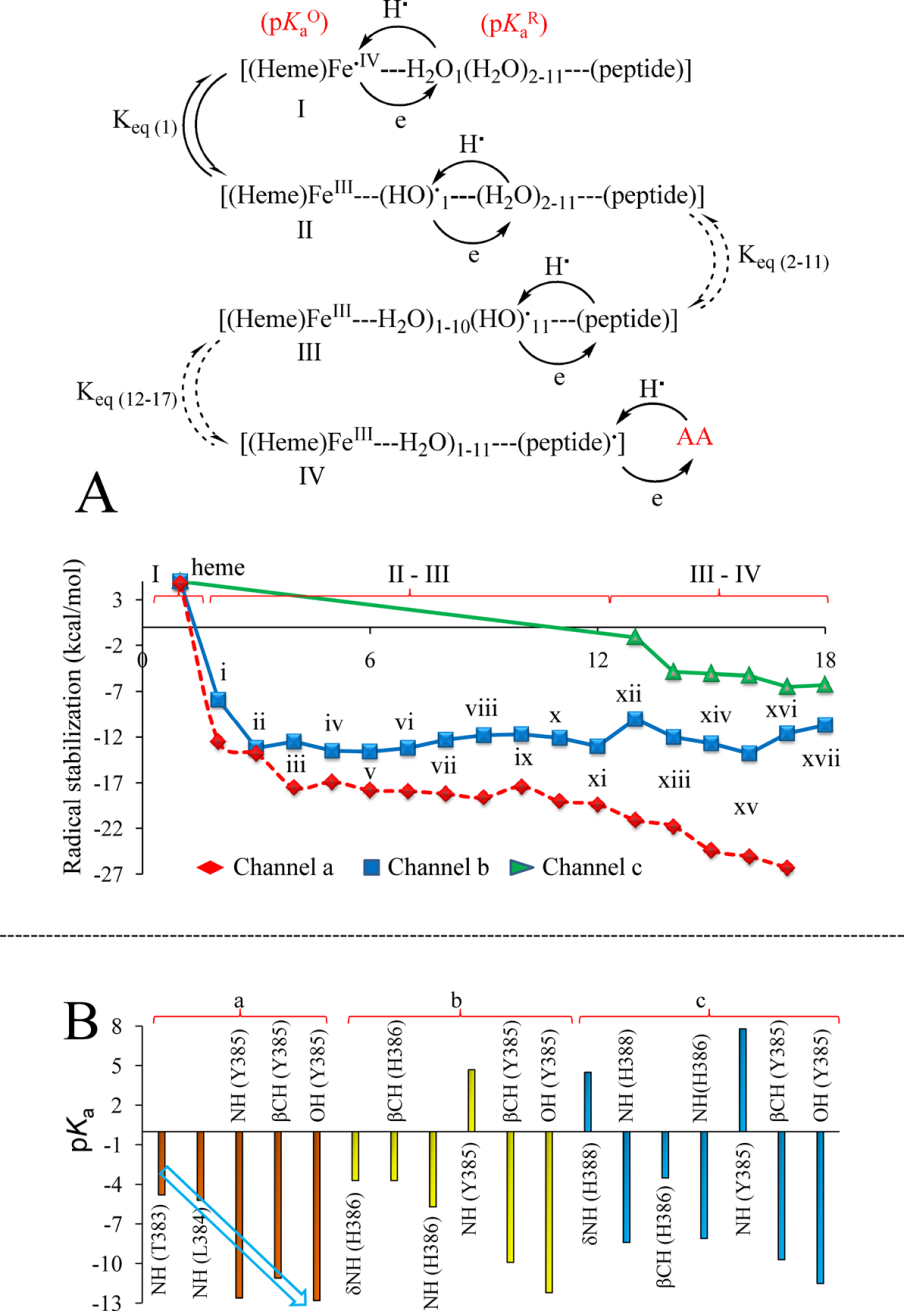
Simulations over the COX-2–AA–heme complex: (**A**) Equilibria between the consecutive steps of the H**·**/e transfer process and the corresponding radical stabilization of three channels (i–xviii, Fig. 1C) (**B**) p*K*_a_ of the peptide participating groups of the three channels. All values with SD ± 0.5 units.

Although the H-bond distances over the three channels were comparable and provide compelling evidences for the proton coupled electron transfer (PCET)—an ubiquitous electron transfer mode in biology^45–47^, radical stabilization energy (∆G) at each of the participating atoms (i–xviii, Fig. 1C) w.r.t. heme along with the equilibrium constant (*K*_eq_) for every consecutive step of the three channels were calculated as a way of predicting their relative operational feasibility (Figure S7–S9). Starting with the initial thermal equilibrated system and performing at 298 K with entropy factor constant, the energy of the system was considerably decreased for both the channels ‘a’ and ‘b’ when shifting the radical from heme to W-1 (I to II, Fig. 4, Table S1) by the transfer of H**·** from W-1 to heme assuming concerted mechanism for electron and proton transfer. The energy change was also apparent from 3 × fast predicted rate at step II (Table S2i). With the substantial decrease in energy of radical over the water loops of channel ‘a’ and ‘b’ (II to III, Fig. 4), gradual stabilization occurred when the radical traversed the peptide part of channel ‘a’ (III–IV, Fig. 4, Table S1) making an overall energy decrease of 31 kcal/mol in comparison to 15 kcal/mol on channel ‘b’. Characteristically, the radical carrying atoms on the peptide chain were all Ns’ for channel ‘a’ except one βC (benzylic centre xvi) whereas for channels ‘b’ and ‘c’, high energy carbon centred radicals at position xiii, xvi and xiv, xvii, respectively were developed. The series of equilibrium constant expressions also preferred the movement of H**·** through channel ‘a’ though the difference from the other channels was not significant (Table S2i). When comparing with channel ‘c’ (Table S1), it seems that the delocalization of the radical over the water loop assisted in decreasing the energy of the system and further in association with the H-bonded network on the peptide, channel ‘a’ was more suitable for accomplishing the electron transfer between Y385 and heme. Once O**·**-Y385 is generated, being placed just 2.85 Å off 13-pro-(*S*)-H of AA (Fig. 3B–D), it can directly abstract the H**·**.

### p*K*_a_ of the participating groups

Since the acid dissociation constant plays decisive role in the proton transfer within a chain, the p*K*_a_ of the participating groups were determined in the protonated (p*K*_a_^R^) and the deprotonated (p*K*_a_^O^) forms. For channel ‘a’, starting with W-1, the p*K*_a_ was systematically decreased from − 22.4 at W-1 to − 3.4 at W-11 whereas it varied between the respective ends from − 20.3 to − 1.1 for channel ‘b’ (Table S2i). Availability of proton with the donor groups and satisfying the condition p*K*_a(acc)_ > p*K*_a(don)_; unlike channel ‘b’ and ‘c’, the p*K*_a_ of the groups xii–xvi of channel ‘a’ was found desirably decreasing when moving towards Y385 (Fig. 4B, Table S2ii). Moreover, the possibility of taking extra proton from the medium when the electron is transferred was ruled out on the basis of p*K*_a_^R^ < pH (physiological pH) that indicated the preference for the intra-protein H transfer. Therefore, desired trend of p*K*_a_ over channel ‘a’ along with the RSEs’ and positive equilibrium constants jointly coordinated the working of the strongly coupled Bronsted acid–base composites and provided desired driving force for the movement of the H**·**/e from one end of the channel to the other end.

### Cyclooxygenase activity of the mu COX-2

**Figure 5:**
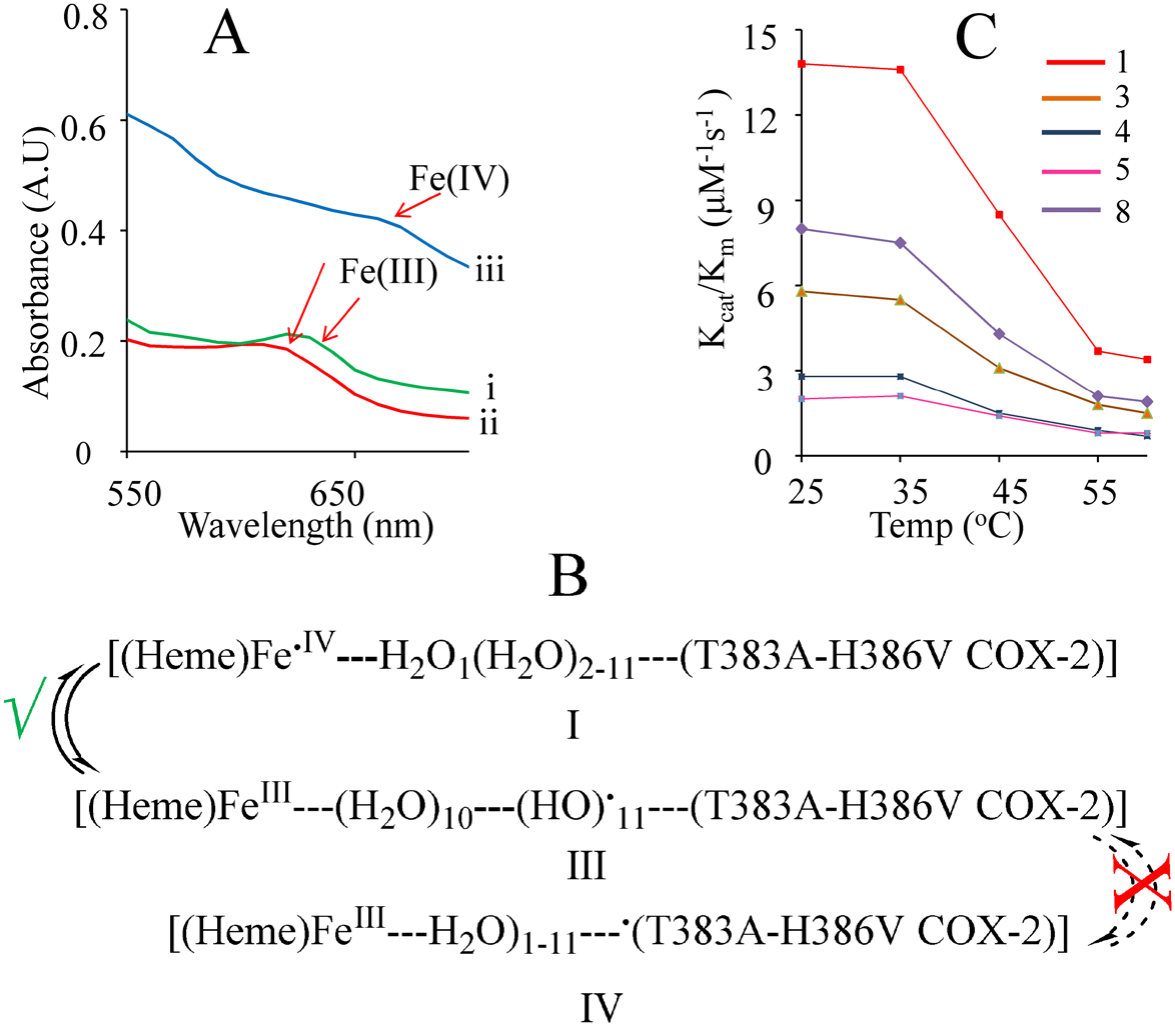
Experimental studies with wt- and mu-COX-2: (**A**) UV–vis spectra of T383A-H386V muCOX-2 reaction: (i) no AA, (iii) AA, (ii) after 5 min of AA addition. (**B**) schematic representation of blockage of H**·**/e transfer in T383A- H386V COX-2. (**C**) Change of enzymatic activity with temperature. 1–8 correspond to entries in Table 1. All values were average of 3 expts, SD ± 0.5–2.0 µM^-1^ s^-1^.

Supporting the role of water loop in the working of the enzyme, restoration of 610 nm band was observed after its shifting to 650 nm in the case of T383A—H386V muCOX-2 reaction (Fig. 5A, S11) though this enzyme did not perform AA metabolism (Table 1). Apparently, the radical generated at the heme (Fe(IV) (650 nm)) during the initial step of the enzyme catalysis might be converted to Fe(III) (610 nm) due to the H**·** transfer from W-1 to heme (stage I to II–III, Fig. 5B). However, the radical from the water loop did not pass to the peptide (no conversion of stage III to IV, Fig. 5B) and no AA metabolism was pursued. Further, the graded change in the activity of N382A, T383A, F381A, L384F, Y385F, H386V and H388V muCOX-2 w.r.t. wt COX-2 (Table 1) indicated the preferred movement of H**·**/e through T383-L384-Y385 chain when 60–65% decrease in the AA metabolism was observed for T383A COX-2 and L384F COX-2 and absolutely no activity of Y385F muCOX-2. For N382A and H386V muCOX-2, 35% decrease of AA metabolism was recorded and only 8–7% decrease was shown by H388V and F381A muCOX-2. The large change in the activity of H386V COX-2 in comparison to that of H388V COX-2 ruled out the involvement of channel ‘c’ in the electron transfer process. The decrease in the activity of N382A COX-2 complying with the conformational changes-altering the position of T383 and disturbing the electron transfer channel (Figure S12) supported the results of Kulmacz group^22,48^ for the role of N382 and T383 in the COX-2 catalysis. Determined as a way to detect the perturbations in the protein-site interactions with AA, the observed variation in *K*_*m*_ was consistent with the COX-2 activity of the recombinant proteins and indicative of the extent of structural contacts between the amino acid and AA (Table 1, Figure S14–S23). Further, arguing the role of water loop in the enzyme activity and expecting its disturbance at higher temperature, a large change in *K*_cat_/*K*_m_ of *wt* as well as the recombinant COX-2 for AA was observed (Fig. 5C, Table S3) though the enzyme activity in general is affected by the temperature. Although not specifically indicating the role of water loop but lyophilized COX-2 in acetonitrile did not work besides the reports on enzyme activity in non-aqueous solvents^37^. Further studies on temperature dependency of *K*_cat_/*K*_m_ may disclose its other implications in AA metabolism^49^.

**Table 1:** Catalytic activity of COX-2 for AA metabolism in terms of PGE_2_ formation (ng/mL) and kinetic constants. Average of 3 expts, SD ± 0.5–2.0.

**Entry**	**COX-2**	**Activity % of wt COX-2**	***K***_***m***_** (μM)**	***K***_**cat**_** (s**^**-1**^**)**	***K***_**cat**_**/K**_**m**_** (μM**^**-1**^** s**^**-1**^**)**
1	wt	100	4	55.2	13.8
2	F381A	93	8	45	5.6
3	N382A	67	6	35	5.8
4	T383A	40	10	28	2.8
5	L384F	36	12	25	2.0
6	Y385F	0	5	5.1	1.0
7	H386V	65	8	35	4.3
8	H388V	92	5	40	8
9	T383A, H386V	2	13	26	2
10	H388V, H386V	68	11	25	2.2

Therefore, the combined analysis of the results of molecular dynamics (MD) and the other contributing factors suggest that out of the three connections between the ferryl heme and Y385 (Fig. 1C: a, b, c), route ‘a’ seems the most likely channel for proton coupled electron transfer. Moreover, this information about the enzyme mechanism helped in the design of anti-inflammatory agents working through targeting the water channel as well as the AA binding site.

### Rational design of new COX-2 inhibitors

Analysis of COX-2 crystal coordinates clearly showed that the catalytic activity of the enzyme is initiated by the generation of radical at heme followed by its transfer to Y385 at a distance 12.5 Å with final disposal at AA 2.85 Å further to Y385 (Figure S24). Keeping in view the role of electron transfer channels in the catalytic mode of the enzyme, it was envisaged that in addition to the targeting of AA binding site, the radical generating allosteric site at ~ 14 Å and the radical transfer channel could also be the potential targets of anti-inflammatory drugs. While validating the model, the molecules consisting of hydrophobic and hydrophilic moieties at the two ends of the alkyl linker of varied length were designed assuming the placement of respective end in the AA binding hydrophobic site (Site 1) and the heme binding hydrophilic site (Site 2) of the enzyme. Empanelling the flexible alkyl chains and a rigid indole moiety, the hydrophilic-hydrophobic moieties such as acridine, indole and pyrimidine/ oxindole and dimethoxyphenol were introduced at the two terminals providing the requisite compounds **1**–**10** (Fig. 9) which were screened against COX-2 through enzyme immnunoassays and for the reversal of inflammation against the animal models.

**Figure 6:**
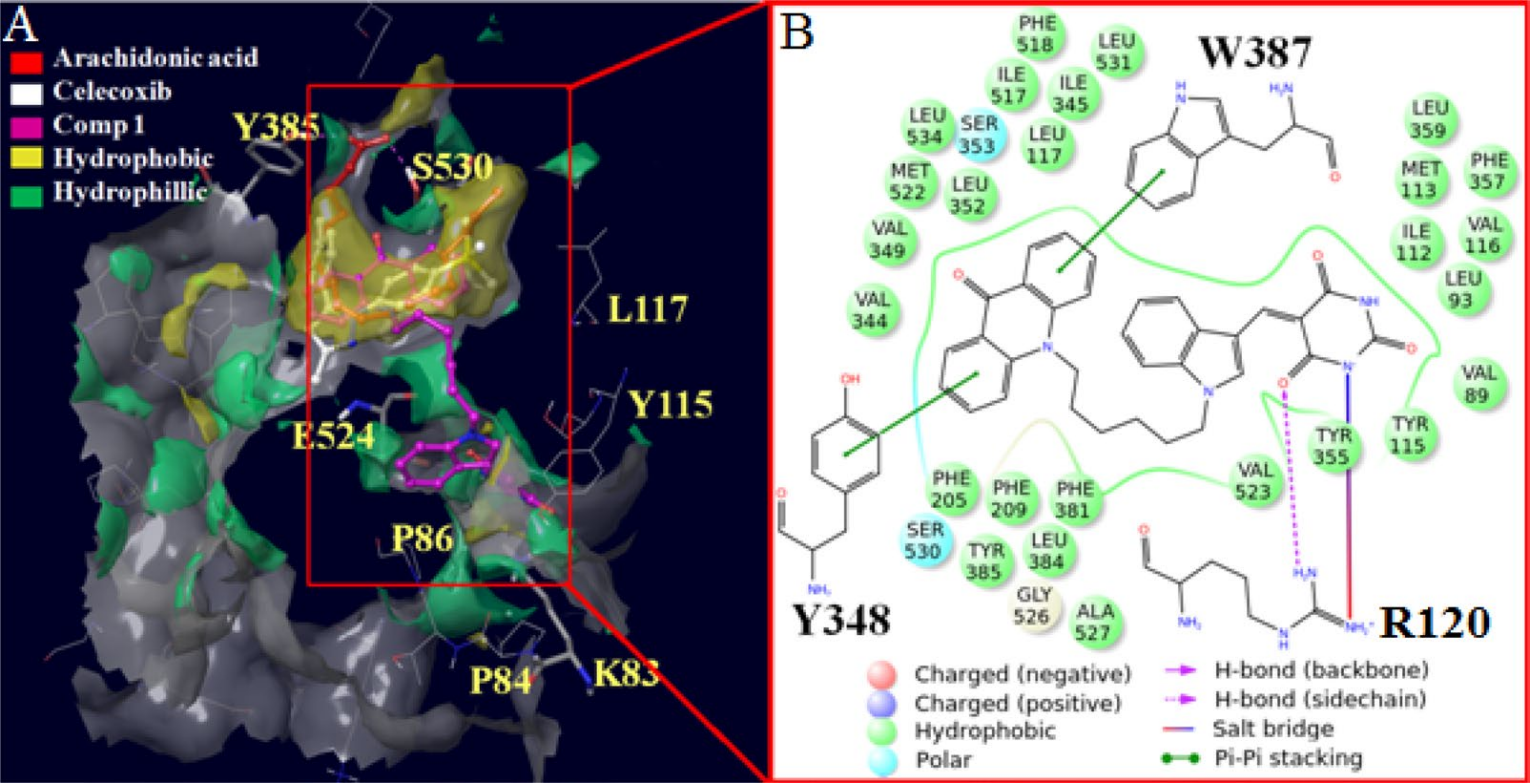
(**A**) Solid surface top view of the arachidonic acid binding pocket (site 1) and allosteric site (site 2) of COX-2 in association with **1** (pink). For site 1, the yellow part represents hydrophobic region whereas green is the hydrophilic region. For simplicity, the hydrophobic and hydrophilic regions of site 2 are not differentiated. (**B**) 2D view of red boxed part of the binding site showing interaction of **1** with amino acids.

The results of molecular docking of compound **1** in the COX-2 pocket comprising of Site 1 and Site 2 supported the bidentate design of the molecules. The acridone part of the molecule was placed in the hydrophobic Site 1 whereas the pyrimidine fragment was seen extending towards Site 2. The MD of COX-2—**1**—heme complex provided more realistic picture showing the interaction of the pyrimidine moiety with water channel in Site 2 while acridone part was embedded in Site 1 (Fig. 6, S24). The molecules with 3C and 5C linker were not able to cover Site 1 and Site 2 simultaneously (supporting information). Further supporting the hypothesis, compound **5** carrying hydrophobic moieties at both the ends of the alkyl chain could not occupy the designated Site 1 and Site 2 when its molecular docking in COX-2 was performed.

### Synthesis of the compounds

**Scheme 1:**
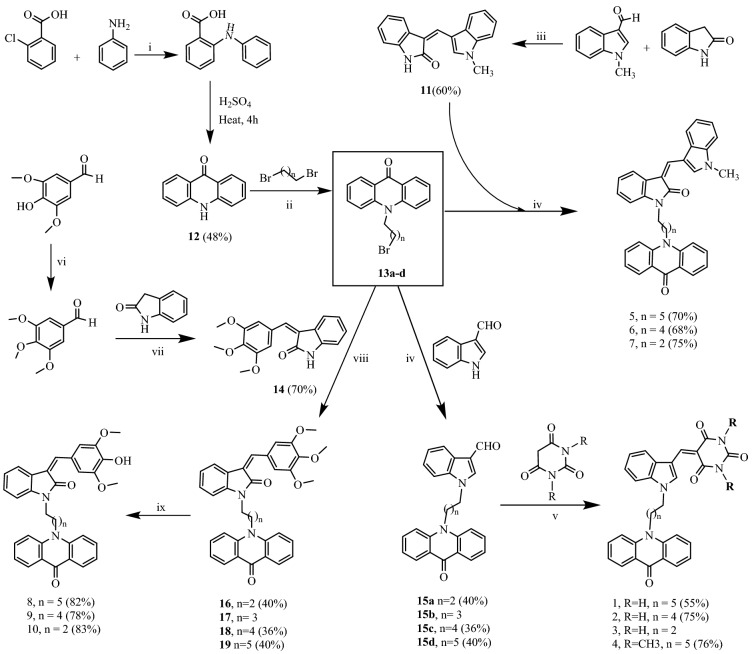
Reaction conditions: (i) K_2_CO_3_, CuO, iso-amyl alcohol, 150 °C, reflux, 36 h; (ii) NaH, DMF, 40 °C, 4 h; (iii) Piperidine, CHCl_3_, MWI, 120 °C, 20 min; (iv) NaH, ACN, 70 °C, 24 h; (v) DMF, MWI, 150 °C, 1 h; (vi) CH_3_I, K_2_CO_3_, DMF, 40 °C, 6 h; (vii) 145 °C, 15 min; (viii) K_2_CO_3_, DMF, 40 °C, 24 h; (ix) Anhydrous AlCl_3_, dry DCM, N_2_ atm, 2 h.

In order to validate the results of molecular modelling studies and developing new anti-inflammatory agents, the compounds were synthesized and screened through various biological experiments targeting COX-2. As depicted in Scheme 1, the formation of compound **12** from N-phenylanthranilic acid was followed by its reaction with dibromoalkanes in the presence of NaH in DMF to get compounds **13a–d**. Reaction of compounds **13a–d** with indole-3-carboxaldehyde using NaH as base in ACN afforded respective compounds **15a–d**. Further, the condensation of **15a–d** with barbituric acid and N,N′-dimethyl barbituric acid at 160 °C resulted into the formation of compounds **1**–**4** (Scheme 1). Reaction of compounds **13a**–**d** with **11** gave respective compounds **5**–**7**. Compound **14**, prepared from syringaldehyde and oxindole, was made to react with **13a**–**d** to get compounds **8**–**10** through the formation of compounds **16**–**19**.

### Biological studies

To demonstrate how the interplay of the proposed mechanism and design of the molecules influence their COX-2 inhibition and inflammation reversal, we performed the enzyme immunoassays and in vivo studies on animal models. All the compounds of Fig. 9 were screened for COX-1/2 inhibitory activity in triplicate at 10^–4^–10^–8^ M concentration by performing enzyme immunoassays^50^. The activity of the compounds was quantified in terms of amount of prostaglandins produced by the enzymes in the presence of different concentrations of the compound. Compound **1** showed appreciable potency against wt COX-2 with IC_50_ (50% inhibitory concentration) 0.06 µM whereas its IC_50_ for COX-1 was 60 µM exhibiting selectivity index 1,000 (Table 2). All the other compounds displayed poor COX-2 inhibitory activity (IC_50_ 0.1–48 μM) than that of compound **1** and the results were coinciding with the molecular docking studies data. Apparently, compound **1** that was interacting collectively in Site 1 and Site 2 proved more effective than those fitting in Site 1 only. In comparison to the four H-bond interactions of the pyrimidine part of compound **1** with the water channel, no such interaction was observed for compound **3** (Figure S32) and that may be the probable reason for poor activity of compound **3** than **1**. Compound **2** though exhibited interactions similar to that of **1** but did not show salt bridge formation with R120 (Figure S133). For compounds **4**, **5** and **6**, the acridone part of the molecules was more buried in the hydrophobic pocket of the enzyme (Figure S135–S137) though none of these molecules showed H-bond interactions except **6**. But compound **6** was engaged in one H-bond and one π–π interaction with the same residue Y355. For compounds **7**–**10**, the acridone component is shifted towards R120 (Figure S138–S141) but still some polar interactions of compounds **8** and **9** with W387 and R120 make them more potent than compounds **7** and **10**. Therefore, the trend of enzyme inhibitory activity of the compounds was quite in agreement with the extent of their H-bond and π–π interactions with the enzyme. These observations also supported the hypothesis that water channel around the heme do play certain role in the catalytic process of COX-2 and its disturbance affects the catalytic profile of the enzyme. The inflammation reversing activity, toxicity and mode of action of compound **1** confirming COX-2 as its cellular target was checked with the animal models.

**Table 2:** IC_50_ (μM) of the compounds for COX-2 and COX-1.

**Compound**	**IC**_**50**_ (μM)*		**SI (IC**_**50**_**(COX-1)/IC**_**50**_** (COX-2)**
	**wt COX-2**	**COX-1**	
1	0.06	60.00	1,000
2	0.10	40.00	400
3	10.00	> 100	> 10
4	2.15	33.00	15
5	0.15	53.00	350
6	0.35	60.00	160
7	22.00	> 100	> 4.5
8	0.12	48.00	400
9	0.23	52.00	250
10	12.00	> 100	> 8
13d	48.50	> 100	> 2
15d	23.48	> 100	> 4
Indomethacin	0.08	0.96	12
Celecoxib	0.04	15	375

*All the readings were average of 3 experiments with SD ± 0.5 µM.

### Analgesic and anti-inflammatory activity

Since it is known to produce biphasic pain response—the neurogenic pain and the inflammatory phase, 2% formalin injection was given in the right hind paw of the animals (20 µL) and the response was quantified by counting the number of flinching after 30 min of the compound **1** treatment^51^. The inflammatory phase has been reported in the current investigation since the cyclooxygenase inhibitors are known to ameliorate the latter phase. The involvement of cyclooxygenase and lipooxygenase pathways was studied by pretreatment with substance P and nitric oxide pathway was studied by pretreatment with L-arginine and L-NAME as previously described^51^. Anti-inflammatory activity was examined by using carageenan induced paw edema as per the previous report^52^.

**Figure 7:**
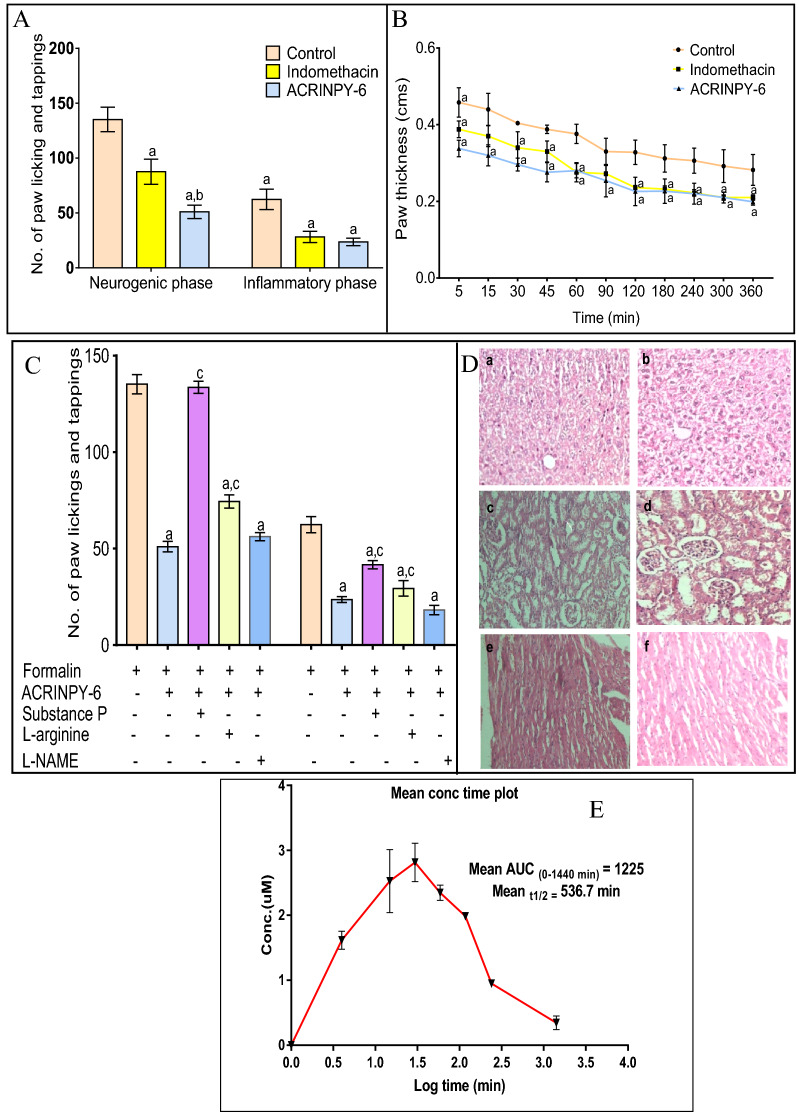
Effect of compound **1** on (**A**) formalin induced flinchings in neurogenic and inflammatory phases in mice, (**B**) paw thickness in carrageenan induced paw edema, (**C**) modulation of the analgesic effect of compound **1** by pre-treatment with substance P, L-arginine and L-NAME. All values are expressed as mean ± S.E.M. Statistical differences were determined by 2-way ANOVA followed by Tukey’s test. ^a^p < 0.05 *vs.* control, , ^b^p < 0.05 *vs.* Indomethacin, ^c^p < 0.05 *vs.* compound **1**. (**D**) Photomicrographs of hematoxylin–eosin (H & E) stained sections of (a) control liver, (b) **1** (2000 mg/kg) treated liver, (c) control kidney, (d) **1** treated kidney, (e) control myocardium, (f) **1** treated myocardium. All pictures were taken with light microscope at × 20 × magnifcation. (**E**) Pharmacokinetic profile of **1** indicated by concentration time plot.

A significant decrease in the number of flinchings in both the neurogenic and inflammatory phase of formalin induced pain was observed after the treatment of the animals with compound **1** (10 mg kg^−1^) (Fig. 7A). The effect was comparable to the standard drug indomethacin. Furthermore, the L-arginine pretreatment also attenuated the analgesic effect of **1** partially whereas L-NAME pretreatment did not (Fig. 7C). Substance P is known for stimulating the inducible cyclooxygenase and lipoxygenase pathway producing pain response due to an increased blood level of prostaglandins and leukotrienes^53^. Treatment of the animals with Substance P attenuated the analgesic effect of **1** but complete reversal was not evident, thereby indicating the possibility of linking some other factors in the analgesic effect of this compound. Increased production of nitric oxide by iNOS is known to cause pain and inflammation in animal studies^54,55^. The results of the current investigation revealed that L-arginine (NO precursor) pretreatment partially diminished the analgesic effect of **1** but the reversal was not complete. Carageenan injection produced a marked increase in the thickness of the injected paw. Indicating the anti-inflammatory activity of the compound, the treatment of the animals with **1** was found to produce a significant decrease in the paw volume after carageenan injection and the effect was comparable to that of the standard drug indomethacin (Fig. 7B).

Acute toxicity studies of **1** were carried out at 3 dose levels of 50 mg Kg^−1^, 300 mg Kg^−1^ and 2000 mg Kg^−1^ by periodic monitoring for the first 24 h and daily monitoring for 14 days^56^. Compound **1** was found to produce excessive licking, sniffing, grooming and restless movements for the first 60 min after oral administration. A few jumping movements were also observed. However, all these behaviours subsided after 60 min. No mortality was observed for the 14 days of observations. The histological examination of liver, kidney and myocardium was grossly unremarkable although a few areas of congestion were observed in the myocardium and kidney as compared to the vehicle treated control group (Fig. 7D). The pharmacokinetic (PK) profile of compound **1**, recorded as per the previously reported protocol^57^, showed half life (T_1/2_) ~ 9 h of compound **1** (Fig. 7E, Table 3). Therefore, complying with the design and the results of in vitro studies, the in vivo experiments were gestured with the emergence of a new category of anti-inflammatory agents.

**Table 3:** PK parameters of compound **1.**

**Parameter**	**Animal**			**Mean**
	**1**	**2**	**3**	
T_1/2_ (min)	671.3	417.8	520.9	536.7
T_max_ (min)	30	15	30	25
C_max_ (μM)	3.15	2.86	2.6	3.15
AUC_(0–1440 min)_	1,282	1,179.9	1,214	1,225.4

### HOMO–LUMO calculations supporting the working of the molecules

**Figure 8:**
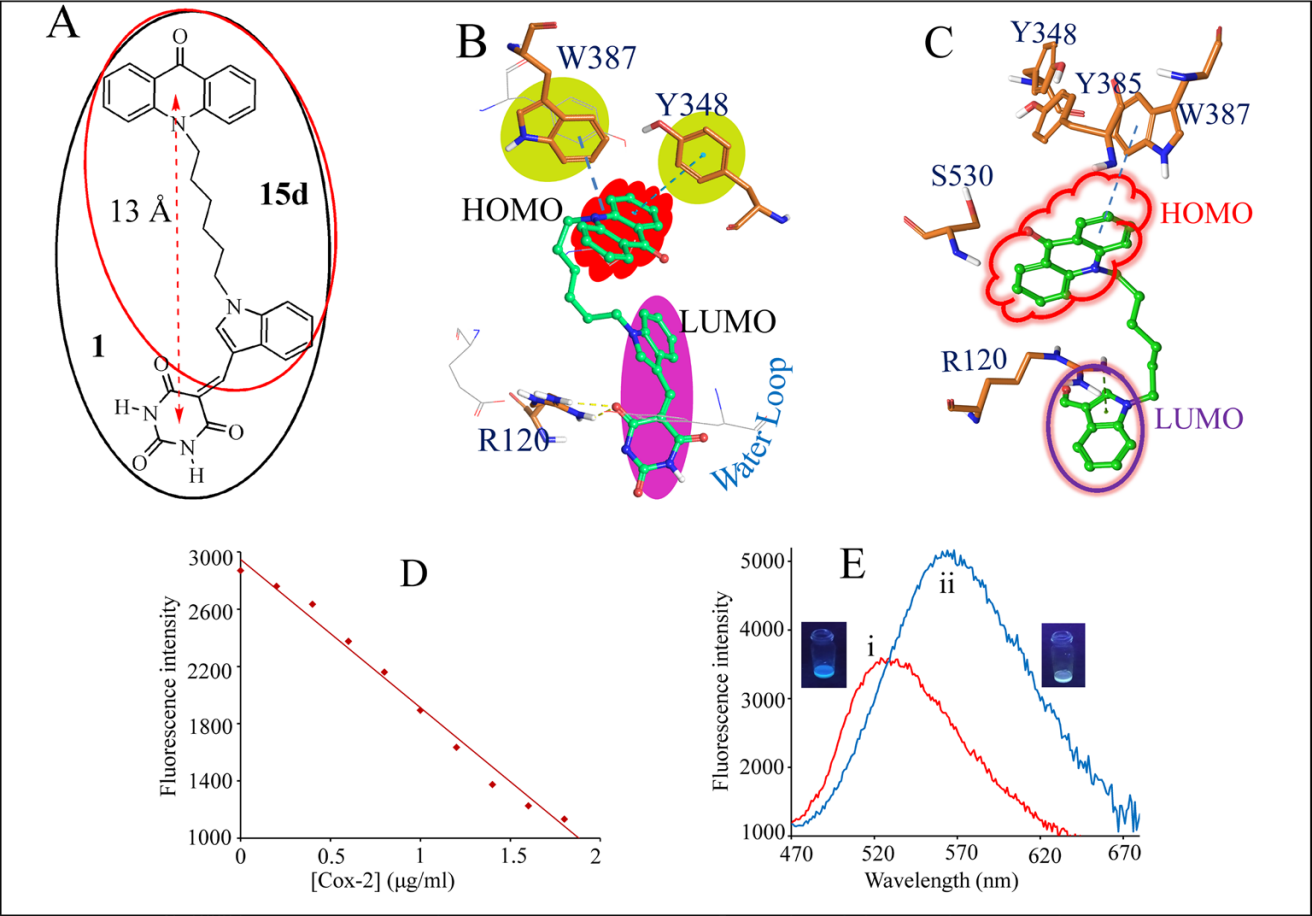
Fluorescence studies supporting the role of water loop: (**A**) Compounds **1** and **15d (15d** has CHO at C2-indole). HOMO–LUMO of (**B**) **1** and (**C**) **15d** interacting with COX-2. LUMO of **15d** did not interact with water loop. (**D**) Change in the fluorescence intensity of compound **1** at 567 nm Vs [COX-2] in H_2_O-DMSO (9:1, v/v). (**E**): Fluorescence spectra of: (i) **1** (1 μM) in the presence of lyophilised COX-2 in DMSO-H_2_O (9:1) and (ii) only **1** in DMSO-H_2_O (9:1, v/v). DMSO-H_2_O (9:1, v/v) solvent system was used to keep lyophilized enzyme as much as possible free of water. The concentration of the enzyme taken in DMSO-H_2_O (9:1, v/v) for fluorescence purpose was not sufficient to record the mass spectra.

**Figure 9:**
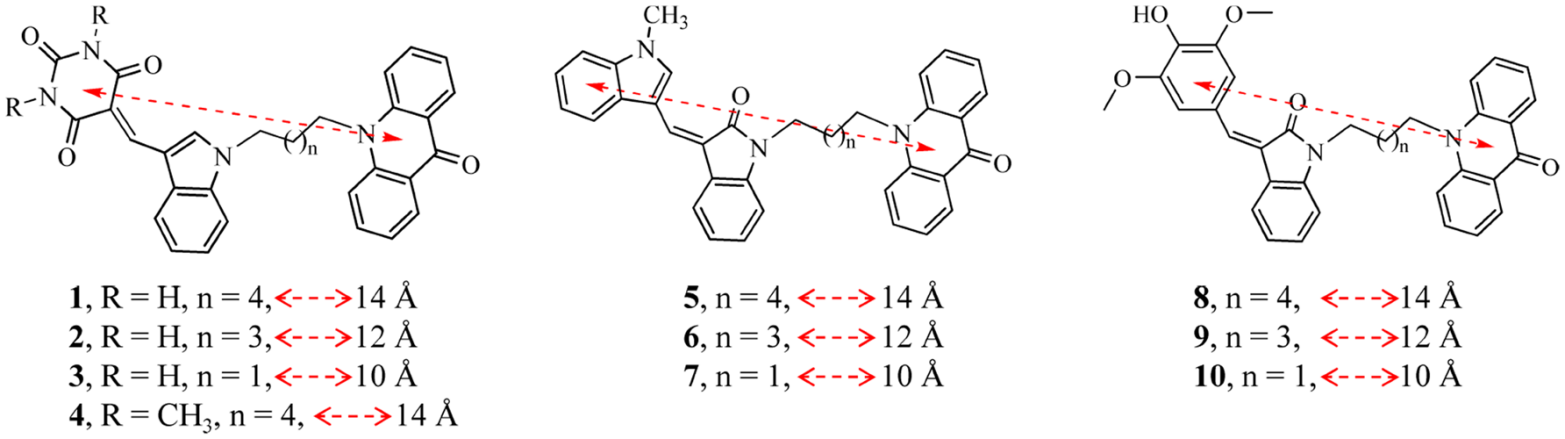
Rationally designed molecules for targeting the proposed electron transfer route and the AA binding site in cyclooxygenase.

The anti-inflammatory profile of compound **1** (Fig. 8A) was attributed to the appropriate distance between its polar and non-polar ends where the pyrimidine part entered the heme pocket disturbing the water loop and the acridine part interacts with Y385, W387, Y348 (Figure S24C, S24D). The location of the HOMO—LUMO maps of compound **1** (Fig. 8B) justified its fluorescence quenching in the presence of COX-2 (Fig. 8D, S25, S26) that probably has occurred due to the electron transfer between the (i) HOMO of **1** and the aromatic rings of W387 and Y348 as well as (ii) the LUMO of **1** and the water molecules around the heme. Partial change in the fluorescence of the solution of compound **1** in DMSO-H_2_O (9:1, v/v) on addition of lyophilised COX-2 (expected that all water associated with the enzyme is removed) (Fig. 8E), though a blue shift of 35 nm (probably due the solvent effect), was indicative of the role of water loop (besides the acridone–W387, Y348 interactions) in the fluorescence quenching of **1** by COX-2. In order to ensure the removal of all water in the lyophilised enzyme, we tried to record the mass spectra of the lyophilised COX-2 but not successful due to its poor solubility in non-aqueous medium and even not in the APCI mode (solid phase). Solution of **1** in DMSO-H_2_O (1:9, v/v) did not exhibit fluorescence quenching indicating that it is not the solvent water that is responsible for the fluorescence diminishing rather the COX-2 and its associated water molecules. Moreover, the decrease in the fluorescence intensity of compounds **13d** and **15d** (Scheme 1, Fig. 8A) (not having the pyrimidine moiety) in the presence of COX-2 was much less (Figure S27–S28) than that observed for compound **1** pointing also towards the contribution of the water loop in decreasing fluorescence through interaction with the LUMO of pyrimidine moiety (Fig. 8C, S29, S30). However, none of the other molecules in Fig. 9 and celecoxib disturbed the water loop rather they interact in the AA binding site only (Figure S31, S32).

## Conclusions

Although the H transfer processes are difficult to monitor in proteins but the variety of computer aided simulations tagged with the experimental evidences provided enough proof in favour of proton coupled electron transfer in cyclooxygenase. A loop of water molecules around the heme was identified whose one end coordinates with the Fe and the second end is H-bonded to N382. N382 is further connected to Y385 through T383, L384 making the probable route of proton translocation from Y385 to heme with the concomitant transfer of the electron from heme to Y385 during the metabolic phase of COX-2. The deciphered information for the role of water loop in the enzyme catalysis led to the design of new molecules interacting in the electron transfer channel as well as the AA binding pocket of the enzyme. Compound **1**, exhibiting interactions with both the sites, was the most effective anti-inflammatory agent amongst the 10 rationally designed molecules in the present studies. Compound **1** exhibited IC_50_ 0.06 μM against COX-2 and reversed the formalin induced analgesia and carageenan induced inflammation in mice by 90%. The results of these studies would have far-reaching effect in the design of new anti-inflammatory drugs and to look into the mechanism of other enzymes.

## Experimental

### General

Melting points were determined in capillaries and were uncorrected. ^1^H and ^13^C NMR spectra were recorded on Bruker 500 MHz and 125 MHz NMR spectrometer, respectively using CDCl_3_ and/or DMSO-*d*_6_ as solvent. Chemical shifts are given in ppm with TMS as internal reference. *J* values are given in Hertz. Signals are abbreviated as singlet, s; doublet, d; double-doublet, dd; triplet, t; multiplet, m. Mass spectra were recorded on Bruker micrOTOF Q II Mass spectrometer. The purity of the compounds was determined using q^1^HNMR method (Absolute q^1^HNMR with Internal Calibration, Figure S132)^58^. The spectral data of all the compounds are given in the supporting information. All in-vivo experiments were performed as per relevant guidelines and regulations and were approved by the institutional ethical committees: Institutional Animal Ethical Committee (IAEC) of Guru Nanak Dev University, Amritsar.

### Procedure for molecular dynamics (MD) simulations

For MD simulations, arachidonic acid was docked in the active site of COX-2 (pdb ID 3HS5)^26^ and the docked complex was optimized using MD simulations on Desmond module in Schrӧdinger Maestro version 10.1 with OPLS-2005 force field in the explicit solvent with TIP3P water model. The docked complex was placed adequately in TIP3P water molecules and the dimensions of each orthorhombic water box were 10 Å × 10 Å × 10 Å and for neutralising the system Na^+^ counter ions were added to balance the net charges of the systems and then 0.15 M NaCl was added. There were about 58,116 atoms of the generated solvent model for the docked complex. Before MD simulations, minimization and pre-equilibration of the system was done using default relaxation model in Desmond. NVT molecular dynamic simulations were performed at 10 K for 100 ps with restraints on heavy atoms. Then, the system was simulated for another 12 ps at 10 K with same settings. This was followed by NPT equilibration at 10 K for 12 ps. The system was simulated for 12 ps at 300 K with restraints on heavy atoms. Finally restraints on heavy atoms were removed and system was simulated for 24 ps at 300 K with thermostat relaxation time of 1 ps and barostat relaxation time of 2 ps. After minimization and equilibration, molecular dynamic simulations were performed at 300 K for 50 ns with Martyna-Tobias-Klein method. Data was collected every 1.5 ps during MD runs. The obtained 5,000 MD poses were analyzed.

### Energy calculations for the proposed radical carrier systems

In order to calculate the energies of the proposed transition states, single point energy task was performed using Jaguar, version 8.8, Schrӧdinger, LLC, New York, 2015. The proposed structures with radical localisation on particular atom were drawn successively in the workspace, which were taken as the input structures. Spin multiplicity and charge were taken according to the drawn structure i.e. spin treatment is spin restricted for closed shell molecules (RODFT) and spin un-restricted (UDFT) for open shell molecules. The basis set used was LACV3P** and level of theory was density functional theory (DFT) and the hybrid function used in these calculations was B3LYP. The method used for accelerating the convergence SCF calculations was Direct Inversion in the Iterative Subspace (DIIS) and no solvent model was taken so that all the calculations were applied to gas phase structure and hence energies were calculated for every system.

### Calculation of equilibrium constant (K_eq_)

In the present study, we calculated the energy of every system i.e. starting from the neutral system (no radical character on the heme) and moving on with the radical localisation at different sites-starting from heme to water loop and further to the respective peptide of channels ‘a’, ‘b’ and ‘c’ and finally to Y385–OH. From the energy values, we calculated the enthalpy change ∆H. By taking the assumption that the whole system is in equilibrium we are taking ∆S = 0.

From the Gibbs free energy equation

$$\Delta {\text{G }} = \, \Delta {\text{H }} - {\text{ T}}\Delta {\text{S}}{.}$$

Under standard conditions

$$\Delta {\text{G}}^{ \circ } = \, \Delta {\text{H}}^{ \circ } - {\text{ T}}\Delta {\text{S}}^{ \circ } .$$

Here, ∆G° = standard change in Gibbs free energy, ∆H° = Standard Change in enthalpy.

T = temperature i.e. 298 K, ∆S° = standard change in entropy.

At equilibrium, ∆S° = 0

$$\Delta {\text{G}}^{{\text{o}}} = \, \Delta {\text{H}}^{{\text{o}}} .$$

Also, ∆G = ∆G° + RT lnK_eq_.

Again at equilibrium, ∆G = 0.

So, ∆G° = -RT lnK_eq_.

Now, K_eq_ = e^-∆Go/RT^.

R = universal gas constant (1.9872 cal K^-1^ mol^-1^), K_eq_ = equilibrium constant.

### Procedure for calculating p*K*_a_

The p*K*_a_ calculations were performed using Jaguar, version 8.8, Schrӧdinger, LLC, New York, 2015. A series of calculations on the protonated form and on the deprotonated form of each of the participating group of the three channels were performed followed by an empirical correction. The atom or atoms whose p*K*_a_ values are calculated were specified in p*K*_a_ atom cell in the input tab. p*K*_a_ atom was selected in such a manner that it should be the acidic hydrogen atom in an acid, or the basic atom in a base. The method used for accelerating the convergence SCF calculations was Direct Inversion in the Iterative subspace (DIIS) at 2000 iterations and water was selected as the solvent. After finishing the settings, job was run and after the job completion, p*K*_a_ values for each atom were added to the structure in the maestro output file.

Five participating groups of channel ‘a’, six participating groups of channel ‘b’ and seven groups of channel ‘c’ were selected one by one in the protonated as well as the deprotonated form for calculating their respective p*K*_a_ (Figure S11). The p*K*_a_ for the deprotonated atoms are shown in Table 1 in the main text.

### Enzyme activity of the wt and mu COX-2

The arachidonic acid metabolic activity of the wt- as well as the mu- COX-2 was checked by the enzyme immunoassay kits^50^. The recombinant proteins were purchased from Merck, Sigma and Cayman Chemical Company. All the reactions of the enzyme immunoassays were performed as per the kit protocol except the use of various mu COX-2. The activity of the mu COX-2 w.r.t. the wt COX-2 was determined by calculating the amount of PGE2 generated by each mu COX-2 and comparing with the PGE2 generation by wt COX-2. UV–vis spectra were recorded on BIOTEK Synergy H1 Hybrid Reader.

### *K*_m_ calculations

The Michaelis Menten constants of each of the mu COX-2 as well as the wt COX-2 for arachidonic acid were determined by monitoring the formation of prostaglandins as a function of arachidonic acid concentration. All the experiments were performed at pH 7.0 as per the protocol of enzyme immunoassay kit^50^ but the concentration of arachidonic was varied and the formation of prostaglandins was noted. 1/V_o_ and 1/[AA] were determined from the absorbance spectrax^59^. K_m_ was determined from the slop of the plot between 1/V_o_ and 1/[AA]. K_m_ = slope x V_max_ where V_max_ was intercept on y- axis.

### Procedure for COX inhibitory immunoassay

For studying the COX-1, COX-2 inhibitory activities of the compounds, various reagents were prepared as per the protocol of the assays^50^. The compounds were screened in triplicate at 10^–4^–10^–8^ M concentration using the procedure as described in the previous report^52^. The concentrations of the compound **1** causing 50% inhibition (IC_50_) were determined using a dose response inhibition curve (duplicate determinations) with GraphPad PRISM.

### Analgesic and anti-inflammatory activity

Swiss albino mice of either sex (25–35 g) were used for determining the analgesic and anti-inflammatory activity of the compounds. The animals were maintained at 22 ± 2 °C under 12 h light/12 h dark cycle with free supply of food and water. The study was approved by the IAEC of Guru Nanak Dev University, Amritsar, Punjab, India. A total of six groups of animals with 5 animals in each group were used. The experimental procedure as described in our previous report^52,57^ was used for studying the analgesic and anti-inflammatory activity of the compounds. All the treatments were given intraperitoneally (i.p.).

*Mechanistic studies*. Three groups of mice with 5 animals in each group were taken for exploring the inhibition of COX and LOX and modulation of nitric oxide pathway. For COX and LOX pathways, the animals were pretreated with substance P, 30 min before administering **1**. For nitric oxide pathway, animals were pretreated with nitric oxide precursor, L-arginine and NOS inhibitor, L-NAME, 30 min before administering **1**. The details of the protocols are described in previous reports^52,57^.

*Acute Toxicity Studies*^56^. Four groups of animals with three animals per group were taken. The first group was administered the vehicle and served as the control group, the second, third and the fourth groups were treated with **1** at doses of 50 mg Kg^−1^, 300 mg Kg^−1^ and 2000 mg Kg^−1^, respectively. All the treatments were administered after 4 h of fasting. Thereafter, the animals were observed continuously for the first four hours and periodically for 24 h. After 14 days, one animal each in control and highest dose of **1** (2000 mg Kg^−1^) was sacrificed and histological studies were conducted using H and E staining.

**In-vivo Pharmacokinetic studies** were performed as per the previously reported protocol^57^.

### UV–Vis and fluorescence studies

In order to support in-silico experiments, the interactions of **1**, **13d** and **15d** with COX-2 were checked with UV–vis and fluorescence spectral techniques. The UV–Vis spectrum of **1** at 1 µM concentration in Tris–HCl buffer (pH 7.25) exhibited absorption bands at 255 and 420 nm. Incremental addition of COX-2 to the solution of **1** resulted in the absorbance decrease at 255 and 420 nm indicating interactions of the compound with COX-2. The appearance of level-off in the visible region (500–700 nm) was attributed to the Mie scattering due to the formation of aggregates. The fluorescence spectrum of 0.5 µM solution of **1** in Tris–HCl buffer (pH 7.25) exhibited emission band at 567 nm when excited at 420 nm. Upon addition of COX-2 to the aqueous solution of the compound, there was significant quenching in fluorescence emission. Corroborating the results of molecular modelling studies, the changes in the UV–Vis spectra as well as the fluorescence spectrum of the compound on addition of COX-2 probably occurred due to the HOMO–LUMO interactions between **1** and the enzyme (Figure S26). The linear Stern–Volmer plot of decrease in fluorescence intensity at 567 nm on increasing COX-2 concentration gave Stern–Volmer constant Ksv 3.30 × 10^4^ M^-1^. The detection limit of the compound for COX-2 was 0.02 nM (Figure S26C,D).

### HOMO–LUMO analysis

Keeping in view the hydrophobic and hydrophilic interactions of the molecule through acridone and pyrimidine moieties, respectively, the HOMO–LUMO analysis of the ligand and the enzyme–substrate/ligand complex was performed so that the change in fluorescence of compound **1**, **13d** and **15d** in the presence of COX-2 was justified. The geometry optimization and calculations for the compounds were performed by using density functional theory (DFT) level of Jaguar-Schrodinger. The highest occupied molecular orbital (HOMO) and the lowest unoccupied molecular orbital (LUMO) energies at B3LYP/6-31G** level were calculated. PBF solver was used for optimization of structure in both the gaseous and solution phase. In compound **1**, HOMO maps were located on the acridine part of the compound. Docking studies of compound **1** with COX-2 also showed the involvement of acridine moiety in protein–ligand interactions. The LUMO maps were located on the indole-pyrimidine moiety of the compound. As predicted from the molecular modelling studies, the acridone part of compound **1** is placed in the hydrophobic pocket of COX-2 and it exhibits π–π interactions with Y348, W385 and W387; the transfer of electron between HOMO of acridone and the Y348/W385/W387 may be responsible for the quenching of fluorescence of compound **1** in the presence of the enzyme. Moreover, LUMO of **1** also interact with the water loop. While HOMO of **13d** and **15d** do interact with W387 but their LUMO did not interact with the water loop. The lowest unoccupied molecular orbital/highest occupied molecular orbital (LUMO/HOMO) energy gaps ΔEg for compound **1** were calculated (Table S4, Figure S25).

## Supplementary information

Supplementary file1

Supplementary file2

Supplementary file3

Supplementary file4

Supplementary file5

Supplementary file6

Supplementary file7

Supplementary file8

Supplementary file9

Supplementary file10

Supplementary file11

## Abbreviations

ACN: Acetonitrile
AA: Arachidonic acid
COX: Cyclooxygenase
MD: Molecular dynamics
RSE: Radical stabilization energy
PK: Pharmacokinetic
RMSD: Root mean square deviation
DMSO: Dimethyl sulphoxide
HOMO: Highest occupied molecular orbital
LUMO: Lowest unoccupied molecular orbital
PCET: Proton coupled electron transfer
mu: Mutant
wt: Wild type

## References

[1] Vane JR, Botting RM (1998). Anti-inflammatory drugs and their mechanism of action. Inflamm. Res..

[2] Wilerson JT, Ridker PM (2004). Inflammation as a cardiovascular risk factor. Circulation.

[3] Coussens LM, Werb Z (2002). Inflammation and cancer. Nature.

[4] Schaible H-G, Ebersberger A, Banchet GSV (2002). Mechanisms of pain in arthritis. Ann. N. Y. Acad. Sci..

[5] Skaper SD, Facci L, Zusso M, Giusti P (2018). An inflammation-centric view of neurological disease: Beyond the neuron. Front Cell. Neurosci..

[6] Lontchi-Yimagou E, Sobngwi E, Matsha TE, Kengne AP (2013). Diabetes mellitus and inflammation. Curr Diab Rep..

[7] DuBois RN, Abramson SB, Crofford L, Gupta RA, Simon LS, Putte LBAVD, Lipsky PE (1998). Cyclooxygenase in biology and disease. FASEB J..

[8] Seibert K, Maferrer JL (1994). Role of inducible cyclooxygenase (COX-2) in inflammation. Receptor..

[9] Tanabe T, Tohnai N (2002). Cyclooxygenase isozymes and their gene structures and expression. Prostaglandins Other Lipid Mediat..

[10] Smith WL, Langenbach R (2001). Why there are two cyclooxygenase isozymes. J. Clin. Invest..

[11] Turini ME, DuBois RN (2002). Cyclooxygenase-2: A therapeutic target. Annu. Rev. Med..

[12] Smith WL, Urade Y, Jakobsson P-J (2011). Enzymes of the cyclooxygenase pathways of prostanoid biosynthesis. Chem. Rev..

[13] Penning TD, Talley JJ, Bertenshaw SR, Carter JS (1997). Synthesis and biological evaluation of the 1,5-diarylpyrazole class of cyclooxygenase-2 inhibitors: Identification of 4-[5-(4-methylphenyl)-3-(trifluoromethyl)- 1*H*-pyrazole-1-yl]benzenesulphonamide (SC-58635, celecoxib). J. Med. Chem..

[14] Prasit P, Wang Z, Brideau C, Chan C-C (1999). The discovery of rofecoxib, [MK 966, vioxx, 4-(4- methylsulphonylmethyl)-3-phenyl-2(5*H*)-furanone], an orally active cyclooxygenase-2 inhibitor. Bioorg. Med. Chem. Lett..

[15] Talley JJ, Brown DL, Carter JS, Graneto MJ, Koboldt CM, Masferrer JL, Perkins WE, Rogers RS, Shaffer AF, Zhang YY, Zweifel BS, Seibert K (2000). 4-[5-methyl-3-phenylisoxazol-4-yl]-benzenesulphonamide, valdecoxib: A potent and selective inhibitor of COX-2. J. Med. Chem..

[16] Miedany YE, Youssef S, Ahmed I, Gaafary ME (2006). The gastrointestinal safety and effect on disease activity of etoricoxib, a selective Cox-2 inhibitor in inflammatory bowel diseases. Am J. Gastroenterol..

[17] Tseng T-S, Chuang S-M, Hsiao N-W, Chen Y-W, Lee Y-C, Lin C-C, Huanga C, Tsai K-C (2016). Discovery of a potent cyclooxygenase-2 inhibitor, S4, through docking-based pharmacophore screening, in vivo and in vitro estimations. Mol. BioSyst..

[18] William L, Smith WL, Eling TE, Kulmacz RJ, Marnett LJ, Tsaii A-H (1992). Tyrosyl radicals and their role in hydroperoxide-dependent activation and inactivation of prostaglandin endoperoxide synthase. Biochemistry.

[19] Shimokawa T, Kulmacz RJ, Dewitt DL, Smith WL (1990). Tyrosine 385 of prostaglandin endoperoxide synthase is required for cyclooxygenase catalysis. J. Biol. Chem..

[20] Marnett LJ (2000). Cyclooxygenase mechanisms. Curr. Opin. Chem. Biol..

[21] Dorlet P, Seibold SA, Babcock GT, Gerfen GF, Smith WL, Tsai AL, Un S (2002). High-field EPR study of tyrosyl radicals in prostaglandin H2 synthase-1. Biochemistry.

[22] Donk WAVD, Tsai A-L, Kulmacz RJ (2002). The cyclooxygenase reaction mechanism. Biochemistry.

[23] Rouzer CA, Marnett LJ (2003). Mechanism of free radical oxygenation of polyunsaturated fatty acids by cyclooxygenases. Chem. Rev..

[24] Yu Q, Purwaha P, Ni K, Sun C, Mallik S, Qian SY (2009). Characterization of novel radicals from COX-catalyzed arachidonic acid peroxidation. Free Radic Biol. Med..

[25] Liu Y, Roth J (2016). A revised mechanism for human cyclooxygenase-2. J. Biol. Chem..

[26] Vecchio AJ, Simmons DM, Malkowski MG (2010). Structural basis of fatty acid substrate binding to cyclooxygenase-2. J. Biol. Chem..

[27] Kohen A, Klinman JP (1998). Enzyme catalysis: Beyond classical paradigms. Acc. Chem. Res..

[28] Scrutton NS, Basran J, Sutcliffe MJ (1999). New insights into enzyme catalysis ground state tunnelling driven by protein dynamics. Eur. J. Biochem..

[29] Ji, S. In. *Planckian distributions in molecular machines, living cells, and brains: The wave-particle duality in biomedical sciences*, Proc. Intern. Conf. on Biology and Biomedical Engineering,Vienna, March 15–17, 2015; pp. 115–137.

[30] Masgrau L, Roujeinikova A, Johannissen LO, Hothi P, Basran J, Ranaghan KE, Mulholland AJ, Sutcliffe MJ, Scrutton NS, Leys D (2006). Atomic description of an enzyme reaction dominated by proton tunneling. Science.

[31] Ball P (2004). By chance, or by design?. Nature.

[32] Danish HH, Doncheva IS, Roth JP (2011). Hydrogen tunneling steps in cyclooxygenase-2 catalysis. J. Am. Chem. Soc..

[33] Loftfield RB, Eigner EA, Pastuszyn A, Jakubowski H (1980). Conformational change during enzyme catalysis: Role of water in the transition state. Proc. Natl. Acad. Sci. USA.

[34] Pocker Y (2000). Water in enzyme reactions: Biophysical aspects of hydration and dehydration processes. Cell. Mol. Life Sci..

[35] Sage CR, Rutenber EE, Stout TJ, Stroud RM (1996). An essential role for water in an enzyme reaction mechanism: The crystal structure of the thymidylate synthase mutant E58Q. Biochemistry.

[36] Knight JDR, Hamelberg D, McCammon JA, Kothary R (2009). The role of conserved water molecules in the catalytic domain of protein kinases. Proteins Struct. Funct. Bioinf..

[37] Wang X, Hirao H (2013). ONIOM (DFT: MM) study of catalytic mechanism of *myo*-inositol monophosphatase: Essential role of water in enzyme catalysis in two-metal mechanism. J. Phys. Chem. B..

[38] Chen M, Chou WKW, Al-Lami N, Christianson DW (2016). Probing the role of active site water in the sesquiterpene cyclization reaction catalyzed by Aristolochenesynthase. Biochemistry.

[39] Dance I (2018). What is the role of isolated small water pool near FeMo-co, the active site of nitrogenase?. The FEBS Journal.

[40] Vidossich P, Fiorin G, Alfonso-Prieto M, Rovira C (2010). On the role of water in peroxidase catalysis: A theoretical investigation of HRP compound I formation. J. Phys. Chem. B..

[41] Shrimpton P, Allemann RK (2002). Role of water in catalytic cycle of *E. coli* dihydrofolate reductase. Prot. Sci..

[42] Manna RN, Malakar T, Jana B, Paul A (2018). Unraveling the crucial role of single active water molecule in the oxidative cleavage of aliphatic C-C bond of 2,4’- dihydroxyacetophenone catalysed by 2,4’-dihydroxyacetophenone dioxygenase enzyme: A quantum mechanics/molecular mechanics investigation. ACS Catal..

[43] Lee HJ, Svahn E, Swanson JMJ, Gennis RB (2010). Intricate role of water in proton transport through cytochrome c oxidase. J. Am. Chem. Soc..

[44] Dasgupta S, Mukherjee S, Mukhopadhyay BP, Mishra DK (2017). Recognition dynamics of dopamine to human monoamine oxidase B: role of Leu171/Gln206 and conserved water molecules in the active site cavity. J. Biomol. Struct. Dyn..

[45] Hammes-Schiffer S, Stuchebrukhov AA (2010). Theory of coupled electron and proton transfer reactions. Chem. Rev..

[46] Weinberg DR, Gagliardi CJ, Hull JF, Murphy CF, Kent CA, Westlake BC, Paul A, Ess DH, McCafferty DG, Meyer TJ (2012). Proton-coupled electron transfer. Chem. Rev..

[47] Reece SY, Nocera DG (2009). Proton-coupled electron transfer in biology: results from synergistic studies in natural and model systems. Annu. Rev. Biochem..

[48] Bambai B, Rogge CE, Stec B, Kulmacz RJ (2004). Role of Asn-382 and Thr-383 in activation and inactivation of human prostaglandin H synthase cyclooxygenase catalysis. J. Biol. Chem..

[49] Wu G, Kulmacz RJ, Tsai A-L (2014). Kinetic isotope effect of Prostaglandin H synthase exhibits inverted temperature dependence. Catalysts.

[50] COX inhibitor screening assay kit (item no. 560131) was purchased from Cayman Chemical Co. and the standard protocols, as supplied with assay kits, were followed for evaluating inhibitory activities.

[51] Hunskaar S, Hole K (1987). The formalin test in mice: dissociation between inflammatory and non-inflammatory. Pain.

[52] Singh P, Kaur S, Kaur J, Singh G, Bhatti R (2016). Rational design of small peptides for optimal inhibition of cyclooxygenase-2 (COX-2): Development of a highly effective anti-inflammatory agent. J. Med. Chem..

[53] Castellani ML, Conti P, Felaco M, Vecchiet J, Ciampoli C, Cerulli G, Boscolo P, Theoharides TC (2009). Substance P upregulates LTB4 in rat adherent macrophages from granuloma induced by KMnO_4_. Neurotox. Res..

[54] Koon HW, Zhao D, Zhan Y, Rhee SH, Moyer MP, Pothoulakis C (2006). Substance P stimulates cyclooxygenase-2 and prostaglandin E2 expression through JAK-STAT activation in human colonic epithelial cells. J. Immunol..

[55] Ge Z-J, Tan Y-F, Zhao Y-P, Cui G-X (2008). Evidence that inhibition of spinal nitric oxide production contributes to the Antinociceptive effects of emulsified isoflurane on formalin induced pain in rats. Clin. Exp. Physiol. Pharmacol..

[56] OECD Guideline for testing of chemicals, 2001. Guideline 423: acute oral toxicity- acute toxic class method, 2001.

[57] Kaur B, Kaur M, Kaur N, Garg S, Bhatti R, Singh P (2019). Engineered substrate for cyclooxygenase-2: A pentapeptide isoconformational to arachidonic acid for managing inflammation. J. Med. Chem..

[58] Pauli GF, Chen S-N, Simmler C, Lankin DC, Godecke T, Jaki BU, Friesen JB, McAlpine JB, Napolitano JG (2014). Importance of purity evaluation and the potential of quantitative ^1^H NMR as a purity assay. J. Med. Chem..

[59] Singh H, Kaur B, Kaur H, Singh P (2018). A bisubstrate reagent orchestrating adenosine triphosphate and L-tyrosine and making tyrosyl adenylate: partial mimicking of tyrosyl-tRNA synthetase. Org. Biomol. Chem..

