# Supplementary material for: Role of water in cyclooxygenase catalysis and design of anti-inflammatory agents targeting two sites of the enzyme
Source: Sci Rep. 2020 Jul 1;10:10764. doi: 10.1038/s41598-020-67655-6 (PMC7329864; doi:10.1038/s41598-020-67655-6)
Supplement: Supplementary file 2 — Supplementary file2 [file 41598_2020_67655_MOESM2_ESM.doc]

**SUPPORTING INFORMATION**

**Role of Water in Cyclooxygenase Catalysis and Design of Anti-inflammatory Agents Targeting Two Sites of the Enzyme**

Manpreet Kaur^ψ^, Baljit Kaur^ψ^, Jagroop Kaur^ψ^, Anudeep Kaur^Ф^, Rajbir Bhatti^Ф^, Palwinder Singh*^ψ^

^ψ^Department of Chemistry and ^Ф^Department of Pharmaceutical Sciences, University with Potential for Excellence – Guru Nanak Dev University, Amritsar-143005. India

**Table of contents**

| **S. No.** | **Contents** | **Page no.** |
| --- | --- | --- |
| 1 | MD simulations | S2-S3 |
| 2 | Figure S5, S6 | S4 |
| 3 | Energy calculations, Table S1, Table S2 | S5-S14 |
| 4 | Procedure for calcualting p*K*_a_ | S14-S15 |
| 5 | UV spectra studies, Figure S11 | S15-S16 |
| 6 | Figure S12, S13 | S18 |
| 7 | p*K*_a_, *K*_m_ calcualtions | S18-S22 |
| 8 | Table S3, Figure S24 | S23 |
| 9 | HOMO-LUMO, UV-vis, Fluorescecne spectra, Figure S25-S30 | S24-S28 |
| 10 | Figure S31, S32 | S29 |
| 11 | Synthesis of molecules, experimental data, NMR, mass, IR spectra | S30-S81 |
| 12 | Percentage purity of compound **1** by q^1^H NMR | S81-S82 |
| 13 | Molecular Docking Figures | S82-S86 |
| 14 | References | S86-S87 |

**
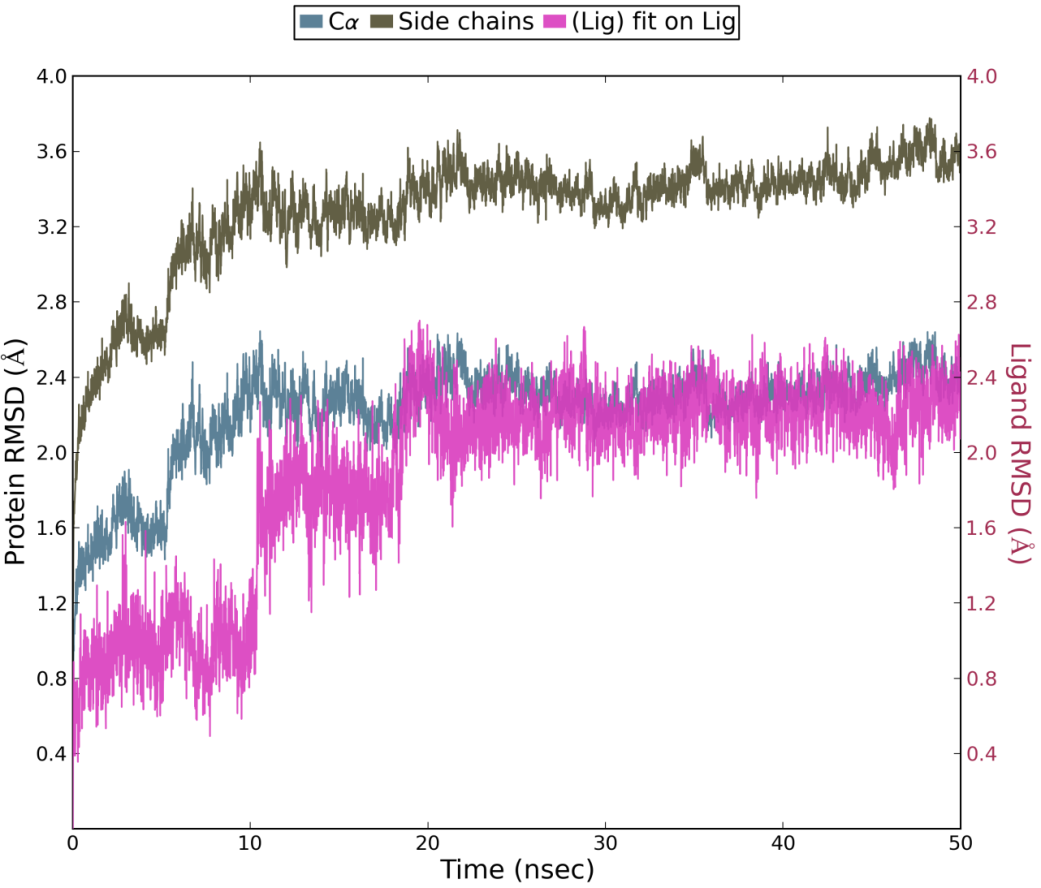
**

**Figure S1**. Root Mean square deviation (RMSD) of backbone atoms during evolution of trajectory of Cα (blue), side chain of protein (brown) and atoms of ligand arachidonic acid (pink) are shown.

**
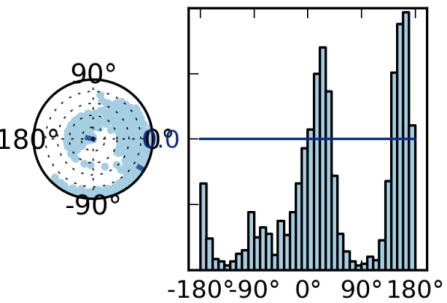

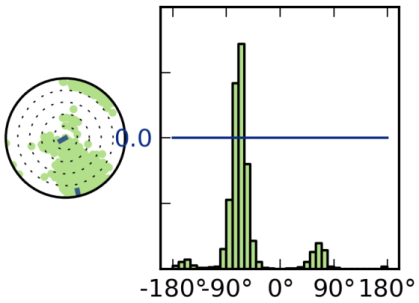

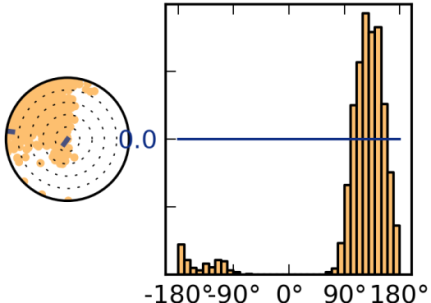

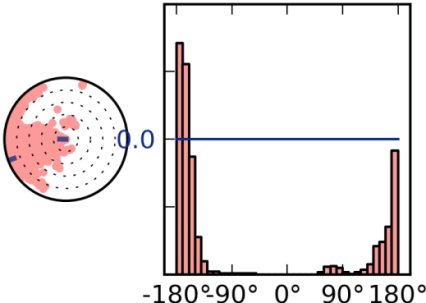

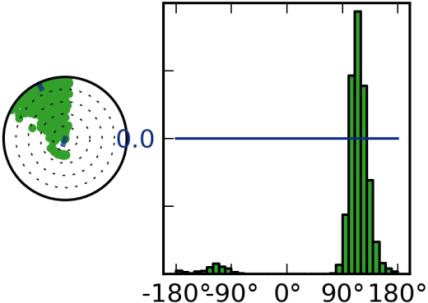

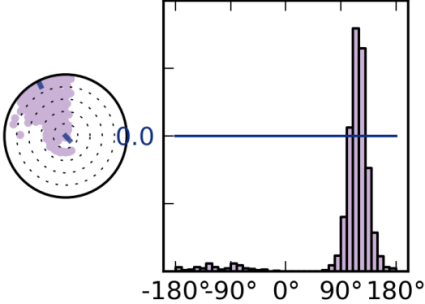

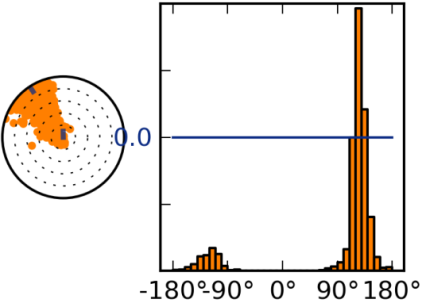

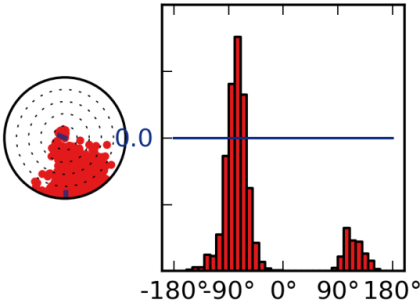

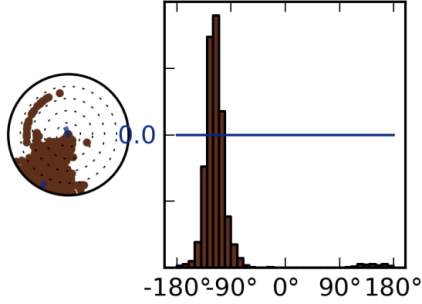

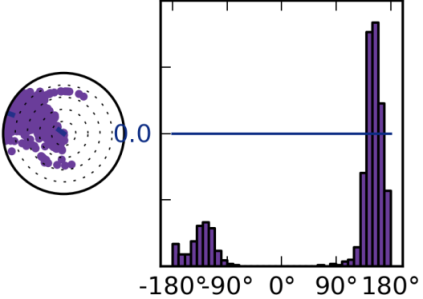
**

**
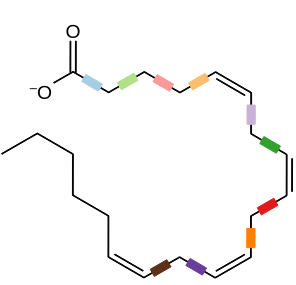
**

**Figure S2**. Ligand torsions plots summarize the conformational evolution of every rotatable bond in the ligand throughout the simulation trajectory (0.00-50 ns). Each rotatable bond torsion is accompanied by a dial plot and bar plots of same color.

**
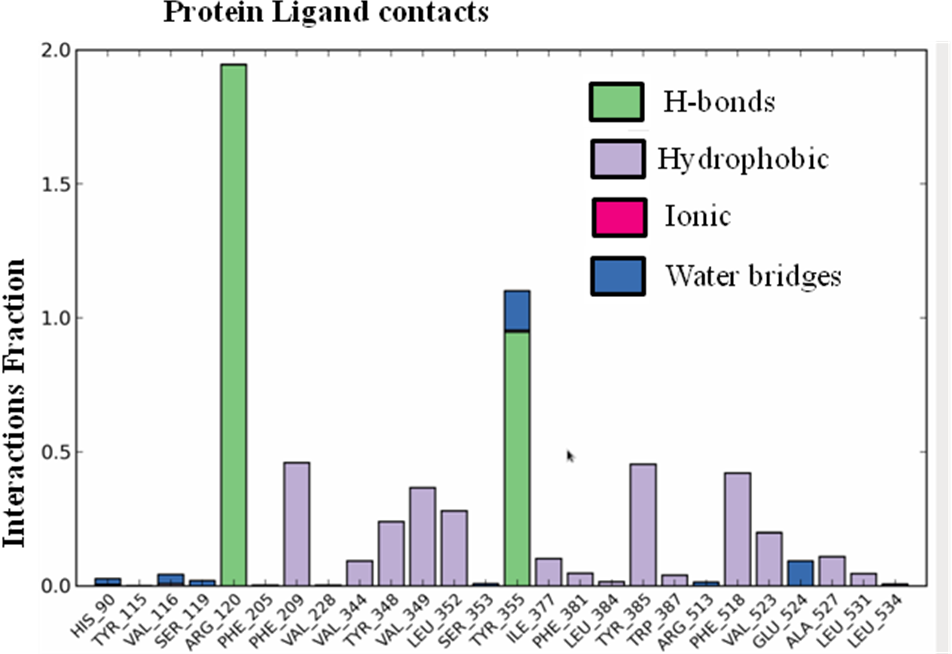
**

**Figure S3**. Interaction analysis between protein and ligand (AA) throughout the simulation over the period of 0.00-50 ns. The stacked bar charts were normalised throughout the trajectory.

**
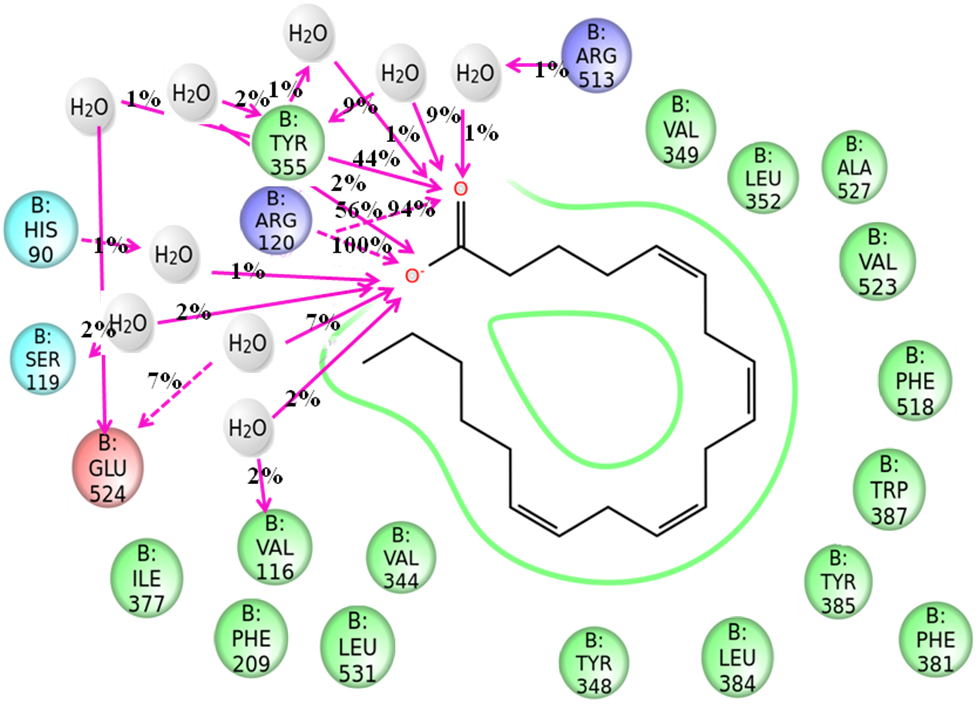
**

**Figure S4**. Detail representation of arachidonic acid atoms with protein residues during the evolution of trajectory (0.00-50 ns) that occur more than 1%.

**
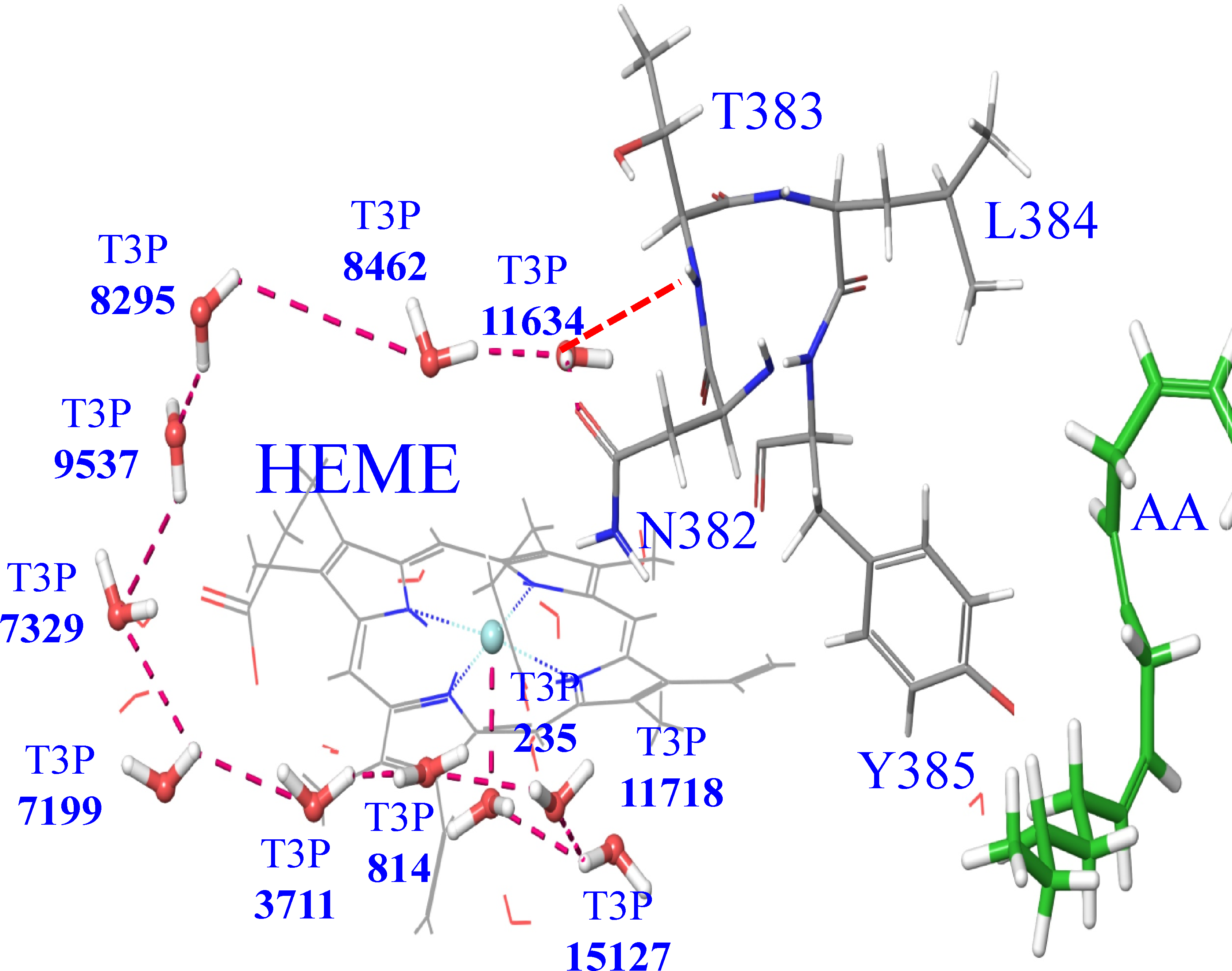
**

**Figure S5**. MD of COX-2 – AA – Heme complex showing the numbering of water molecules around the heme.

**
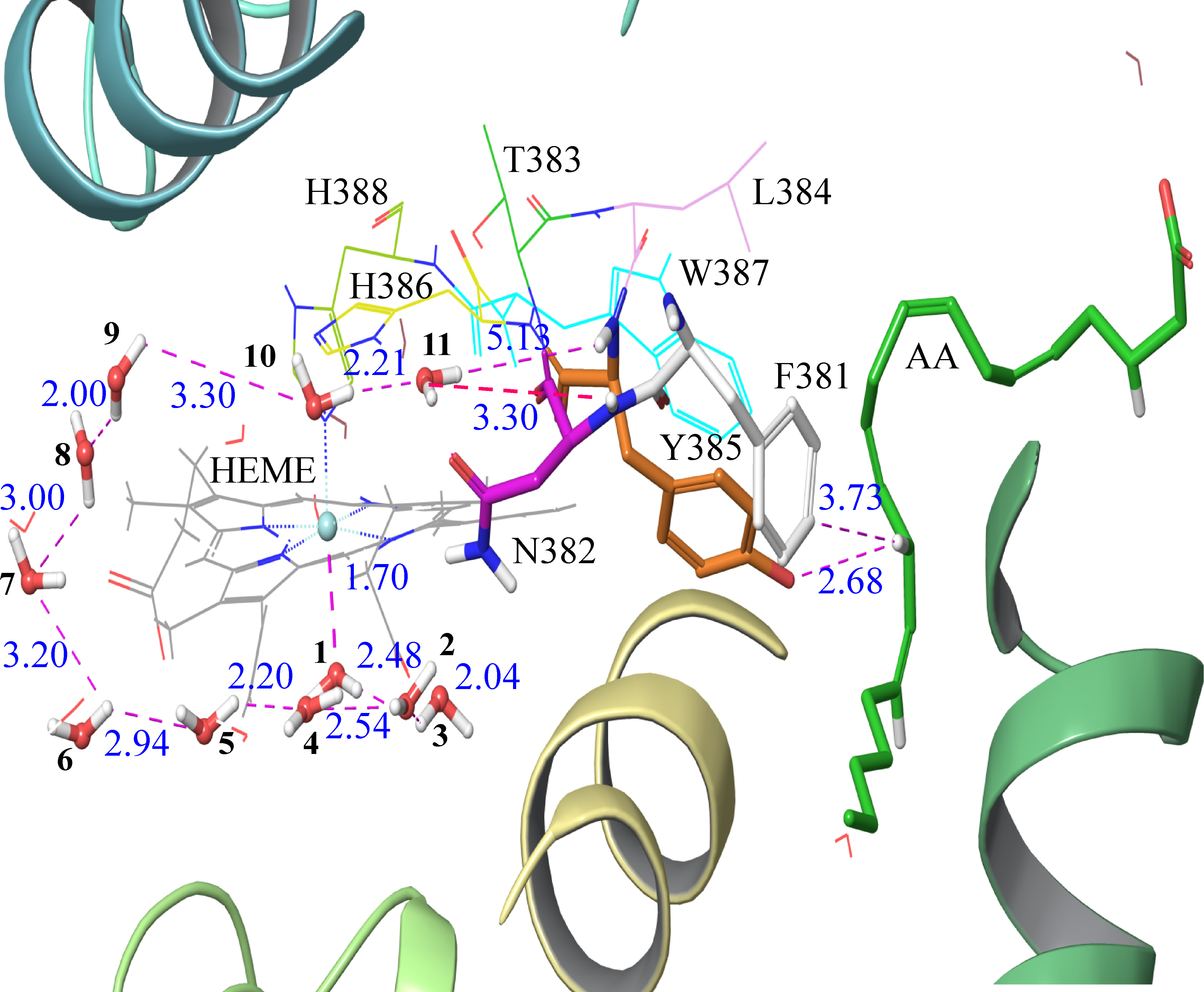
**

**Figure S6**. MD of COX-2 – AA – heme complex showing the distance between the water molecuels of the loop.

**Energy Calculations for the proposed radical carrier systems**

In order to calculate the energies of the proposed transition states, single point energy task was performed using Jaguar, version 8.8, Schrӧdinger, LLC, New York, 2015. The proposed structures with radical localisation on particular atom were drawn successively in the workspace, which were taken as the input structures. Spin multiplicity and charge were taken according to the drawn structure i.e. spin treatment is spin restricted for closed shell molecules (RODFT) and spin un-restricted (UDFT) for open shell molecules. The basis set used was LACV3P** and level of theory was density functional theory (DFT) and the hybrid function used in these calculations was B3LYP. The method used for accelerating the convergence SCF calculations was Direct Inversion in the Iterative Subspace (DIIS) and no solvent model was taken so that all the calculations were applied to gas phase structure and hence energies were calculated for every system.

**Calculation of equilibrium constant (K_eq_)**^1^

In the present study, we calculated the energy of every system i.e. starting from the neutral system (no radical character on the heme) and moving on with the radical localisation at different sites- starting from heme to water loop and further to the respective peptide of channels ‘a’, ‘b’ and ‘c’ and finally to Y385 –OH. From the energy values, we calculated the enthalpy change ∆H. By taking the assumption that the whole system is in equilibrium we are taking ∆S = 0.

From the Gibbs free energy equation

∆G = ∆H - T∆S

Under standard conditions

∆G^o^ = ∆H^o^ - T∆S^o^

Here, ∆G^o^ = standard change in Gibbs free energy, ∆H^o^= Standard Change in enthalpy

T = temperature i.e. 298 K, ∆S^o^ = standard change in entropy

At equilibrium, ∆S^o^ = 0

∆G^o^ = ∆H^o^

Also, ∆G = ∆G^o^+ RT lnK_eq_

Again at equilibrium, ∆G = 0

So, ∆G^o^= -RT lnK_eq_

Now, K_eq_ = e^-∆Go/RT^

R = universal gas constant (1.9872 cal K^-1^ mol^-1^), K_eq_ = equilibrium constant.

**
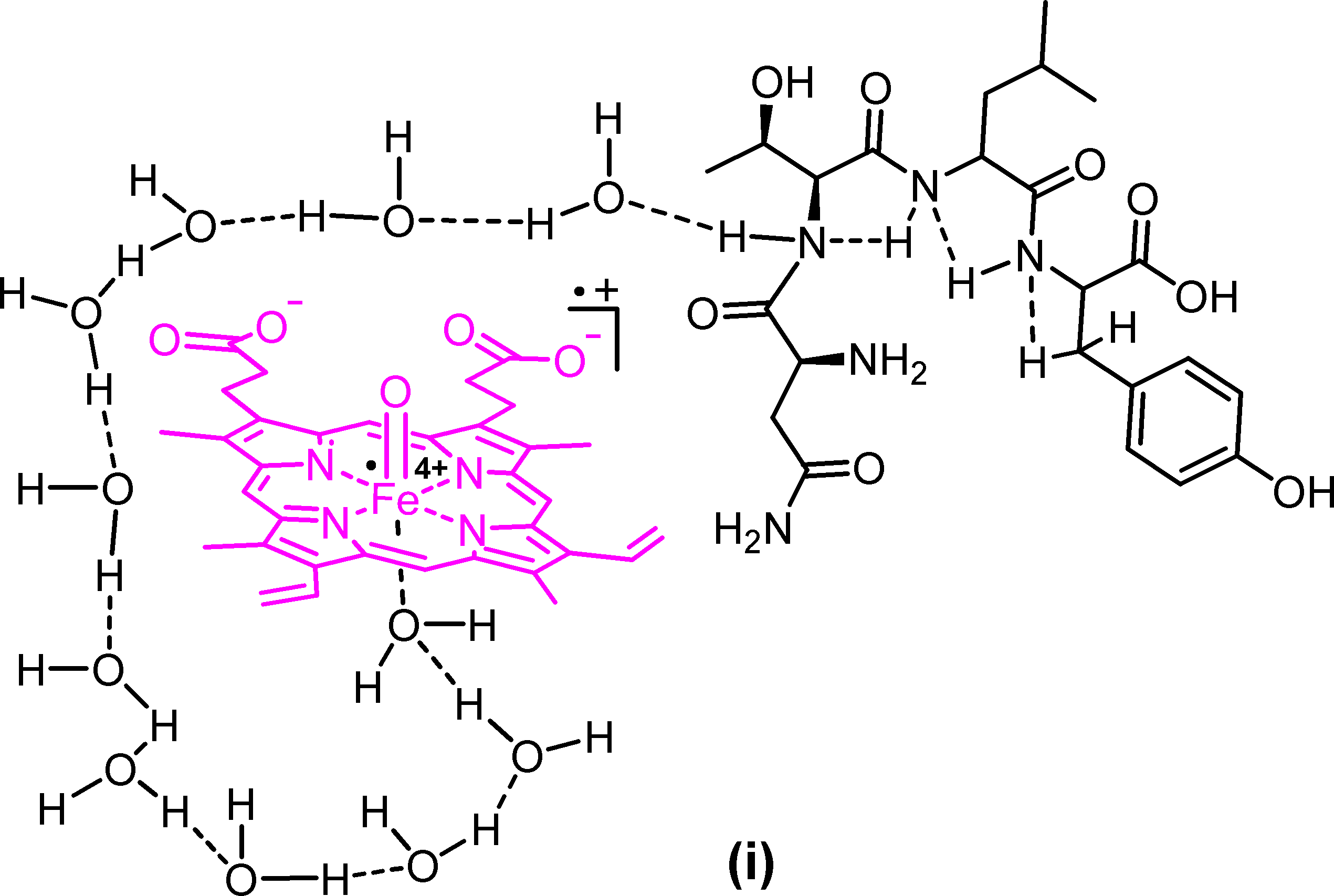

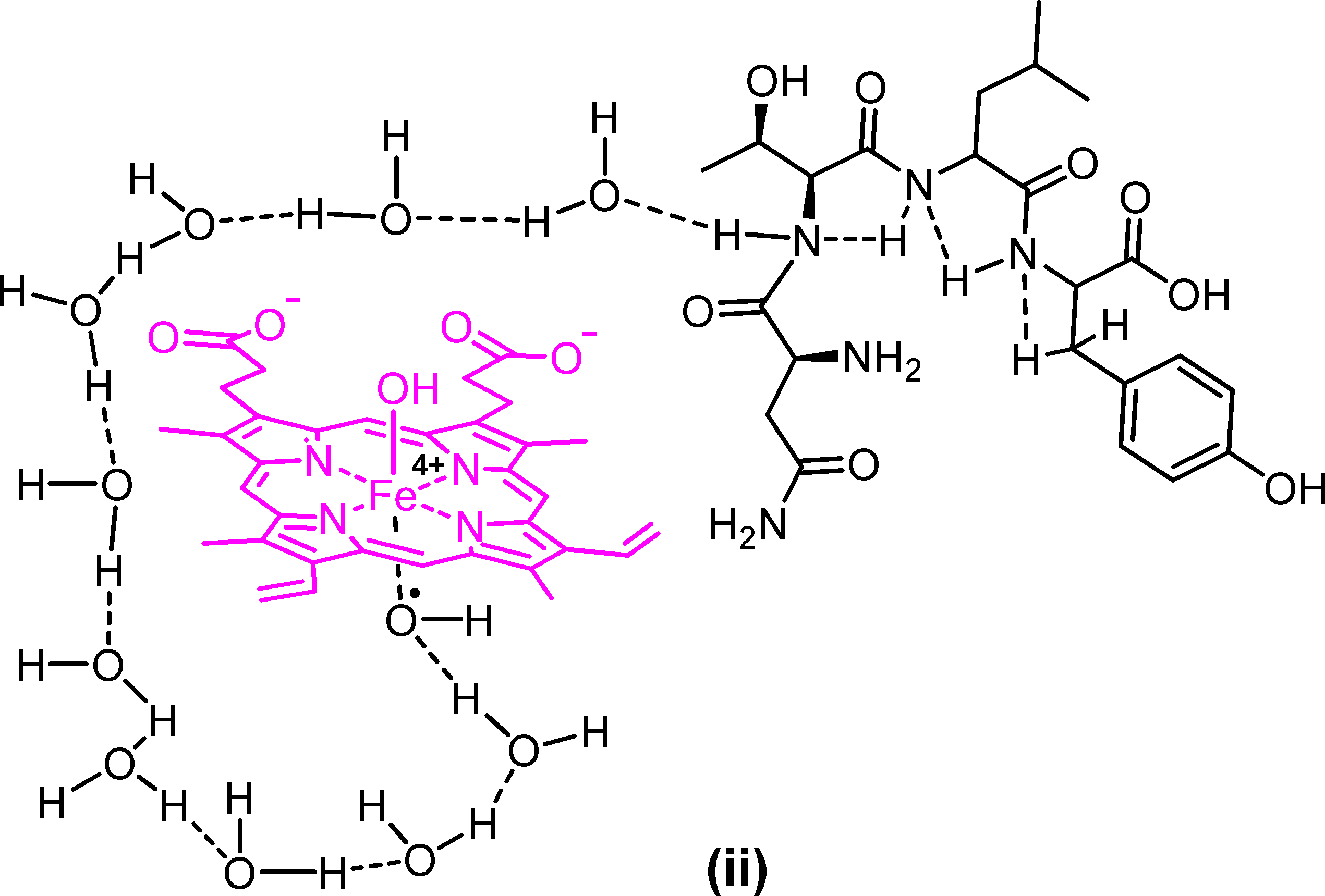
**

**
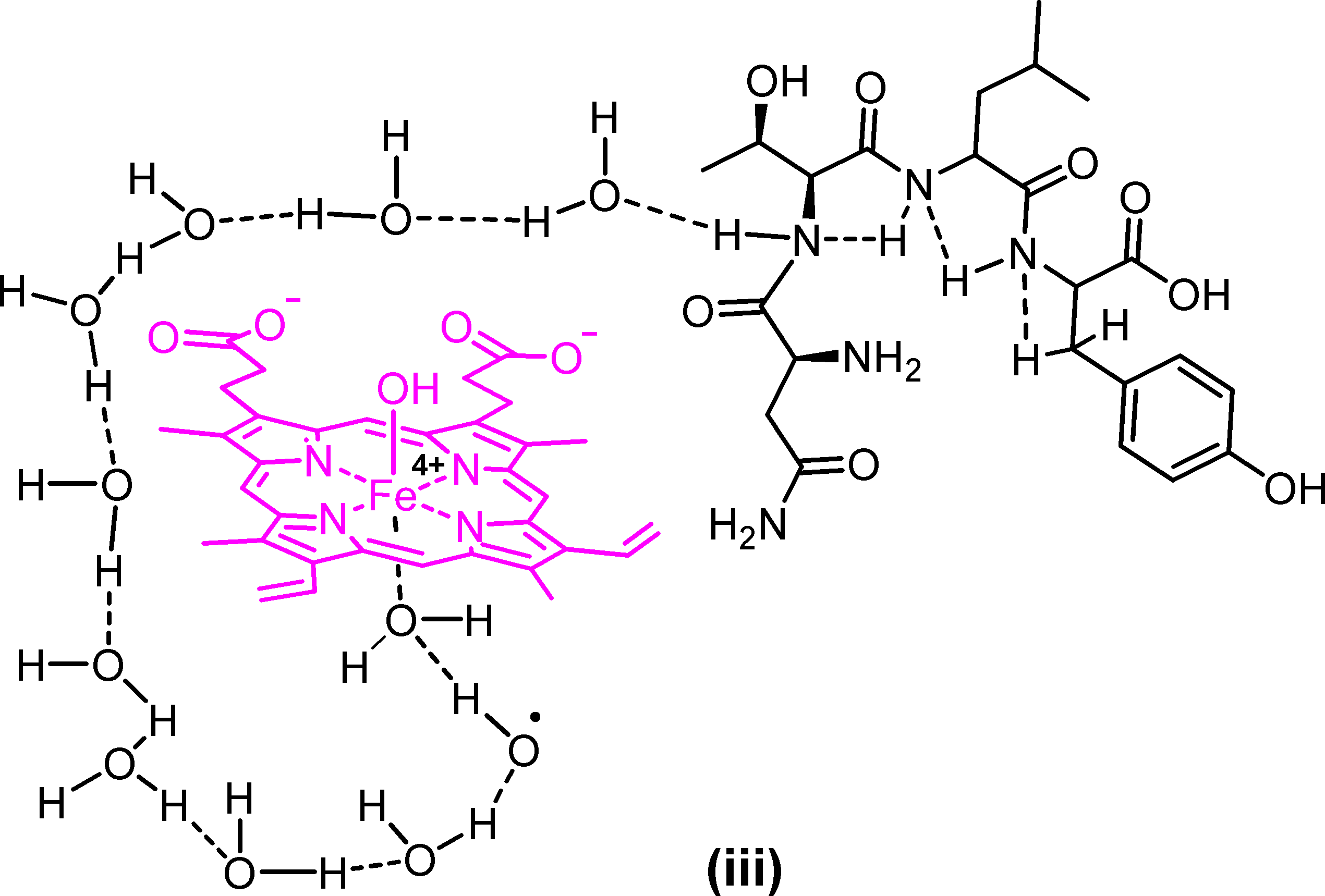

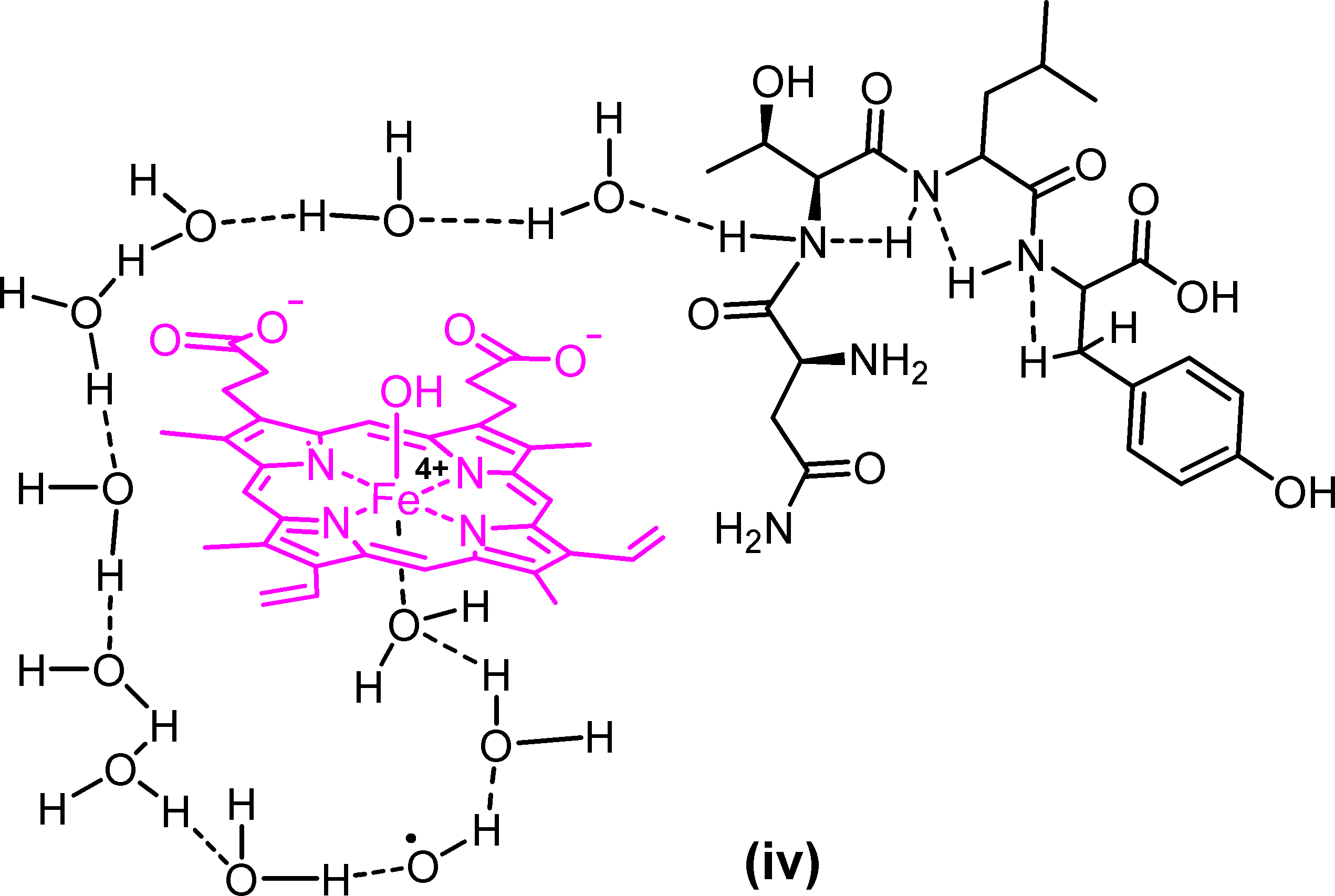
**

**
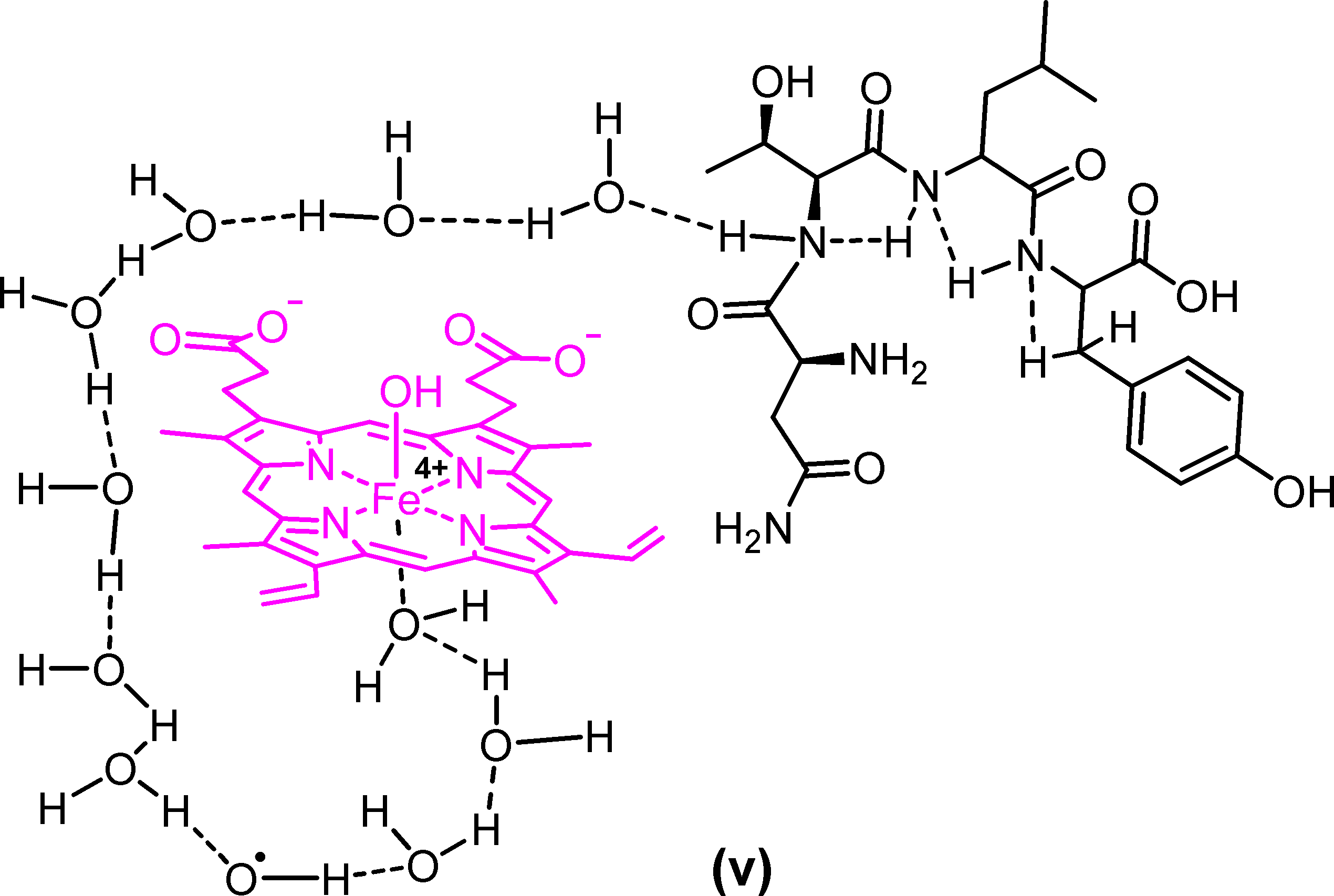

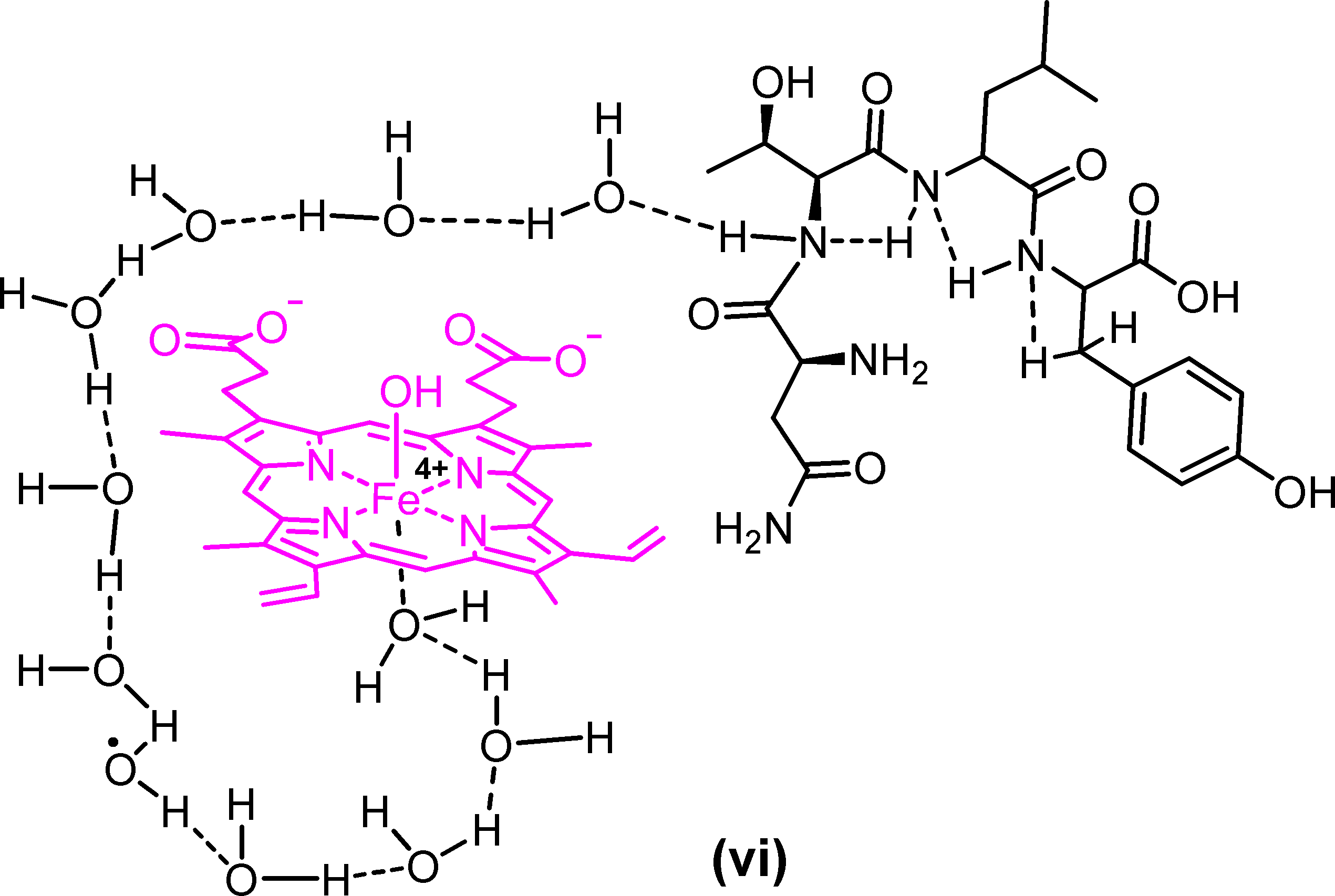
**

**
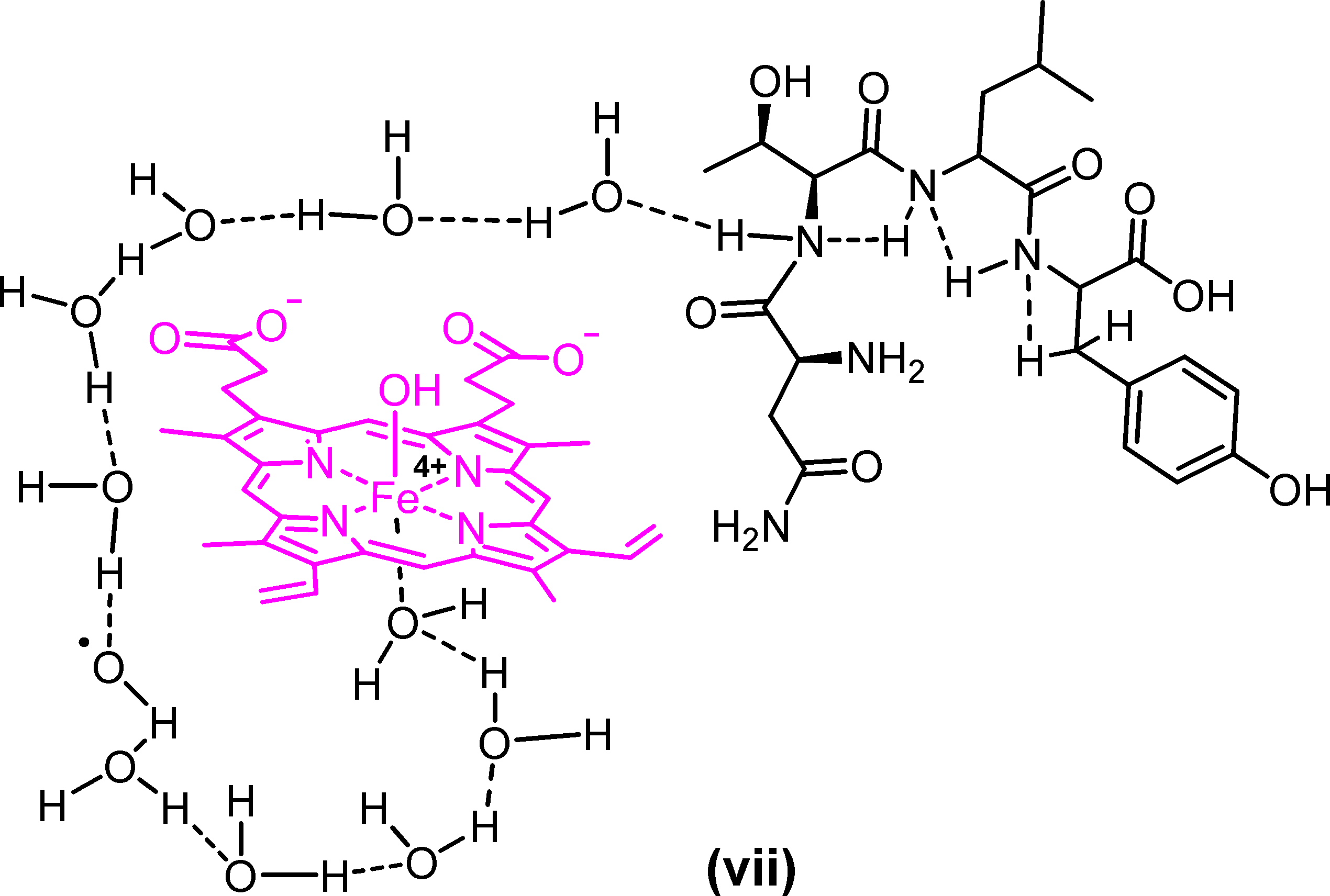

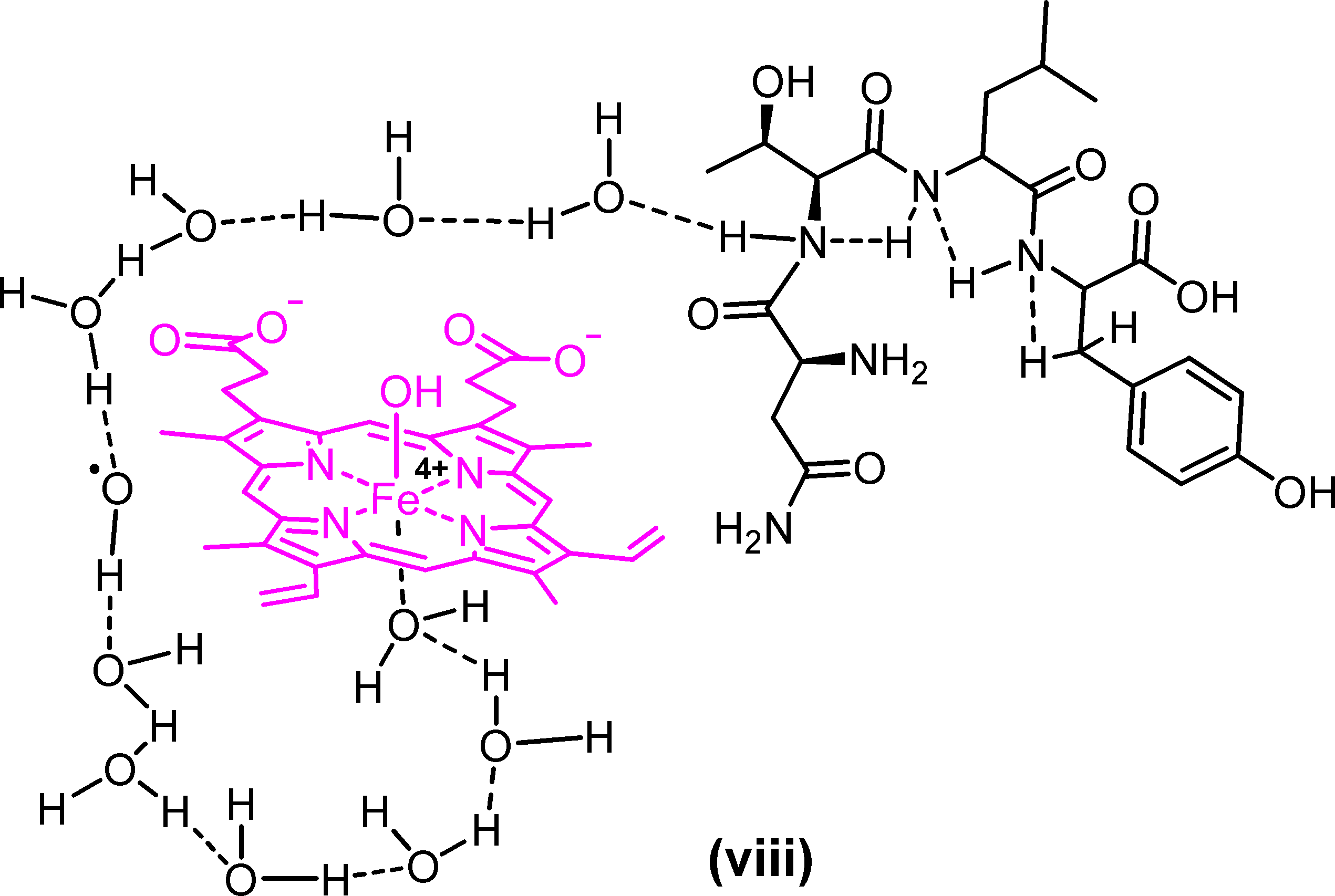
**

**
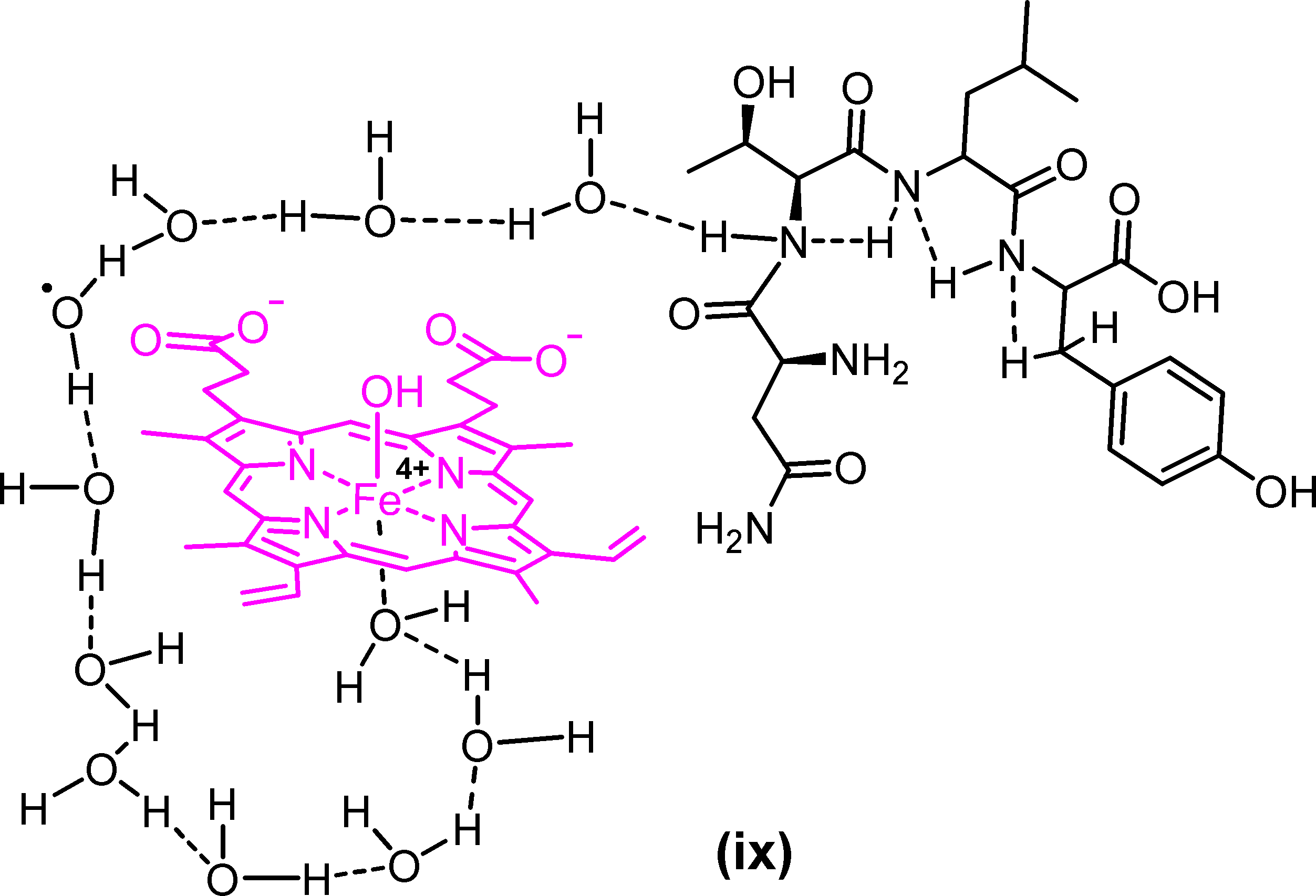

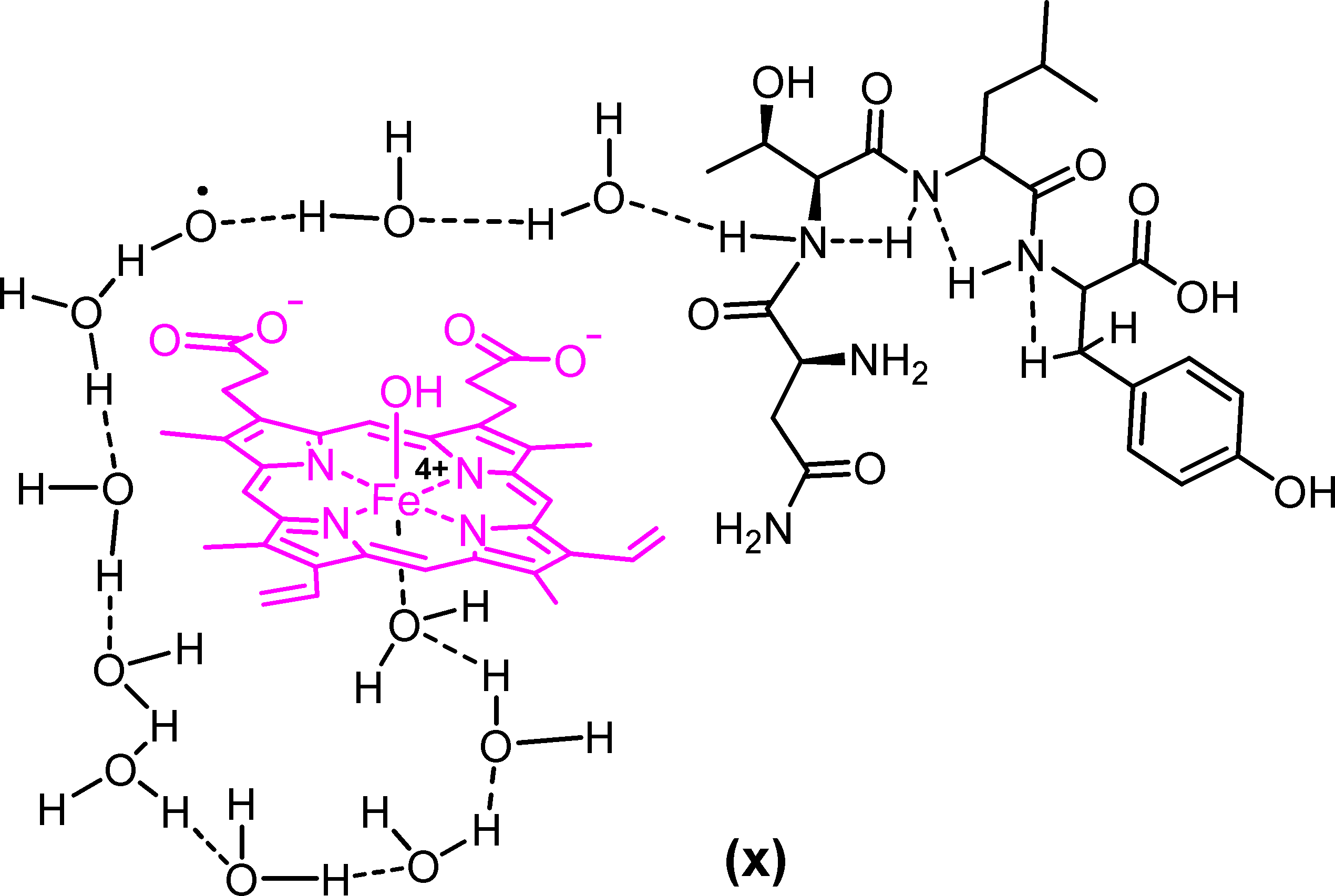
**

**Figure S7**. The radical carrying systems for channel ‘a’ built for calculating the single point energy.

**Figure S7 contd..**

**
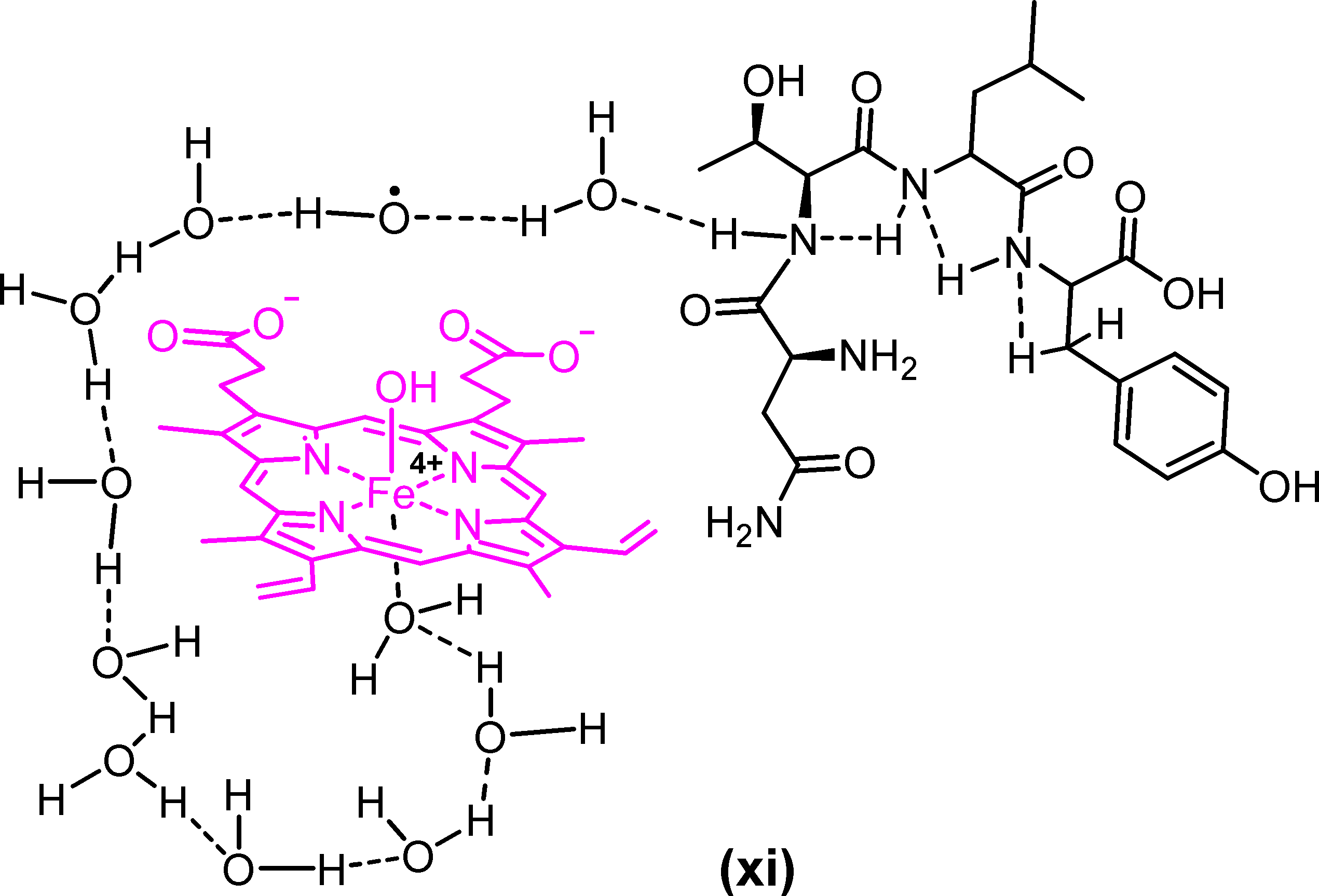

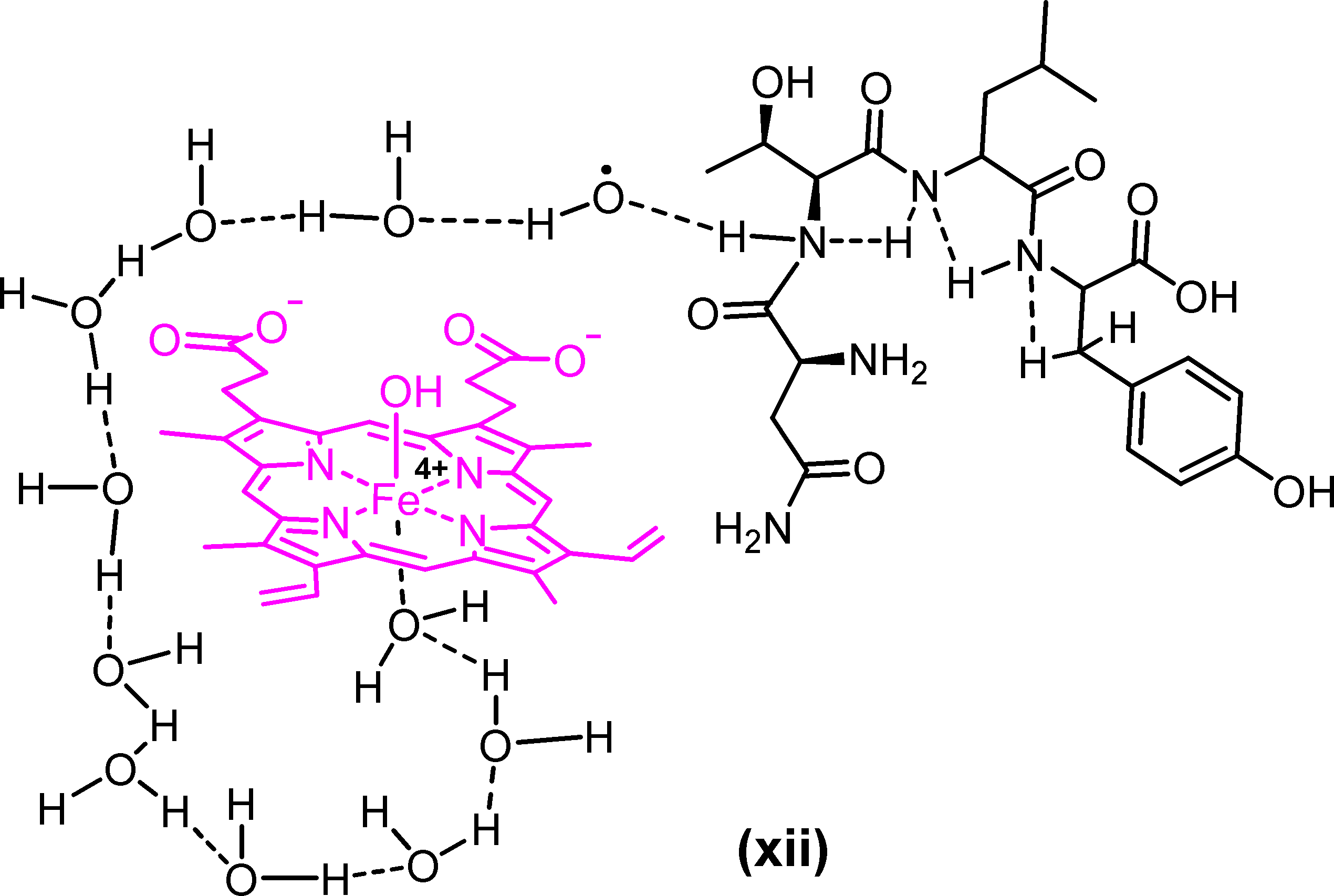
**

**
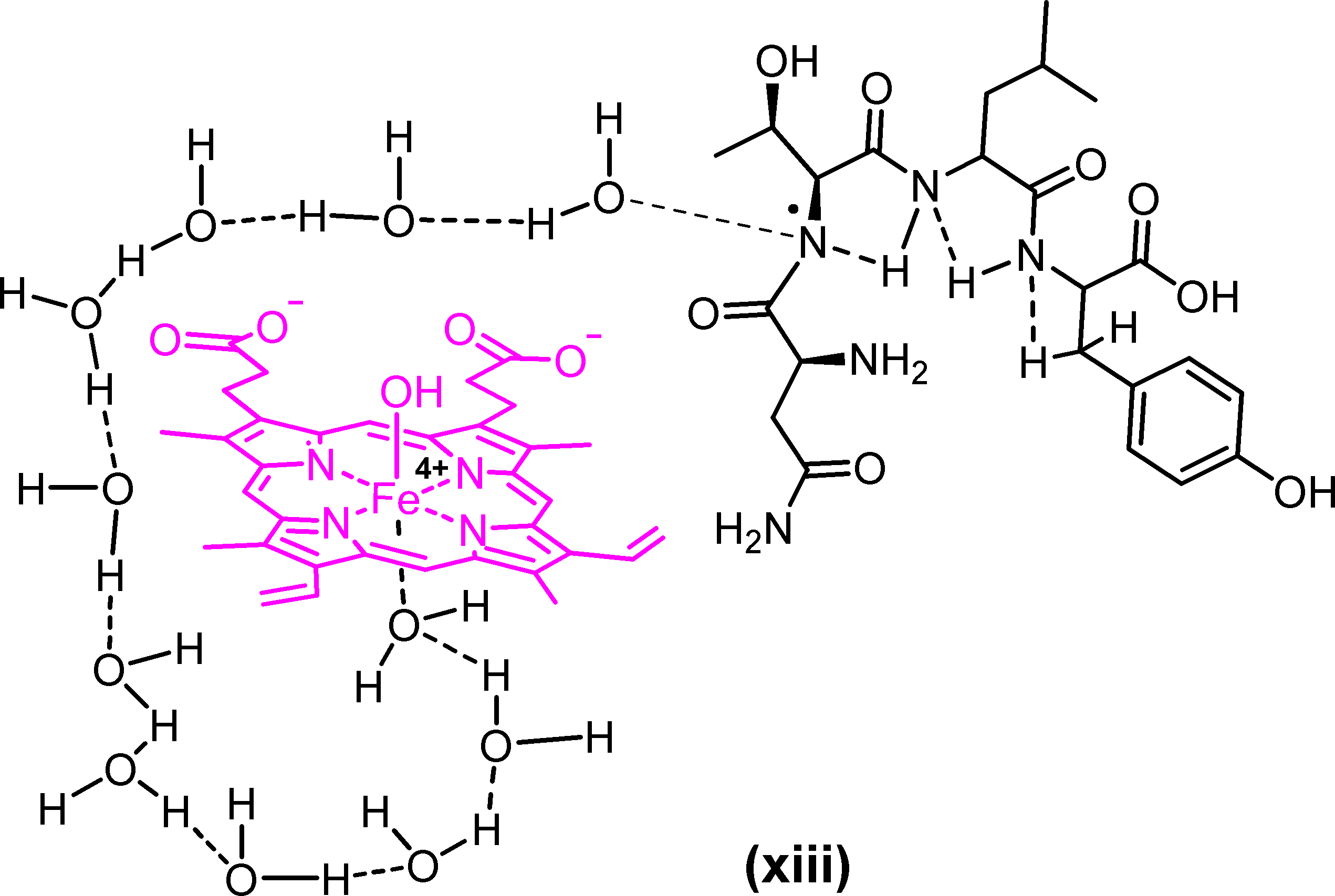

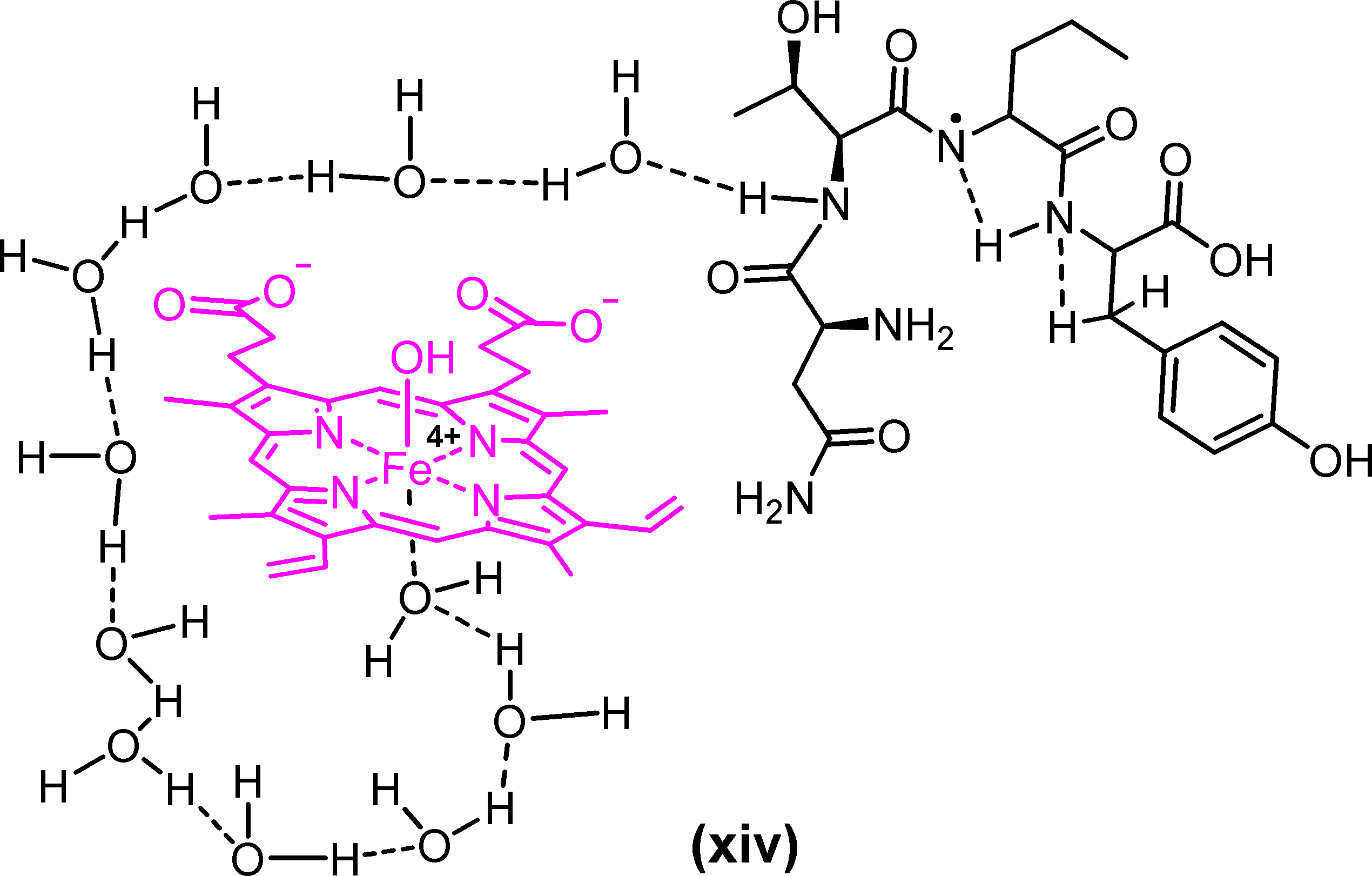
**

**
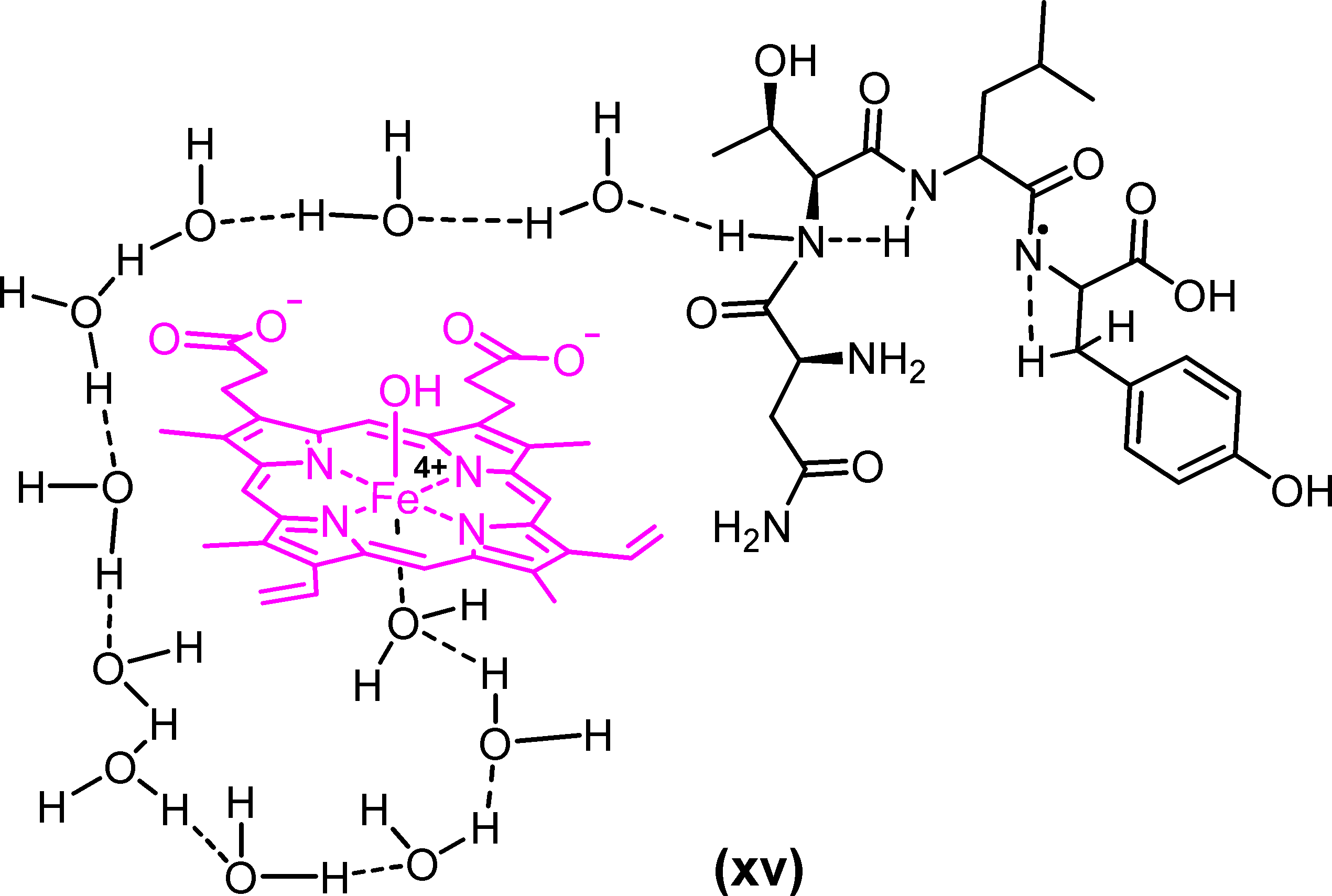

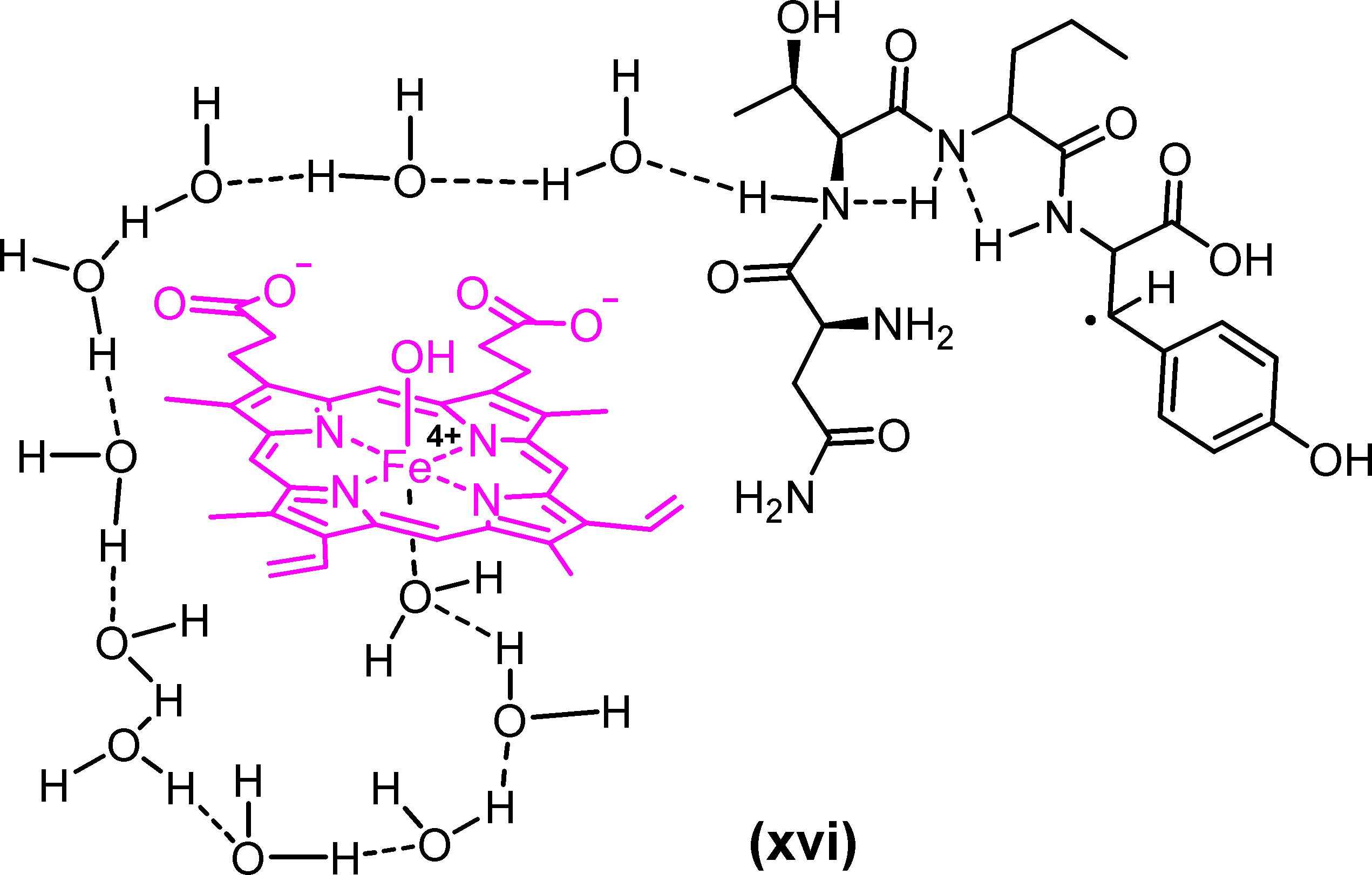
**

**
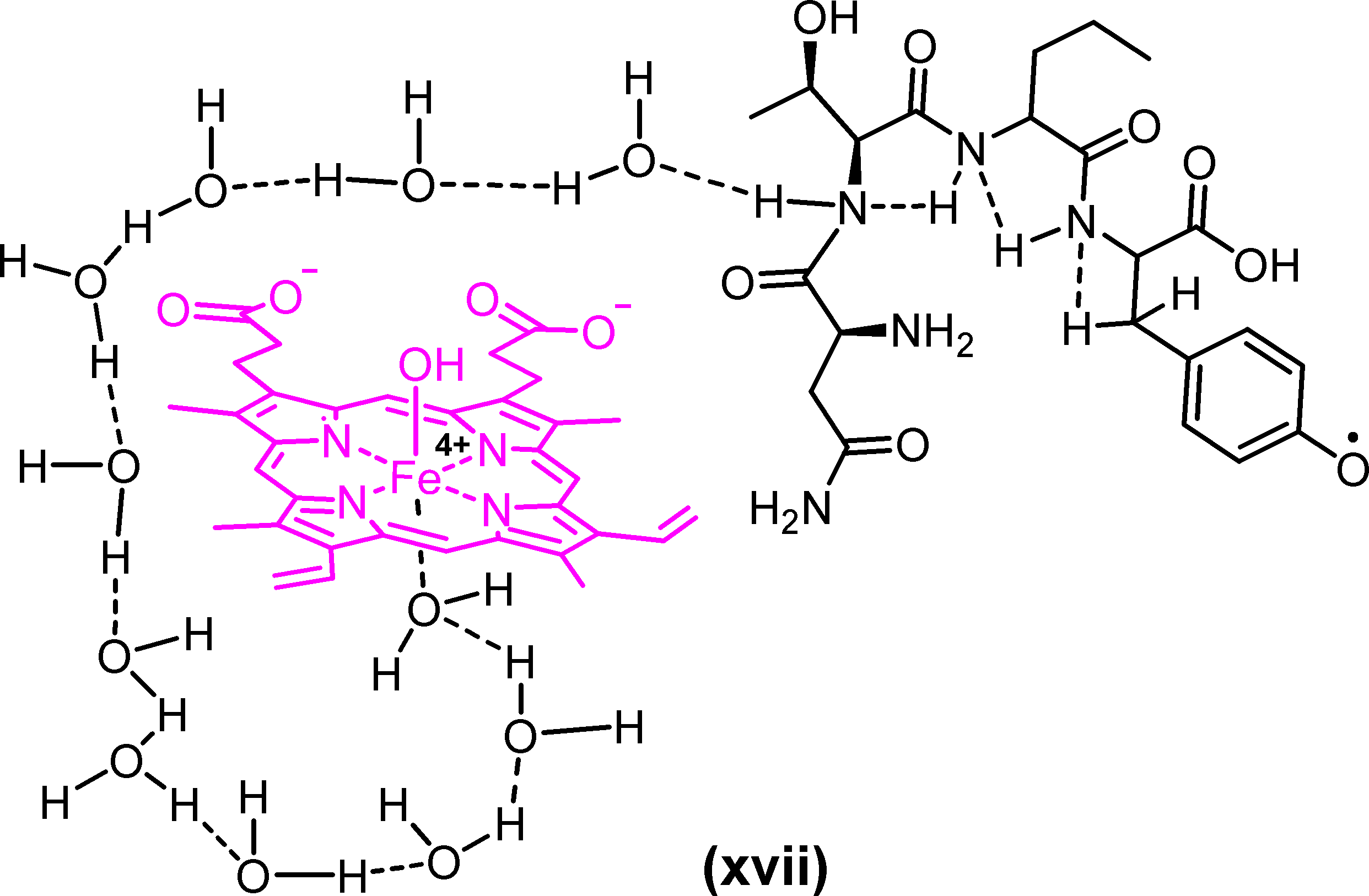
**

**
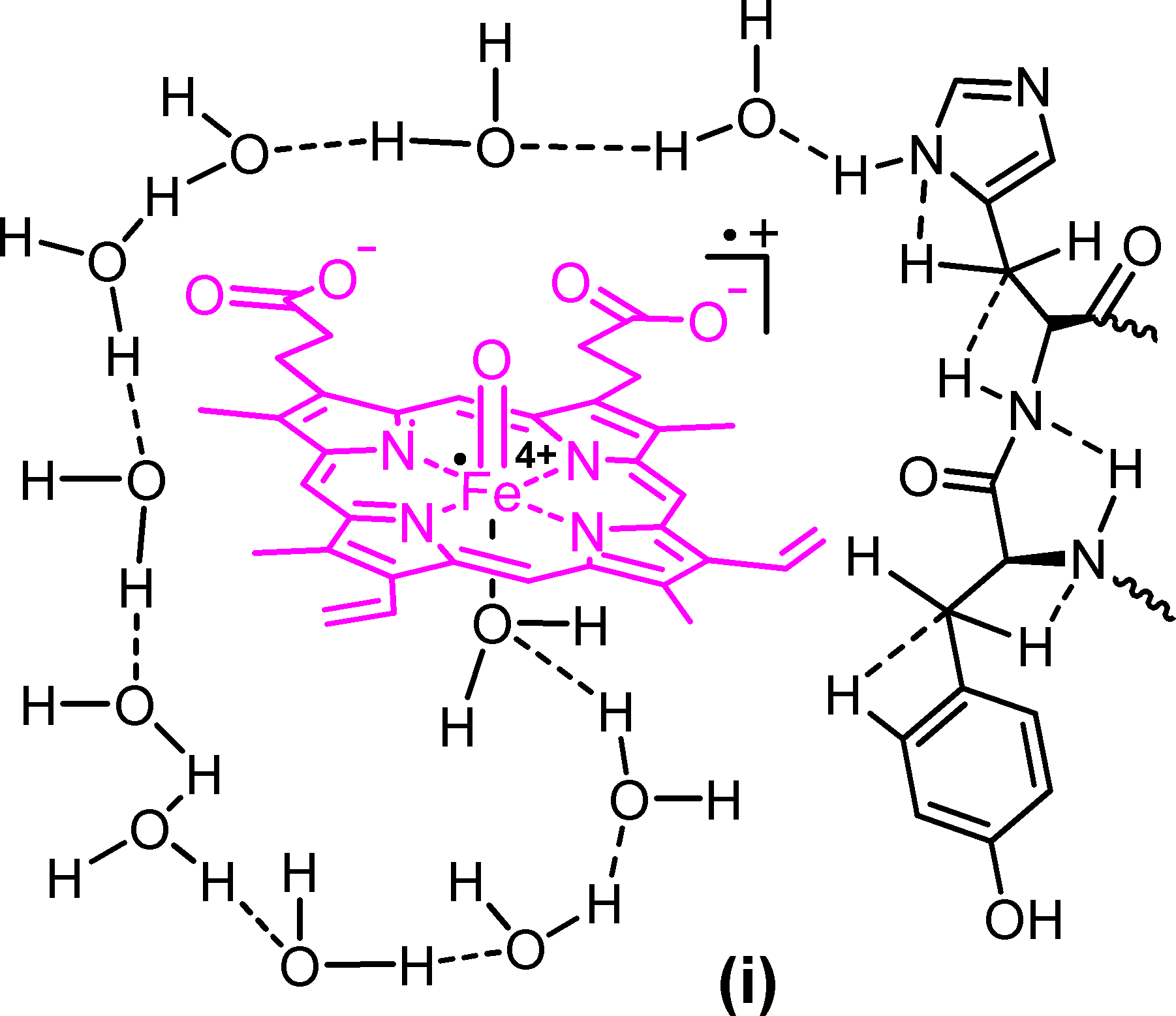

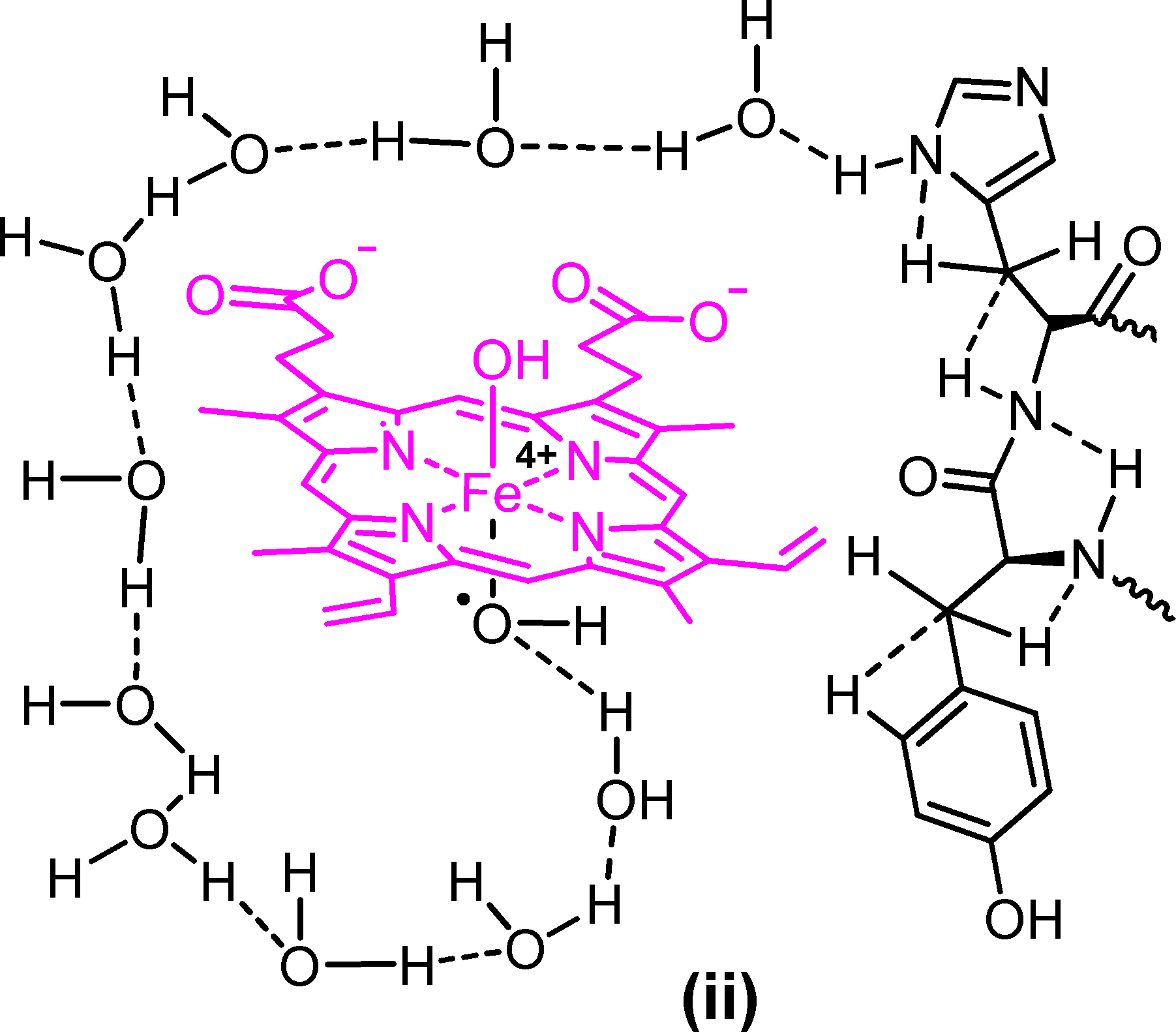
**

**
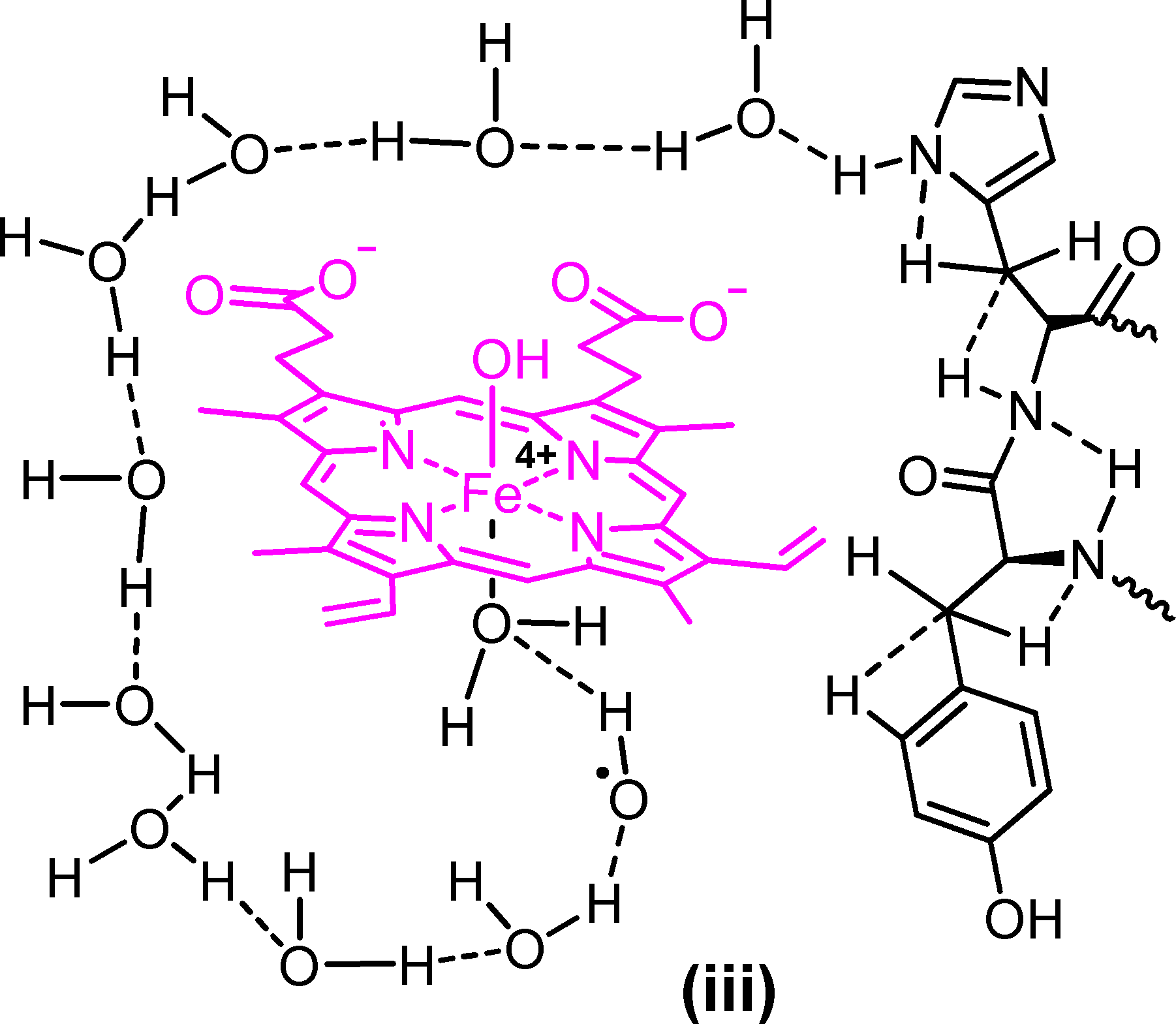

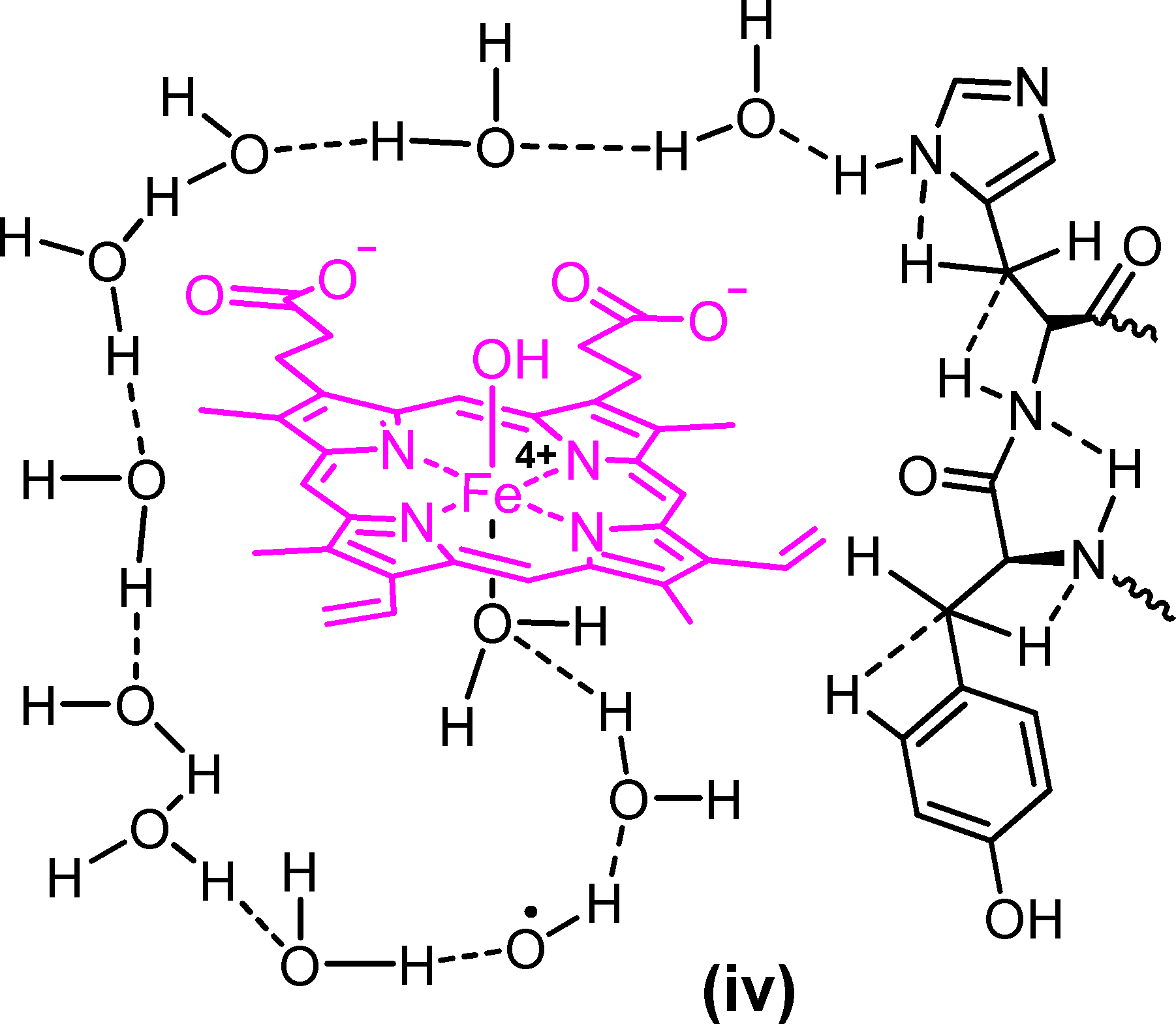
**

**
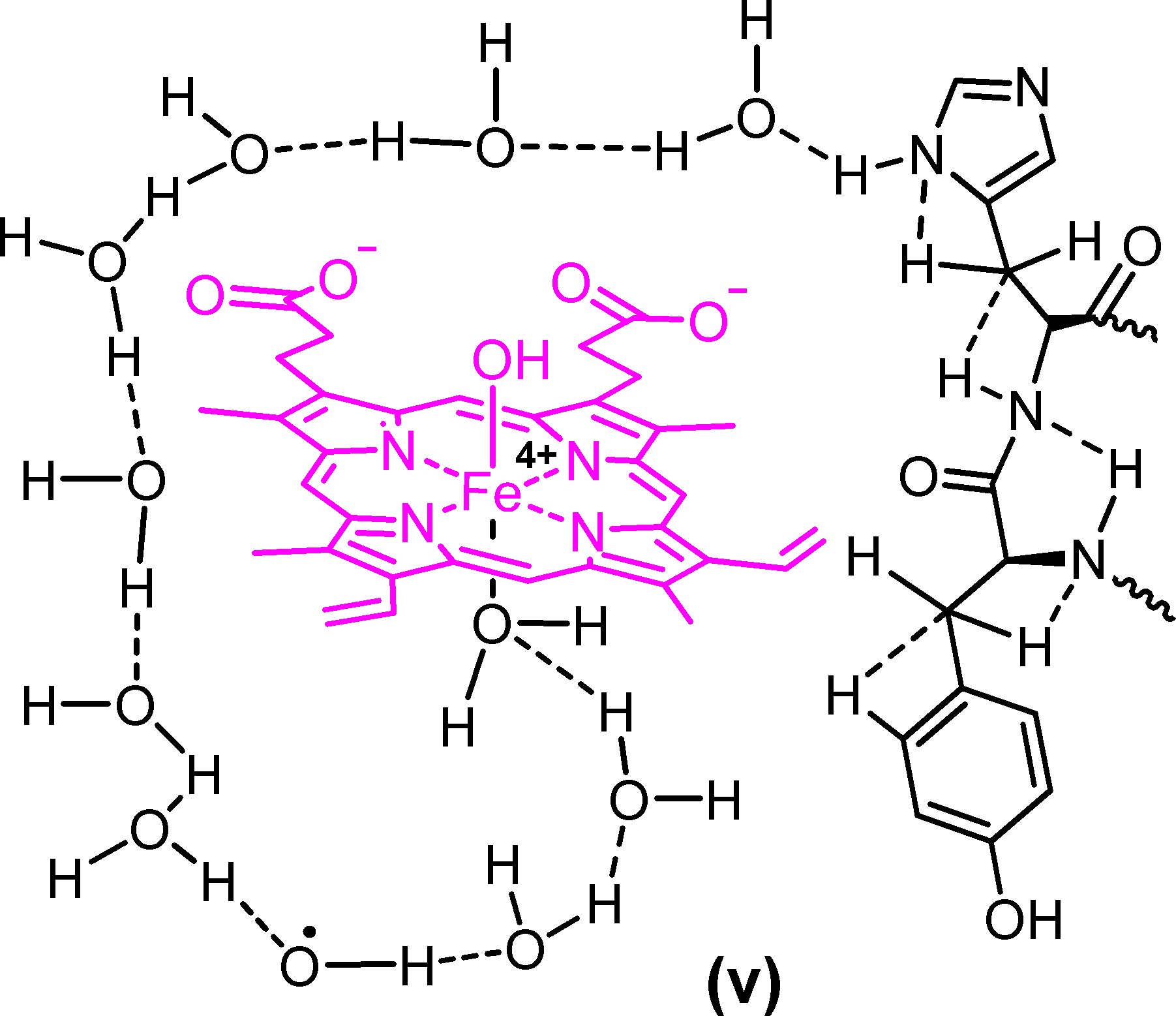

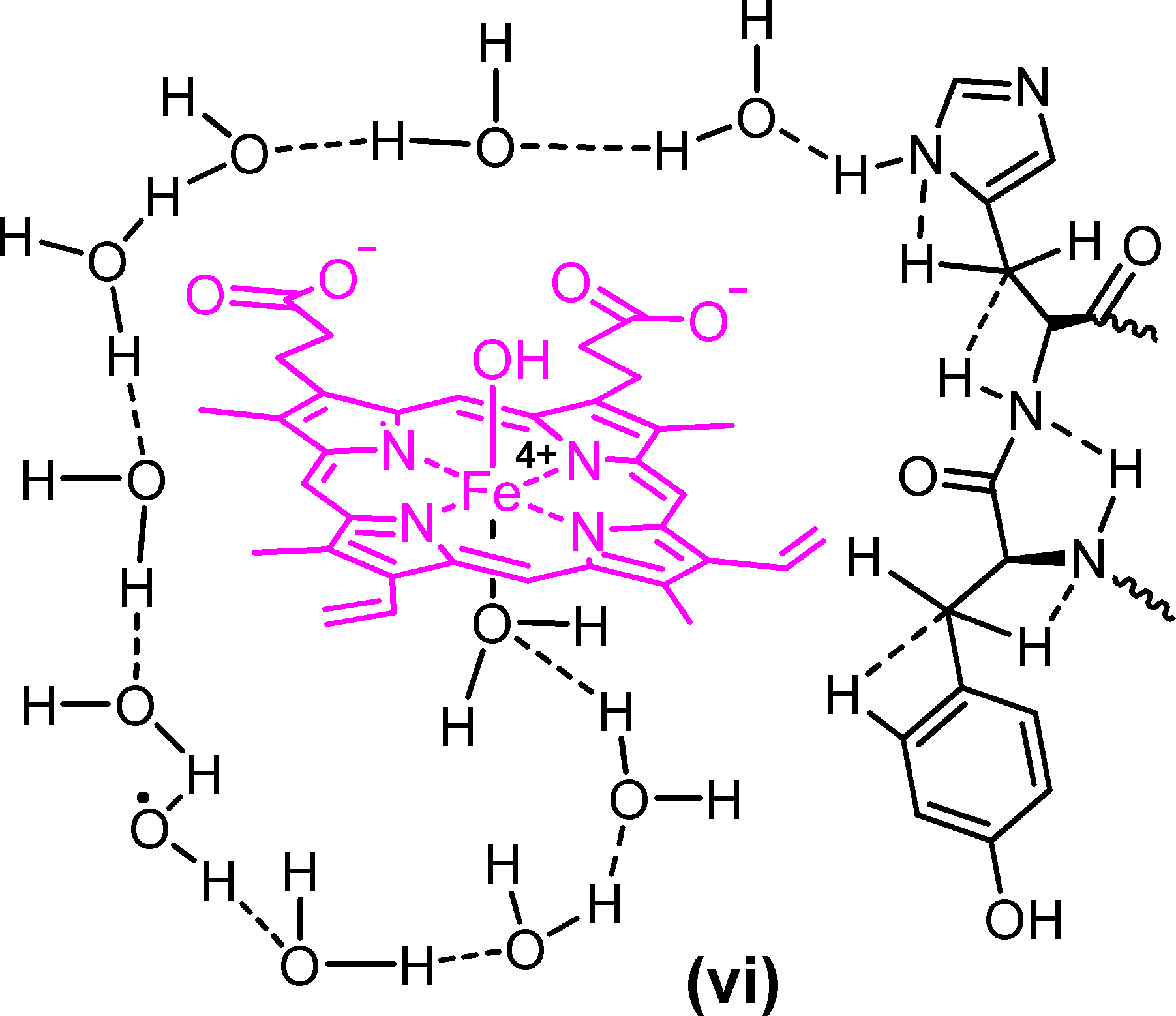
**

**Figure S8**. The radical carrying systems for channel ‘b’ built for calculating the single point energy.

**Figure S8 contd..**

**
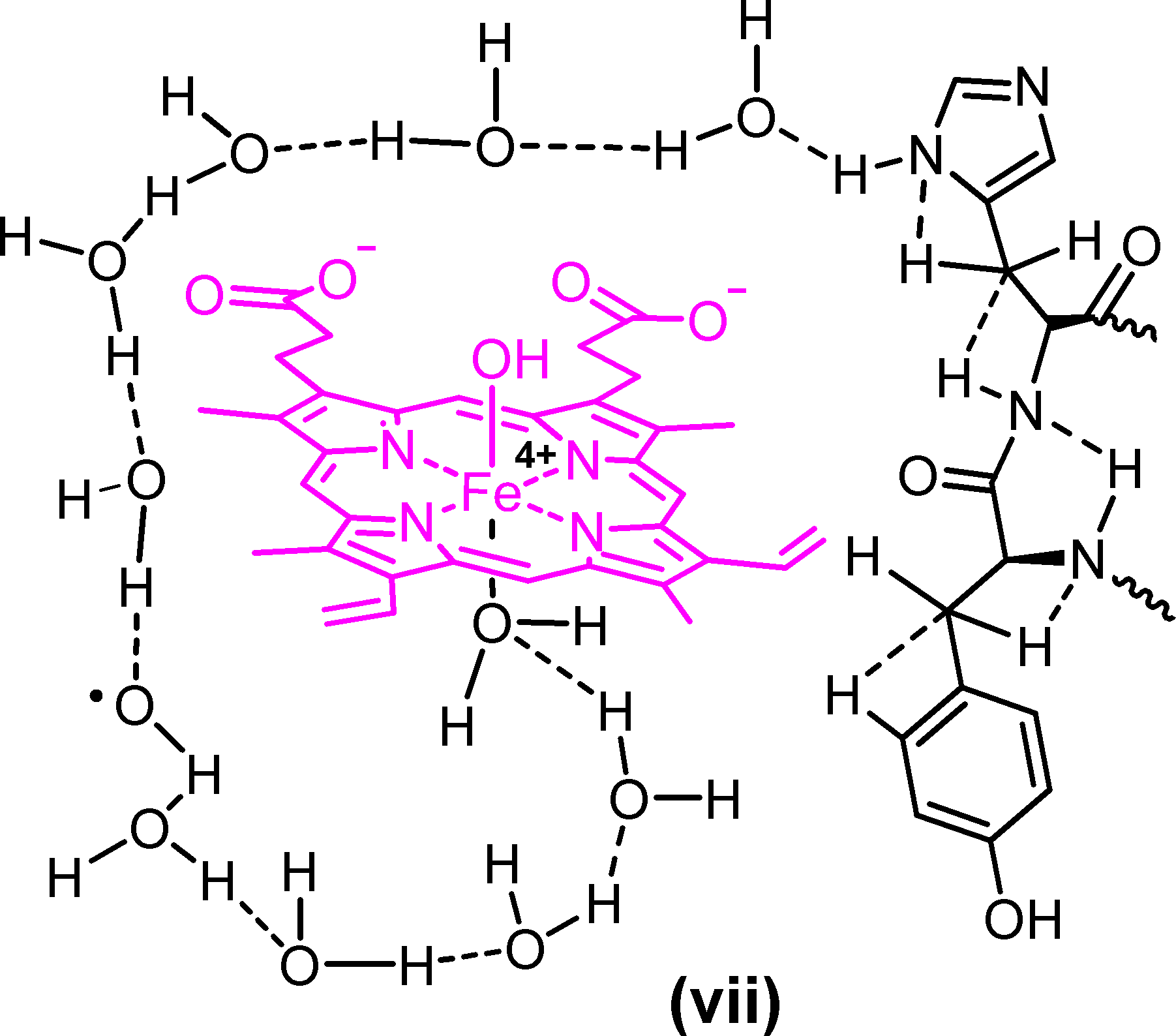

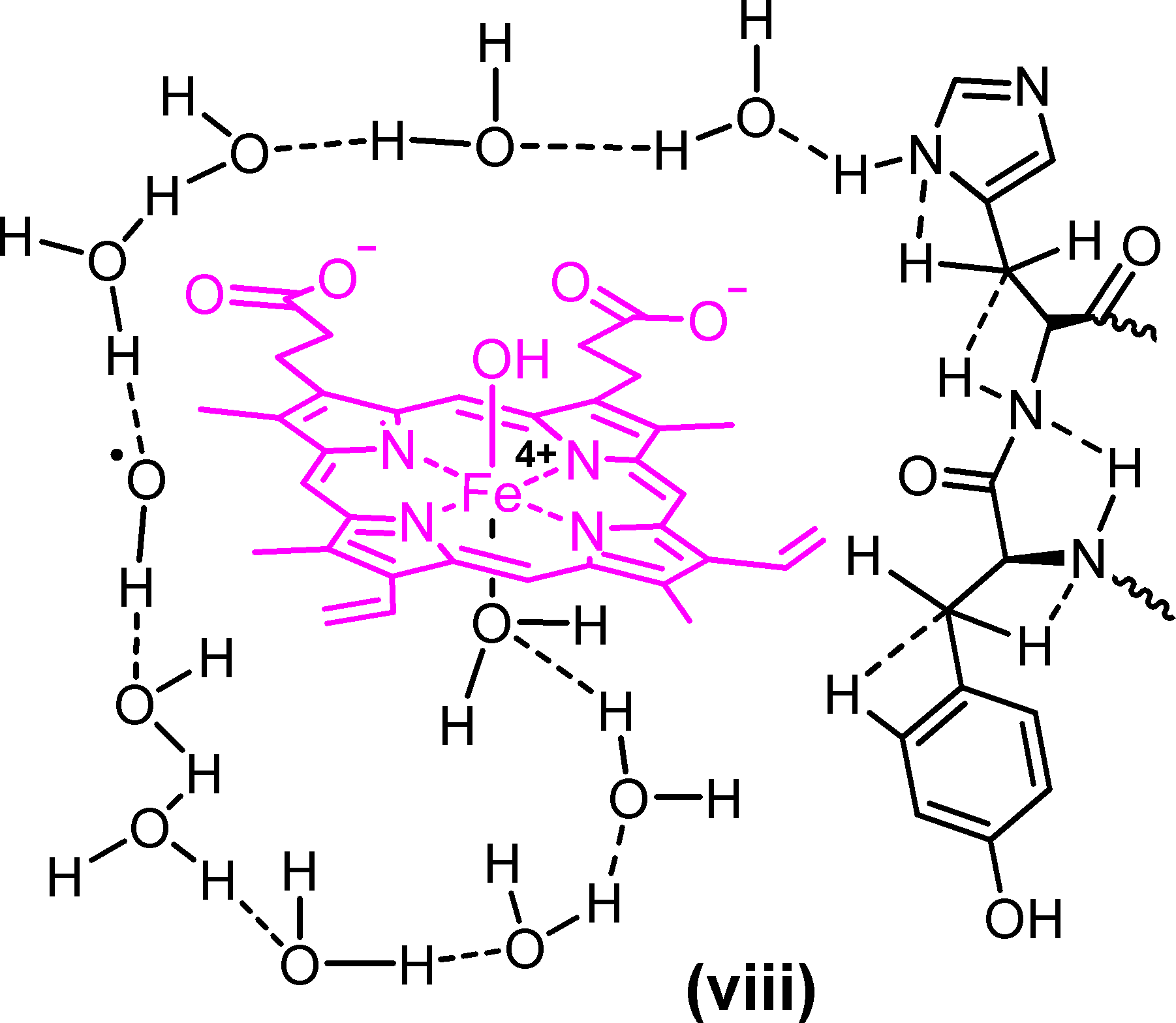
**

**
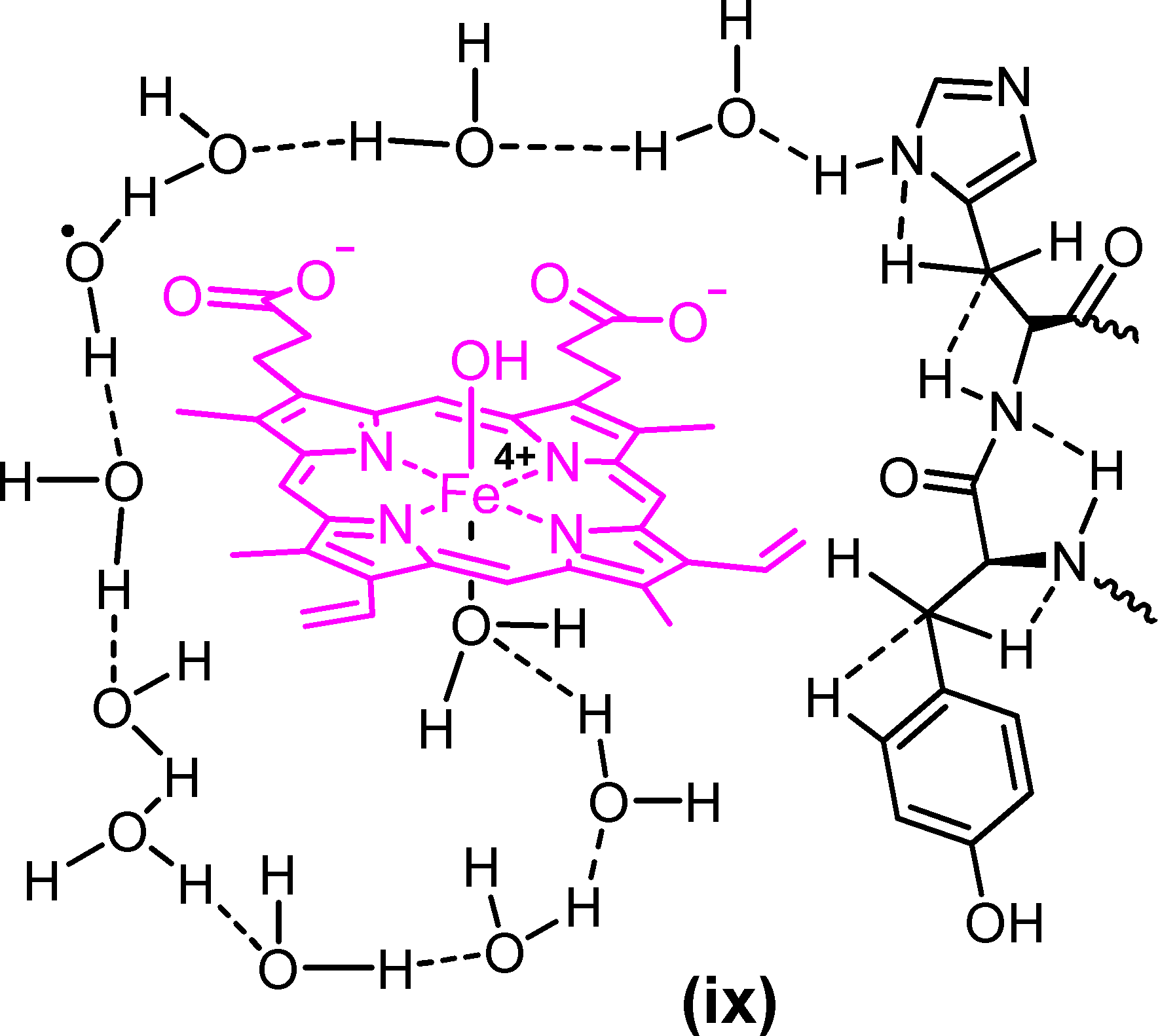

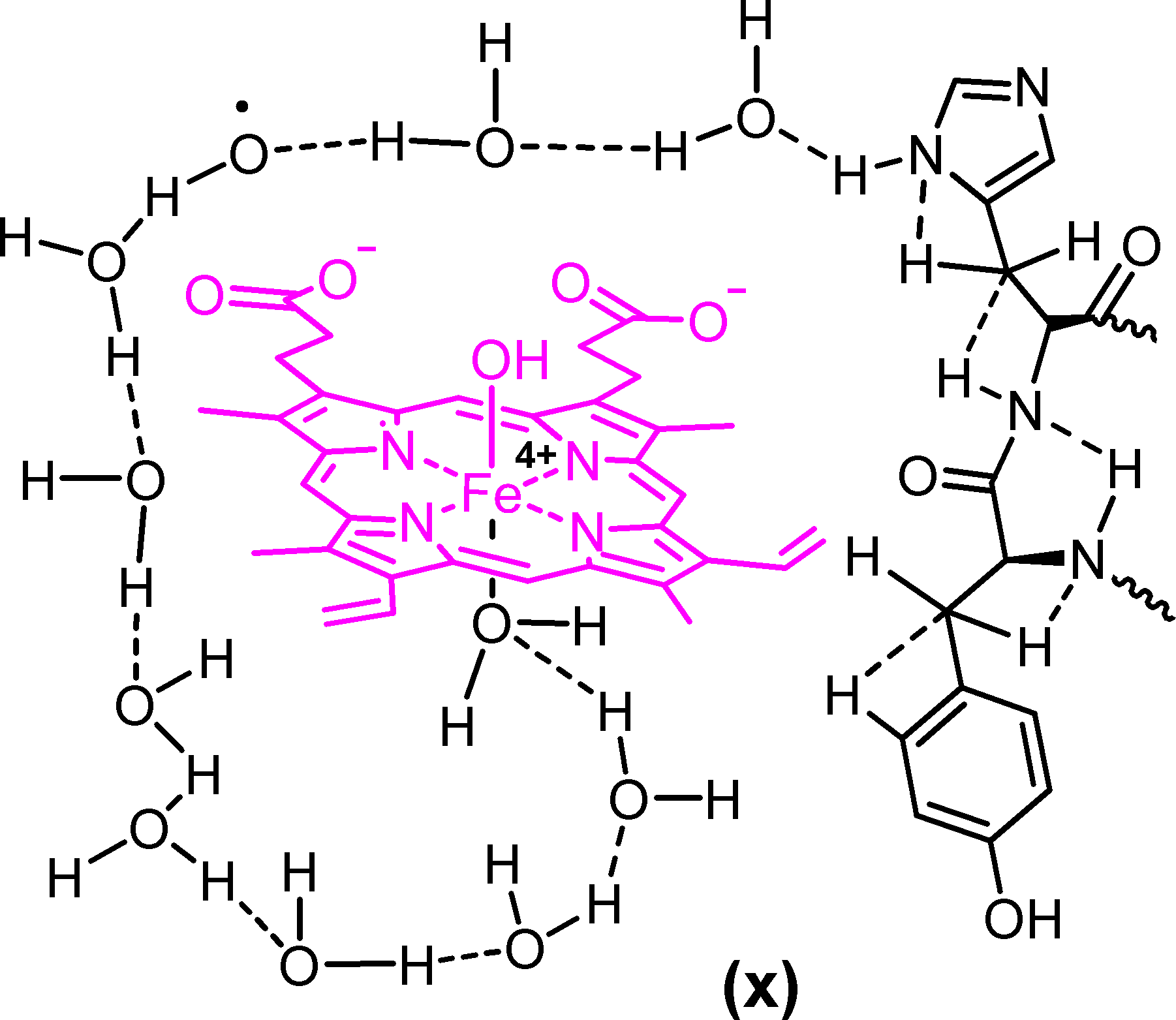
**

**
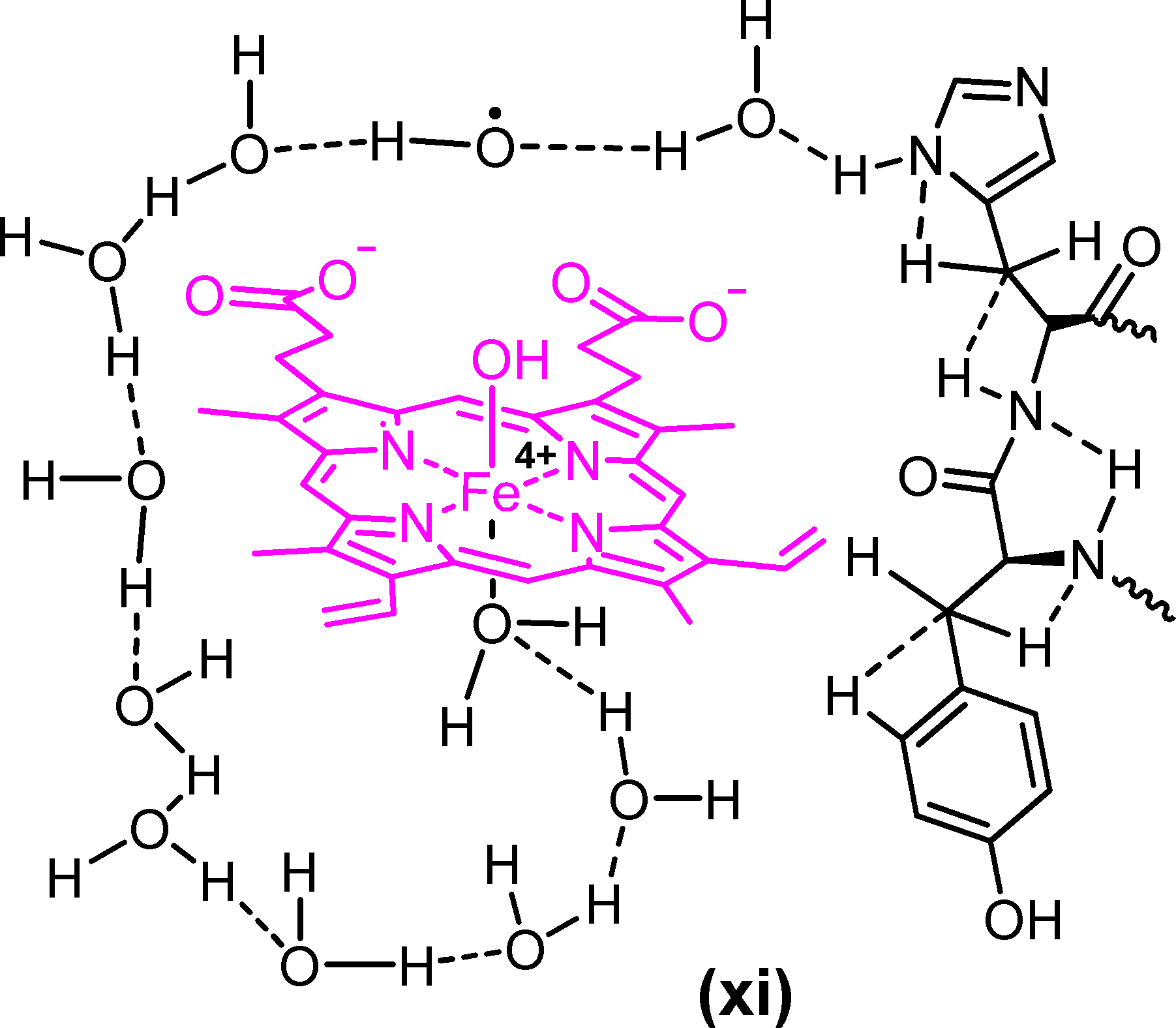

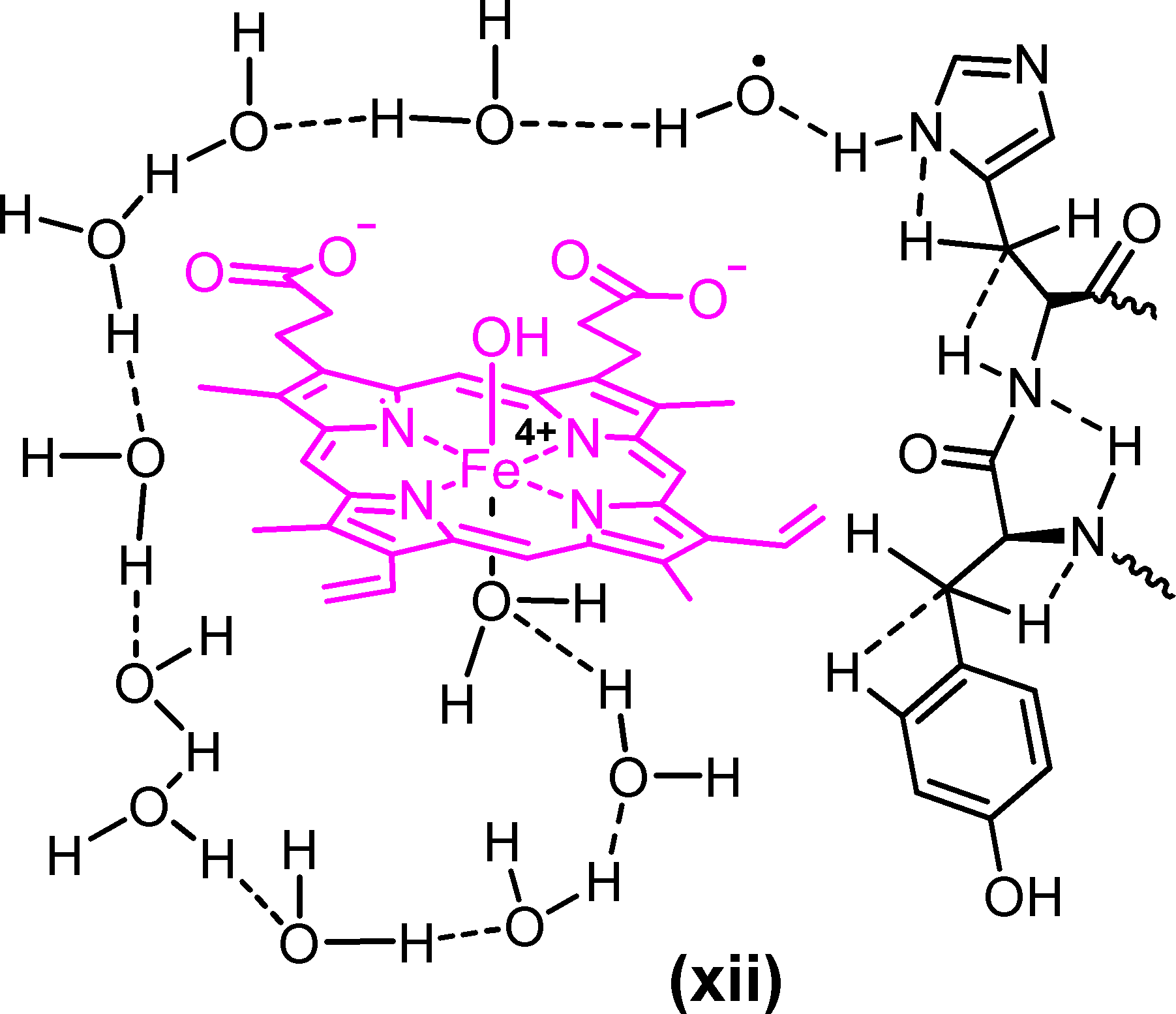
**

**Figure S8 contd..**

**
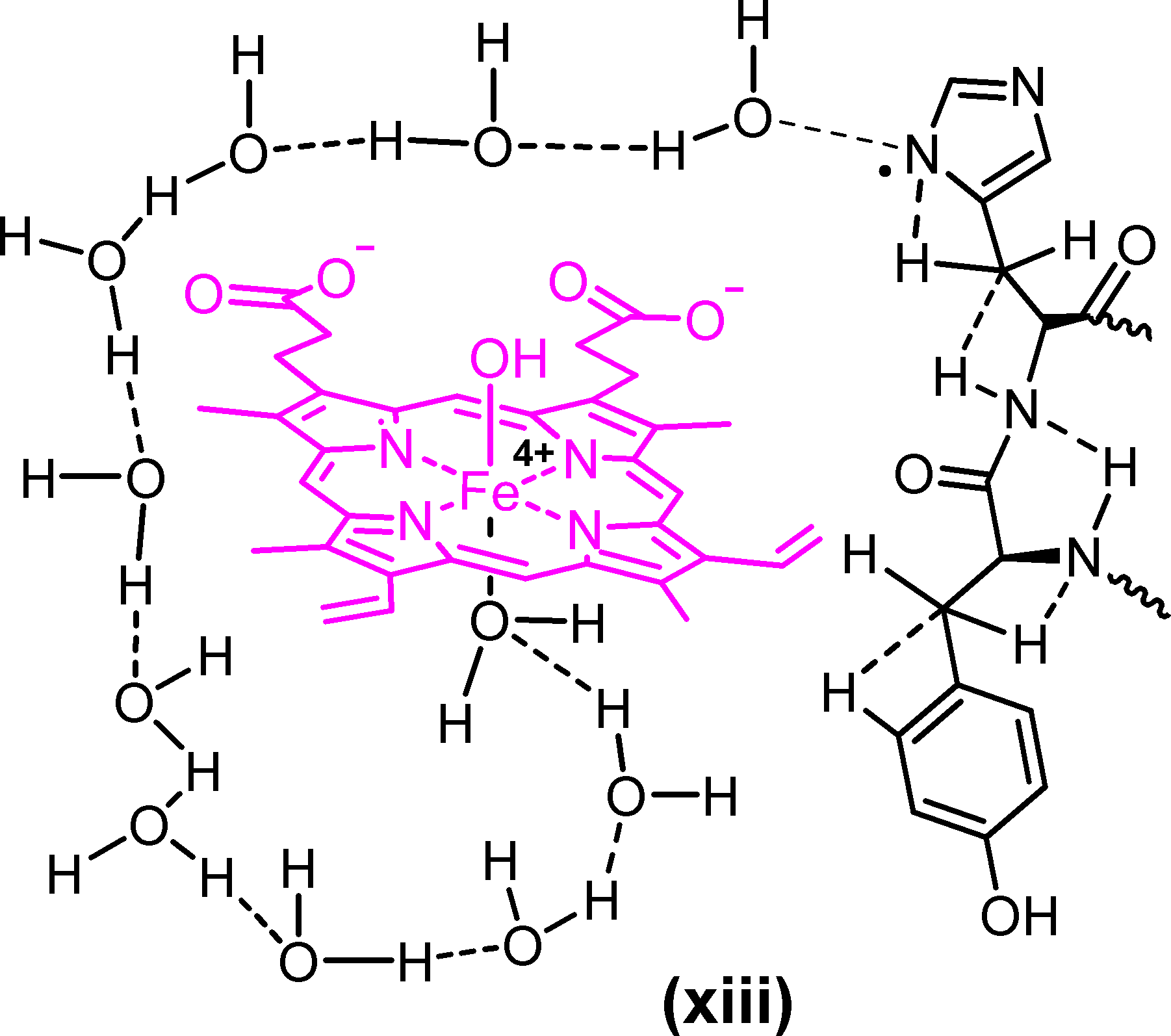

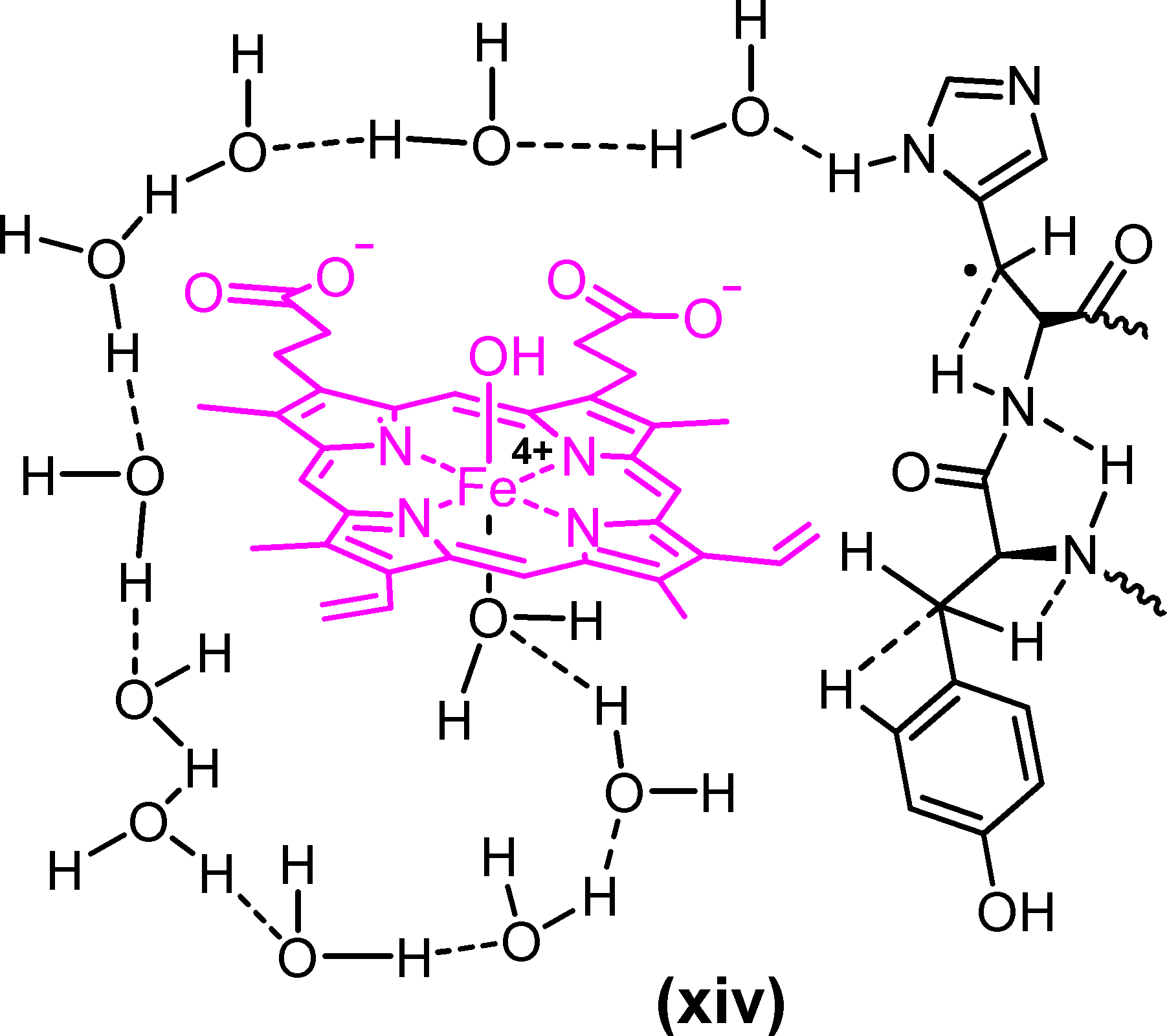
**

**
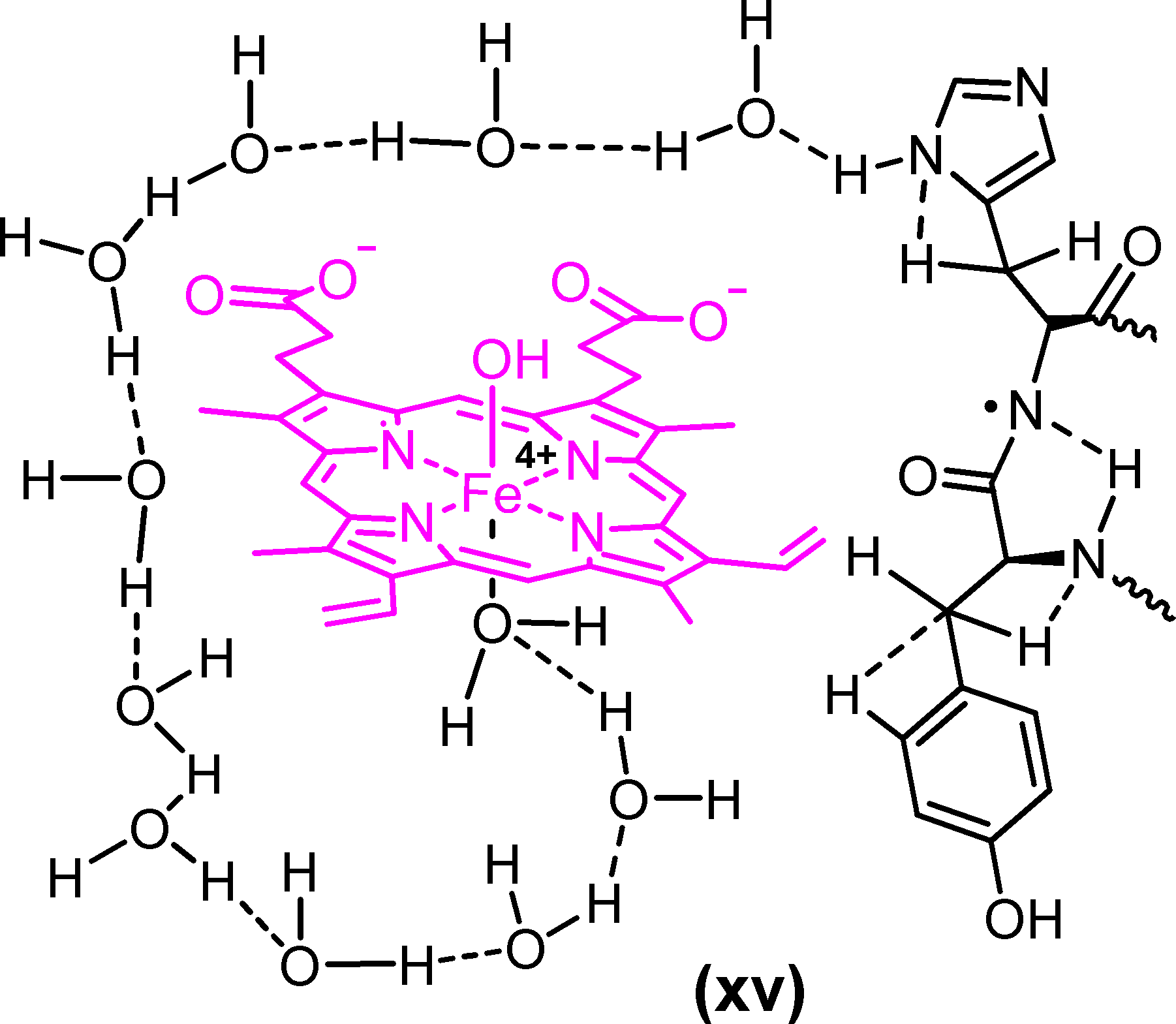

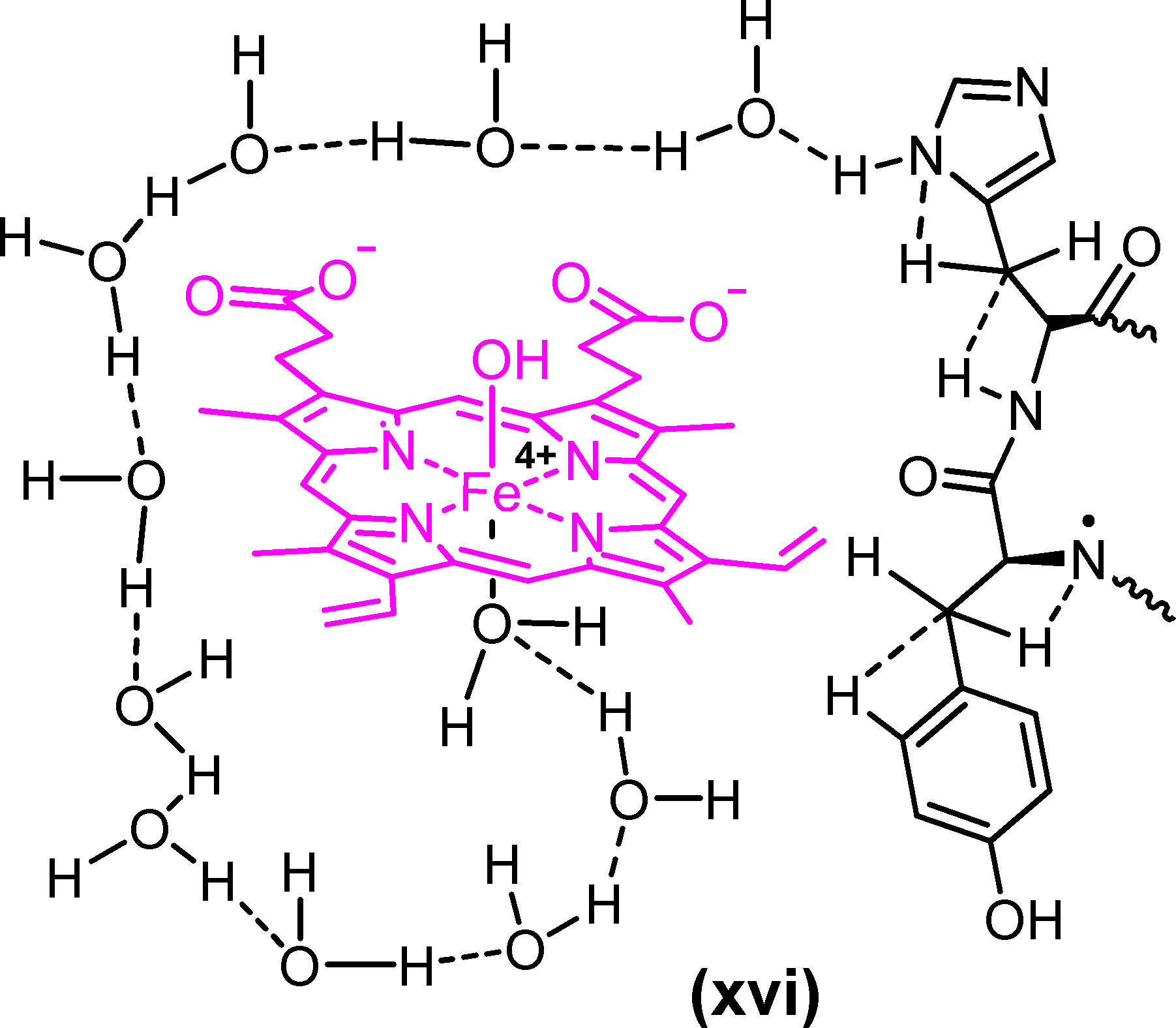
**

**
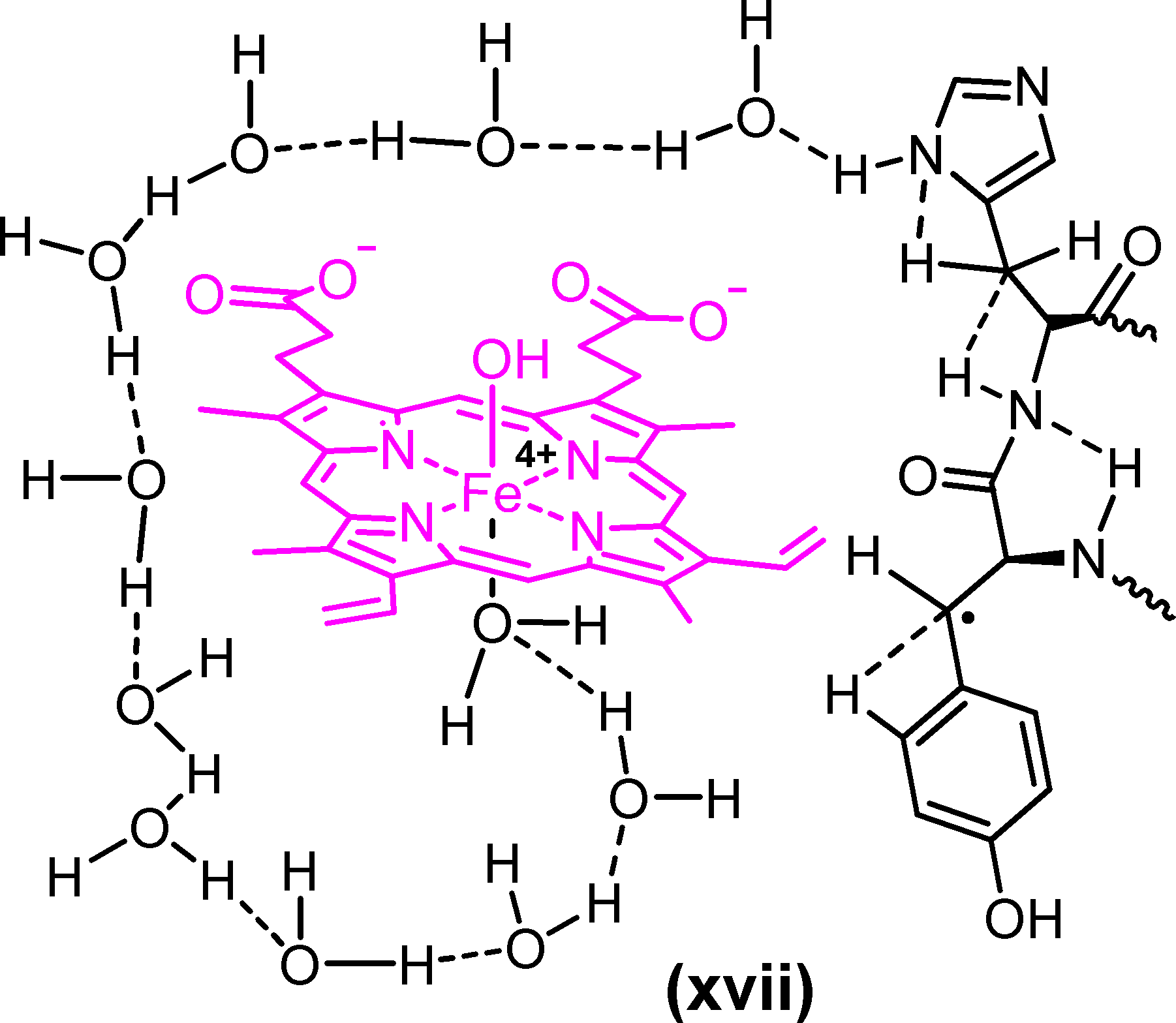

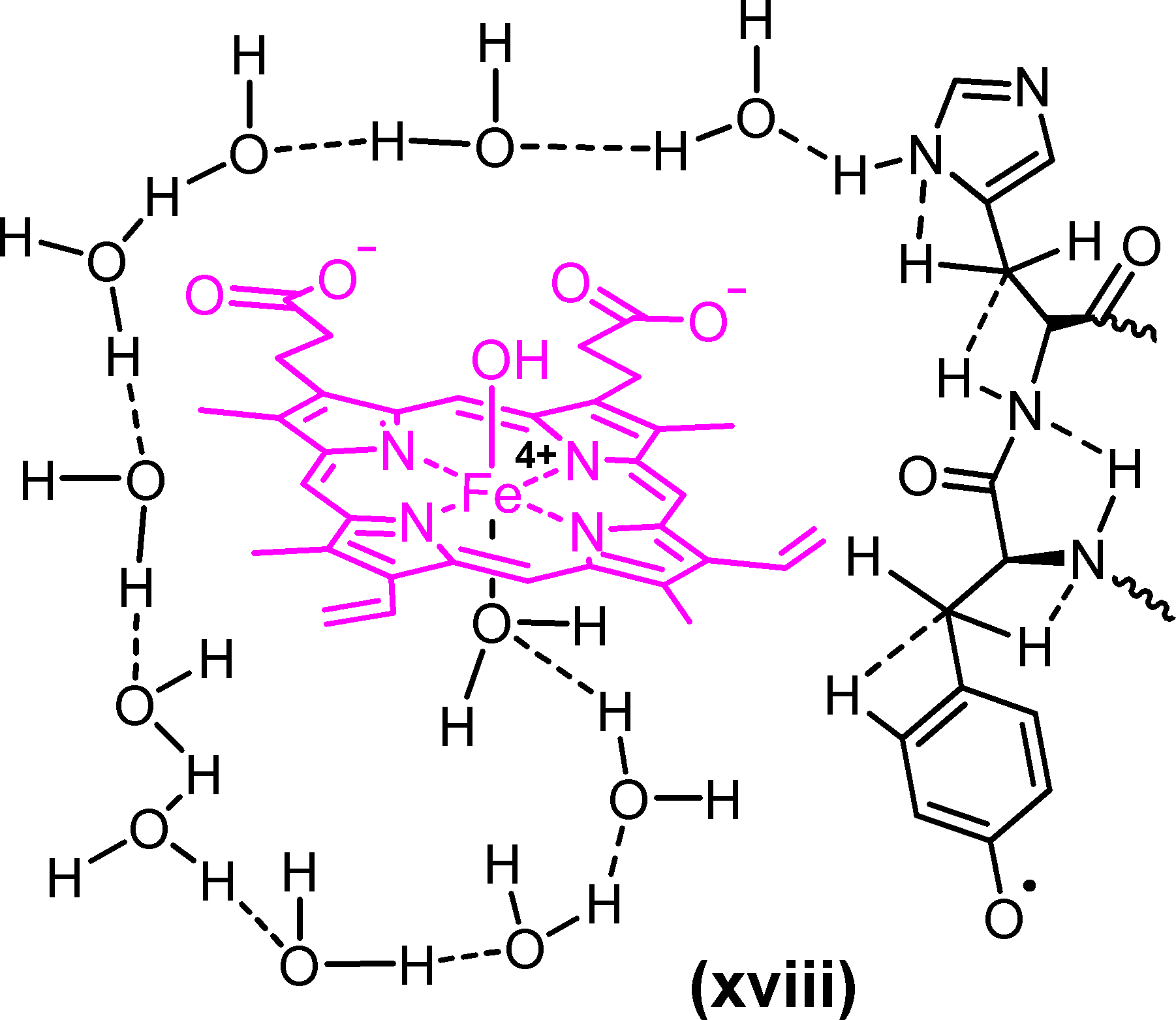
**

**
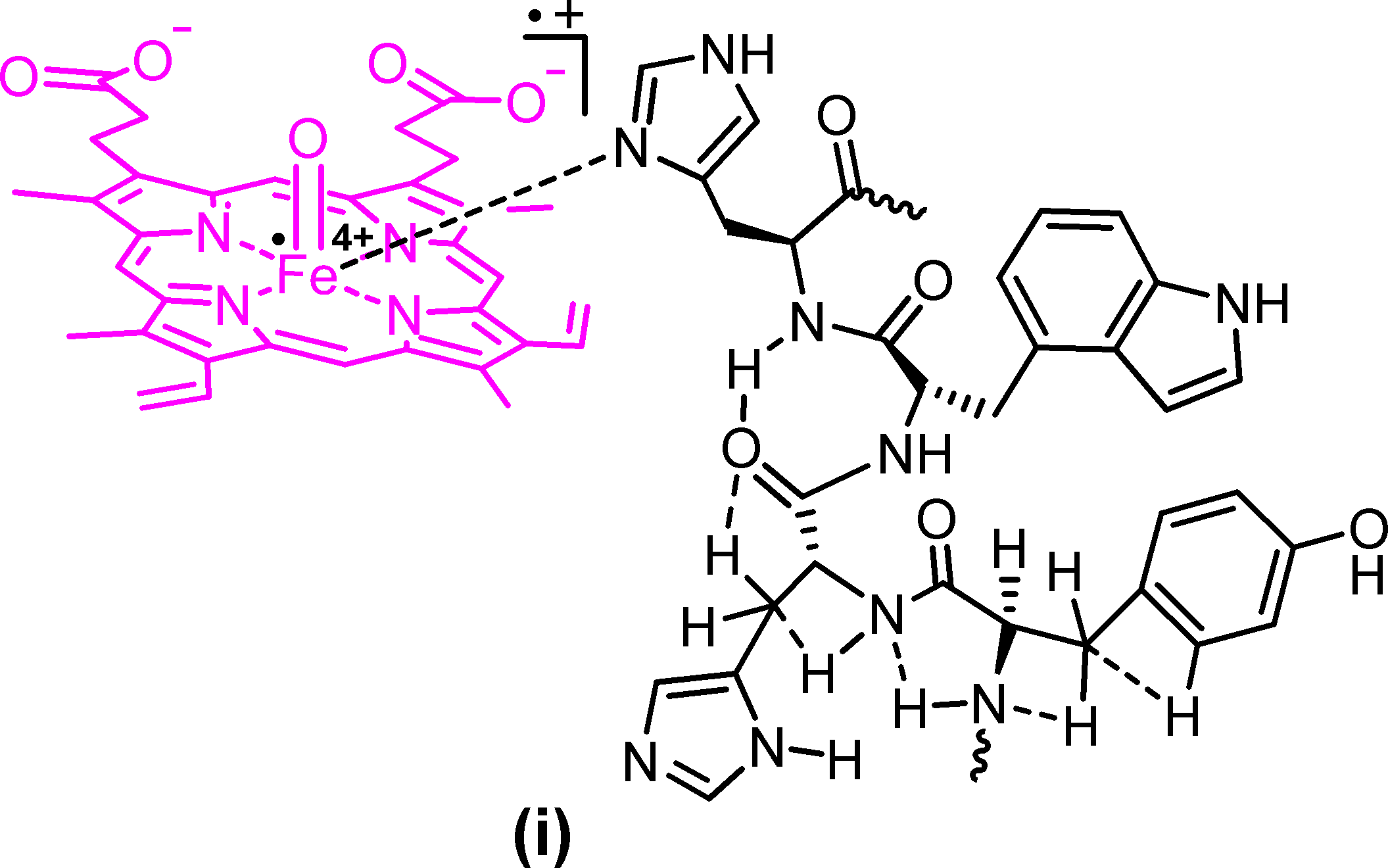

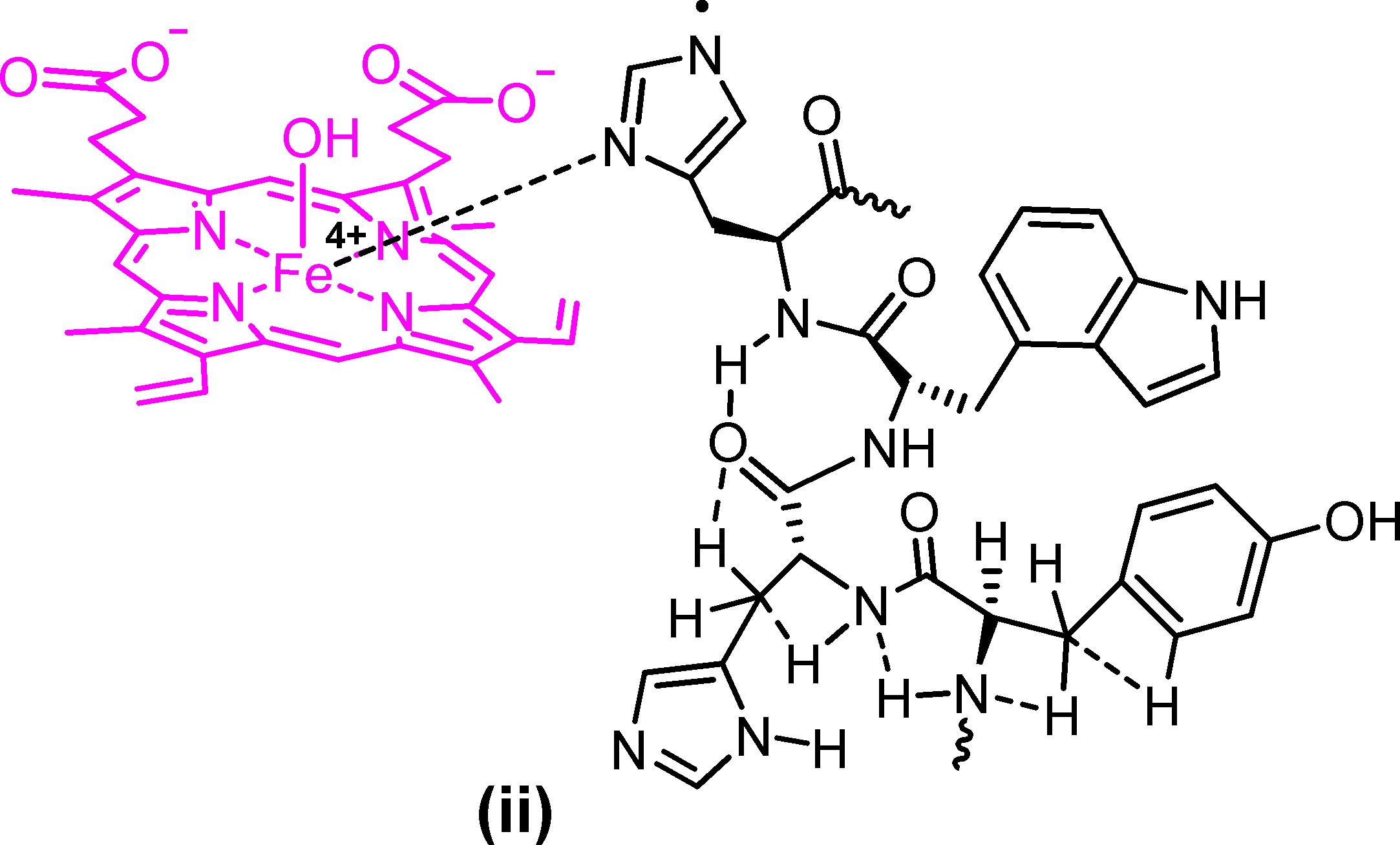
**

**
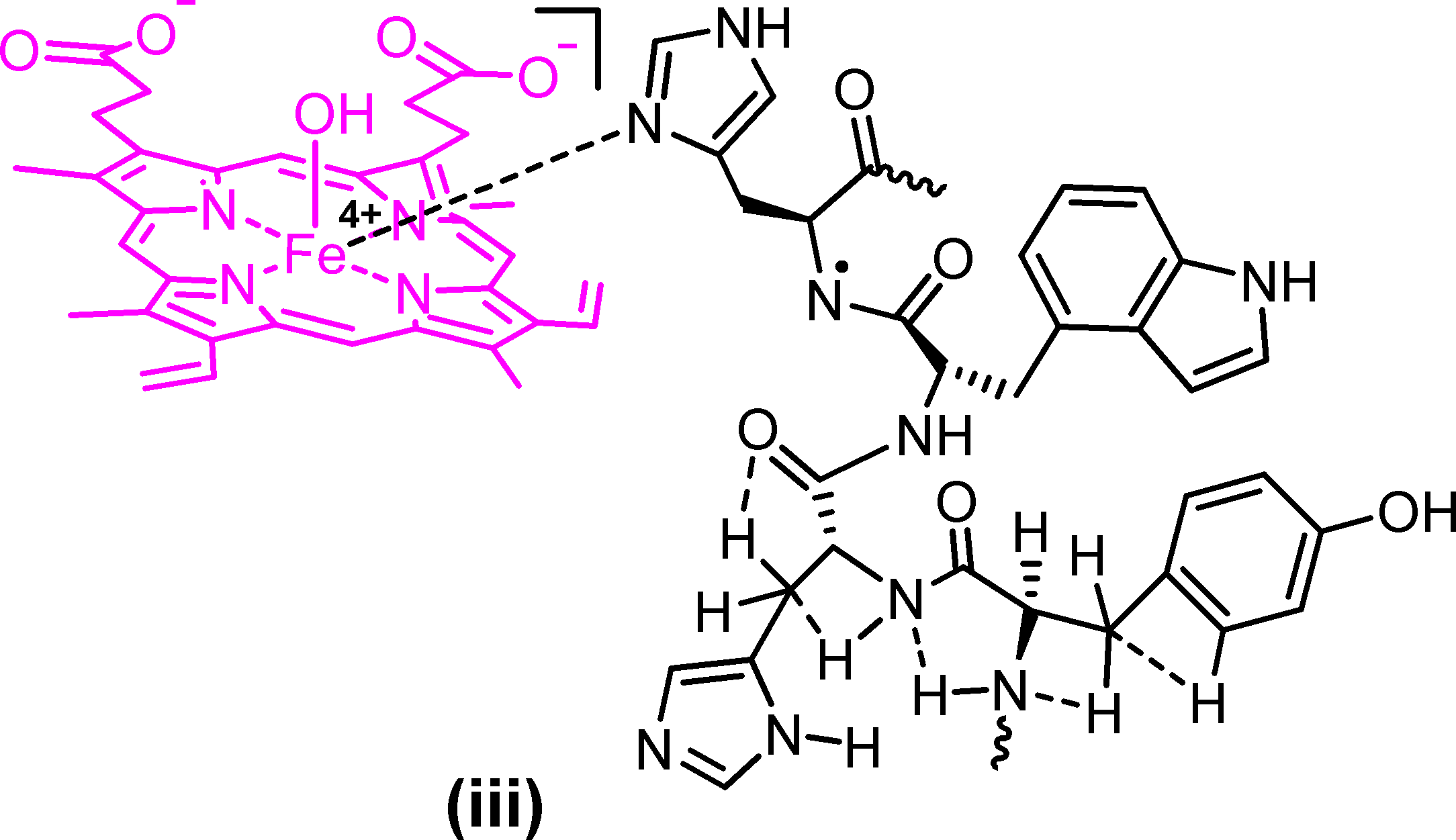

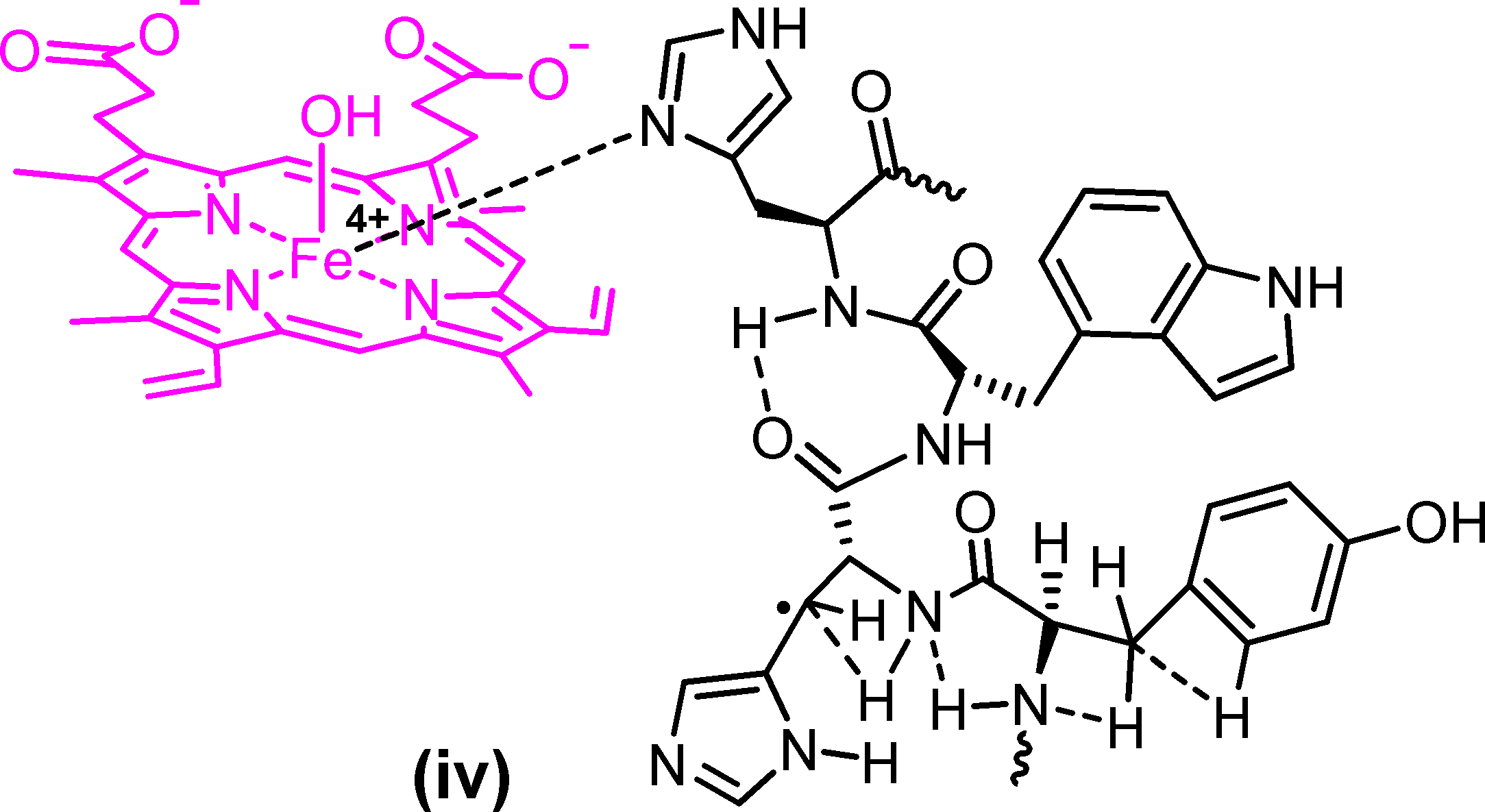
**

**
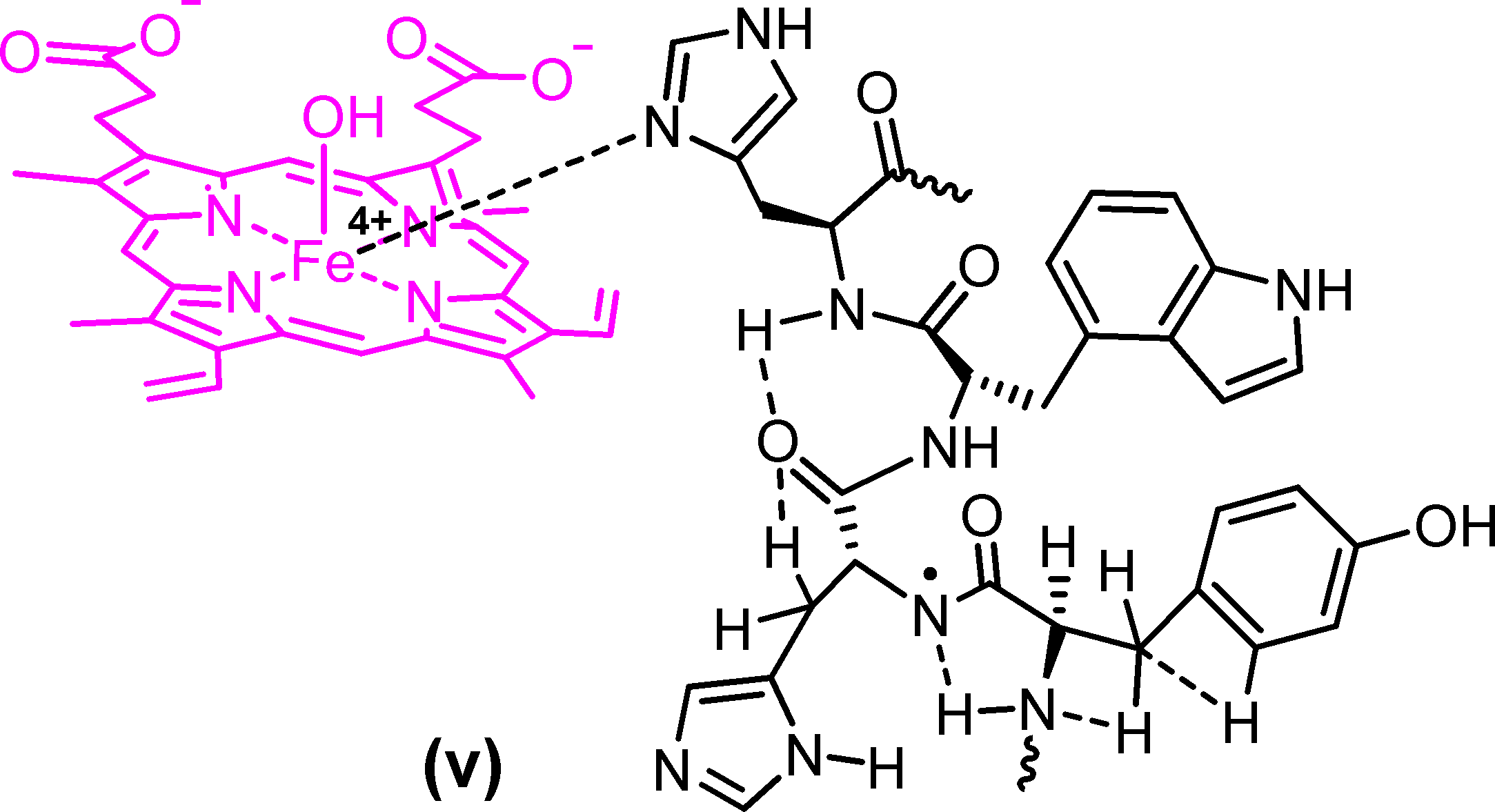

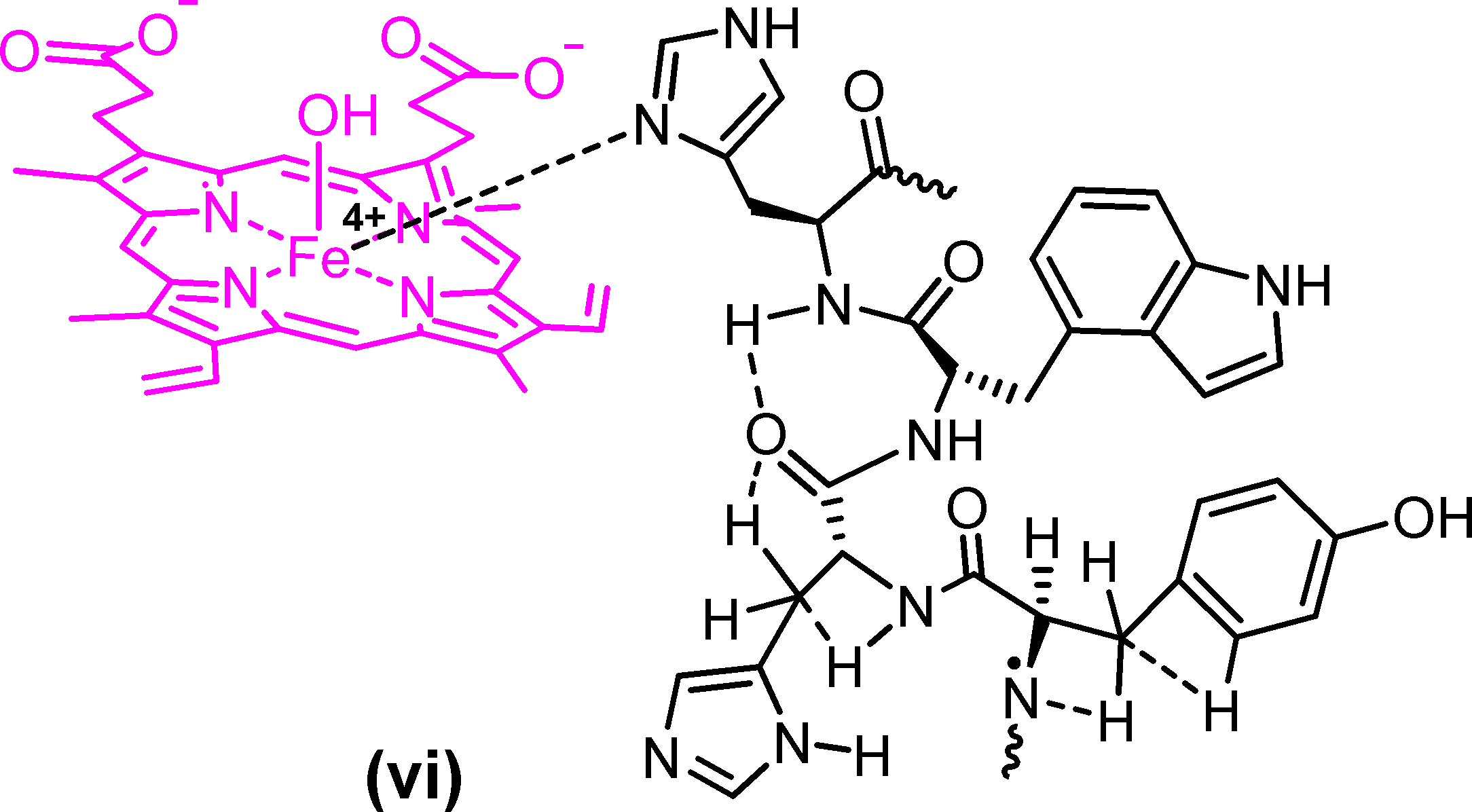
**

**
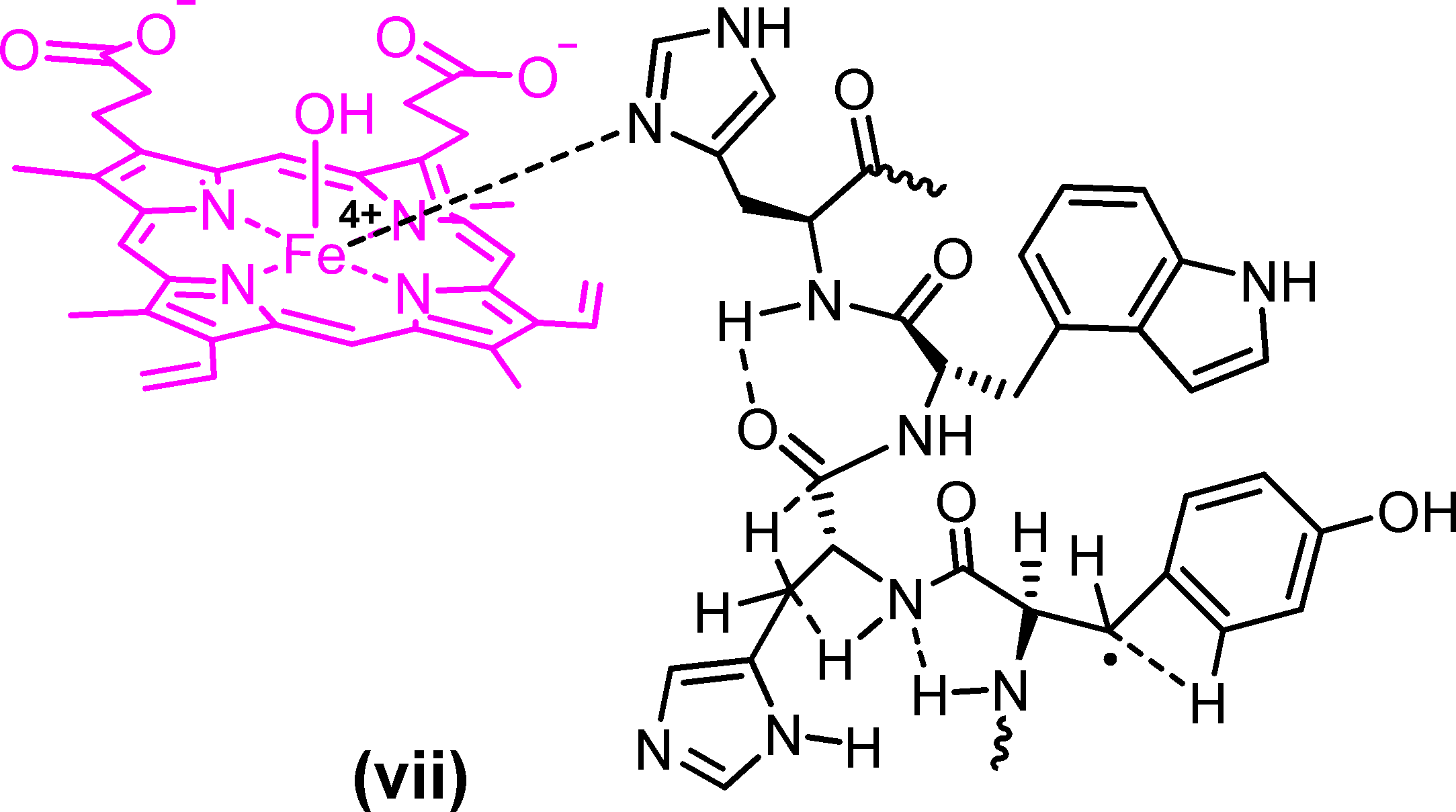

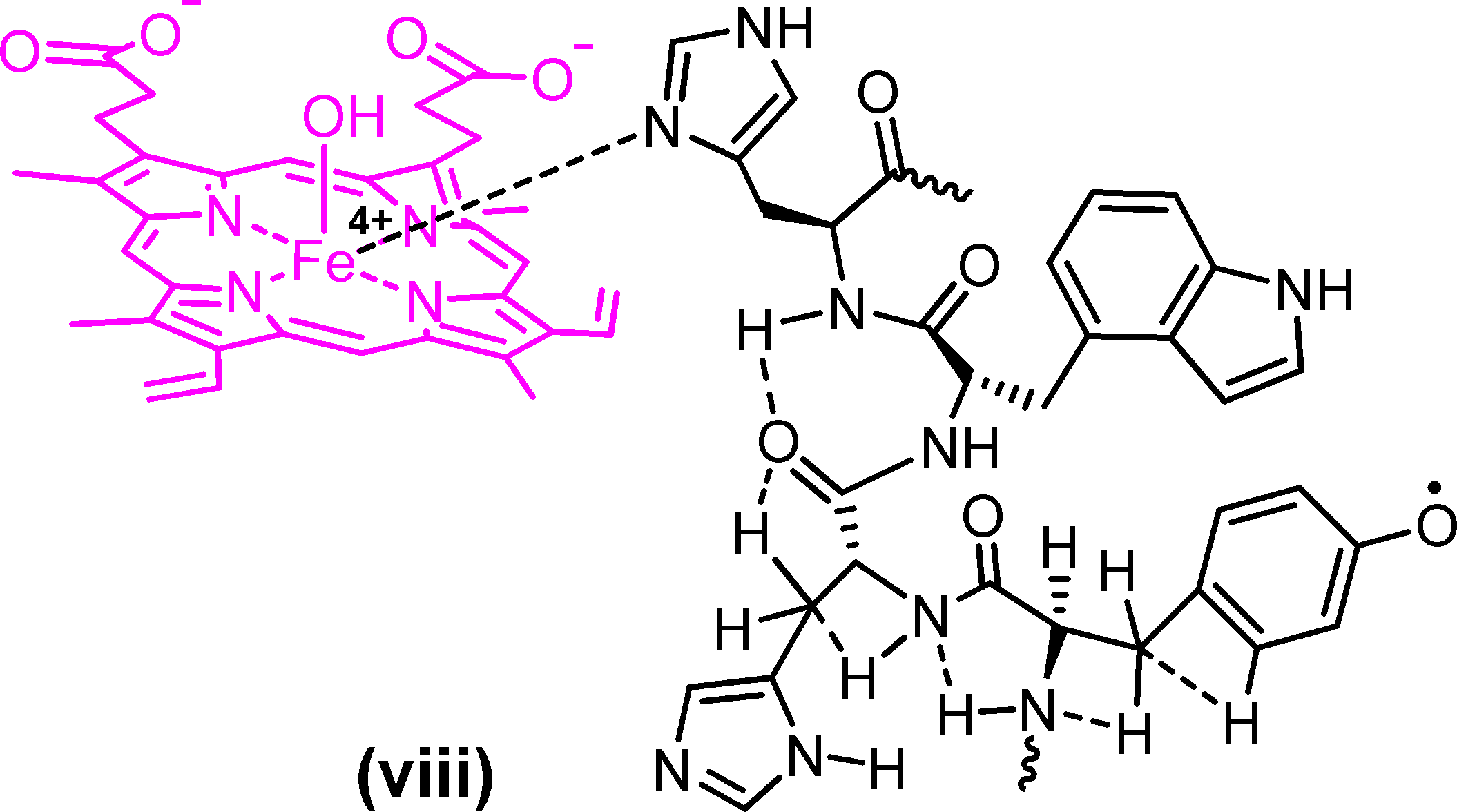
**

**Figure S9**. The radical carrying systems for channel ‘c’ built for calculating the single point energy.

**Table S1**. Single Point energies calculated by generating the radical at various participating atoms of the three electron transfer channels.

Channel- a

| Radical location | | Energy (Hartree) | Relative stability w.r.t. Heme radical, ∆G | |
| --- | --- | --- | --- | --- |
|  |  |  | Hartree | kcal/mol |
| Heme**^∙^** (Fe) | - | -5051.524084* |  |  |
| W-1 | I | -5051.544417 | i – Fe = -0.020 | -12.5 |
| W-2 | Ii | -5051.546261 | ii – Fe = -0.022 | -13.8 |
| W-3 | Iii | -5051.552127 | iii – Fe = -0.028 | -17.5 |
| W-4 | Iv | -5051.551084 | iv – Fe = -0.027 | -16.9 |
| W-5 | V | -5051.552084 | v – Fe = -0.028 | -17.8 |
| W-6 | Vi | -5051.552609 | vi – Fe = -0.028 | -17.9 |
| W-7 | Vii | -5051.553088 | vii – Fe = -0.029 | -18.2 |
| W-8 | Viii | -5051.553725 | viii – Fe = -0.029 | -18.6 |
| W-9 | Ix | -5051.551892 | ix – Fe = -0.028 | -17.4 |
| W-10 | X | -5051.554362 | x – Fe = -0.030 | -19.0 |
| W-11 | Xi | -5051.555289 | xi – Fe = -0.031 | -19.4 |
| T383 N**^∙^**H | Xii | -5051.557709 | xii – Fe = -0.033 | -21.1 |
| L384 N**^∙^**H | Xiii | -5051.558825 | xiii – Fe = -0.034 | -21.8 |
| Y385 N**^∙^**H | Xiv | -5051.562968 | xiv – Fe = -0.038 | -24.4 |
| Y385**^∙^** CH_2_ | Xv | -5051.564757 | xv – Fe = -0.040 | -25.1 |
| Y385**^∙^** OH | Xvi | -5051.566651 | xvi – Fe = -0.042 | -26.3 |

*5 kcal/mol destabilization w.r.t. the system without radical. Centres i – xvi: as per figure 1C

**Channel -b**

| Radical location | | Energy (Hartree) | Relative stability w.r.t. Heme radical, ∆G | |
| --- | --- | --- | --- | --- |
|  |  |  | Hartree | kcal/mol |
| Heme**^∙^** (Fe) | - | -3900.444109* |  |  |
| W-1 | I | -3900.456697 | i – Fe = -0.012 | -7.9 |
| W-2 | Ii | -3900.465145 | ii – Fe = -0.021 | -13.2 |
| W-3 | Iii | -3900.464027 | iii – Fe = -0.019 | -12.5 |
| W-4 | Iv | -3900.465621 | iv – Fe = -0.021 | -13.5 |
| W-5 | V | -3900.465912 | v – Fe = -0.021 | -13.6 |
| W-6 | Vi | -3900.465145 | vi – Fe = -0.021 | -13.2 |
| W-7 | Vii | -3900.463710 | vii – Fe = -0.019 | -12.3 |
| W-8 | Viii | -3900.462913 | viii – Fe = -0.018 | -11.8 |
| W-9 | Ix | -3900.462754 | ix – Fe = -0.018 | -11.7 |
| W-10 | X | -3900.463392 | x – Fe = -0.019 | -12.1 |
| W-11 | Xi | -3900.464826 | xi – Fe = -0.020 | -13.0 |
| H386 (N**^∙^** ring) | Xii | -3900.460045 | xii – Fe = -0.015 | -10.0 |
| H386 (N**^∙^**) | Xiii | -3900.463232 | xiii – Fe = -0.019 | -12.0 |
| H386 (C**^∙^**H) | Xiv | -3900.464348 | xiv – Fe = -0.020 | -12.7 |
| Y385 (N**^∙^**H) | Xv | -3900.466101 | xv – Fe = -0.021 | -13.8 |
| Y385 (C**^∙^**H) | Xvi | -3900.462595 | xvi – Fe = -0.018 | -11.6 |
| Y385 (O**^∙^**) | Xvii | -3900.461161 | xvii – Fe = -0.017 | -10.7 |

*5 kcal/mol destabilization w.r.t. the system without radical. Centres i – xvi: as per figure 1C

**Channel- c**

| Radical location | | Energy (Hartree) | Relative stability w.r.t. Heme radical, ∆G | |
| --- | --- | --- | --- | --- |
|  |  |  | Hartree | kcal/mol |
| Heme**^∙^** (Fe) | - | -4068.123339 |  |  |
| H388(N**^∙^**-ring) | xii | -4068.125092 | -0.001 | -1.1 |
| H388 (N**^∙^**H_2_) | xiii | -4068.131148 | -0.007 | -4.9 |
| H386 (C**^∙^**H) | xiv | -4068.131466 | -0.008 | -5.1 |
| H386 (N**^∙^**) | xv | -4068.131785 | -0.008 | -5.3 |
| Y385 (N**^∙^**H) | xvi | -4068.133699 | -0.010 | -6.5 |
| Y385 (C**^∙^**H) | xvii | -4068.133379 | -0.010 | -6.3 |
| Y385 (O**^∙^**) | xviii | -4068.131944 | -0.008 | -5.4 |

*32 kcal/mol destabilization w.r.t. the system without radical. The trace for channel ‘c’ in the main text showed 5 kcal/mol destabilization that was just to avoid large size of the figure. centres xii – xviii: as per figure 1C in the main text.

**Table S2-i**. p*K*_a_ and the equilibrium constants between the two consecutive steps of the three electron transfer channels. For the water loop, only p*K*_a_^R^ were determined.

| R – X**·**–Y* | p*Ka*^R^ | | | *K*_eq_ | | |
| --- | --- | --- | --- | --- | --- | --- |
|  | a | b | c | a | B | C |
| Fe |  |  |  | 0.3 | 0.2 | 0.3 |
| W-1 | -22.4 | -20.3 | - | 1.0 | 1.2 | - |
| W-2 | -20.1 | -16.7 | - | 1.0 | 0.8 | - |
| W-3 | -17.4 | -15.6 | - | 1.0 | 0.9 | - |
| W-4 | -15.2 | -8.9 | - | 0.9 | 1.0 | - |
| W-5 | -11.0 | -7.3 | - | 1.0 | 0.9 | - |
| W-6 | -8.4 | -6.4 | - | 0.9 | 1.0 | - |
| W-7 | -7.5 | -6.2 | - | 1.0 | 0.9 | - |
| W-8 | -5.2 | -3.4 | - | 1.0 | 1.0 | - |
| W-9 | -4.8 | -2.8 | - | 1.0 | 1.0 | - |
| W-10 | -3.8 | -1.5 | - | 0.9 | 0.9 | - |
| W-11 | -3.4 | -1.1 | - | 1.0 | 1.3 | - |
| Xii | -4.8 | -3.7 | 4.5 | 0.9 | 0.8 | 1.1 |
| Xiii | -5.2 | -3.7 | -8.4 | 1.0 | 0.9 | 0.9 |
| Xiv | -12.6 | -5.7 | -3.5 | 1.0 | 0.9 | 3.3 |
| Xv | -11.1 | 4.7 | -8.1 | 1.0 | 0.9 | 0.3 |
| Xvi | -12.8 | -9.9 | 7.8 | 1.0 | 1.0 | 1.1 |
| Xvii | - | -12.2 | -9.7 | - | 1.1 | 1.1 |
| Xviii | - | - | -11.5 | - | - | 1.0 |

*X carries the radical, R and Y represent group towards heme and peptide. i – xviii: as per figure 1C in the main text.

**Table S2-ii**. p*K*_a_ over the peptide centres of the channel ‘a’, ‘b’ and ‘c’.

| Channel / group | | p*K*_a_  p*K*_a_^O^ p*K*_a_^R^ | |
| --- | --- | --- | --- |
| a | NH(T383) | 10.1 | -4.8 |
|  | NH(L384) | 9.5 | -5.2 |
|  | NH(Y385) | 10.2 | -12.6 |
|  | βCH(Y385) | 6.0 | -11.1 |
|  | OH(Y385) | 5.4 | -12.8 |
|  |  |  |  |
| b | NH(H386) | 12.4 | -3.7 |
|  | CH(H386) | -0.8 | -3.7 |
|  | NH(H386) | 9.3 | -5.7 |
|  | NH(Y385) | 6.5 | 4.7 |
|  | βCH(Y385) | 2.9 | -9.9 |
|  | OH(Y385) | 1.2 | -12.2 |
|  |  |  |  |
| c | δN(H388) | 5.0 | 4.5 |
|  | NH(H388) | 11.5 | -8.4 |
|  | βCH(H386) | 2.4 | -3.5 |
|  | NH(H386) | 11.8 | -8.1 |
|  | NH(Y385) | 5.4 | 7.8 |
|  | βCH(Y385) | -0.6 | -9.7 |
|  | OH(Y385) | 0.9 | -11.5 |

**Procedure for calculating p*K*_a_**

The p*K*_a_ calculations were performed using Jaguar, version 8.8, Schrӧdinger, LLC, New York, 2015. A series of calculations on the protonated form and on the deprotonated form of each of the participating group of the three channels were performed followed by an empirical correction. The atom or atoms whose p*K*_a_ values are calculated were specified in p*K*_a_ atom cell in the input tab. p*K*_a_ atom was selected in such a manner that it should be the acidic hydrogen atom in an acid, or the basic atom in a base. The method used for accelerating the convergence SCF calculations was Direct Inversion in the Iterative subspace (DIIS) at 2000 iterations and water was selected as the solvent. After finishing the settings, job was run and after the job completion, p*K*_a_ values for each atom were added to the structure in the maestro output file.

Five participating groups of channel ‘a’, six participating groups of channel ‘b’ and seven groups of channel ‘c’ were selected one by one in the protonated as well as the deprotonated form for calculating their respective p*K*_a_ (Figure S11). The p*K*_a_ for the deprotonated atoms are shown in Table 1 in the main text.

**
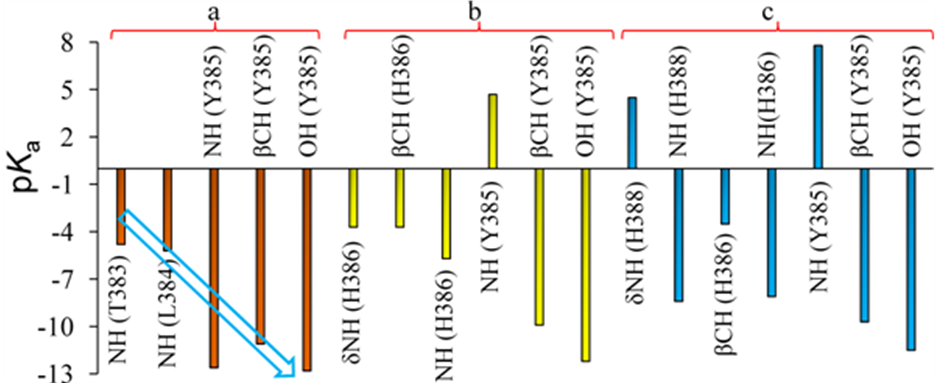
**

**Figure S10**. p*K*_a_ of the participating groups of the three electron-transfer channels in their protonated forms.

**UV-vis spectral studies for T383A, H386V mu COX-2**. The enzymatic reaction was performed as per the enzyme immunoassay kit protocol and the UV-vis spectra were recorded on BIOTEK Synergy H1 Hybrid Reader in bio cell of 1 cm path length with 1 mL volume at different time intervals. The band at 610 nm was restored within 5 min of the reaction (addition of arachidonic acid to the enzymatic reaction).

**
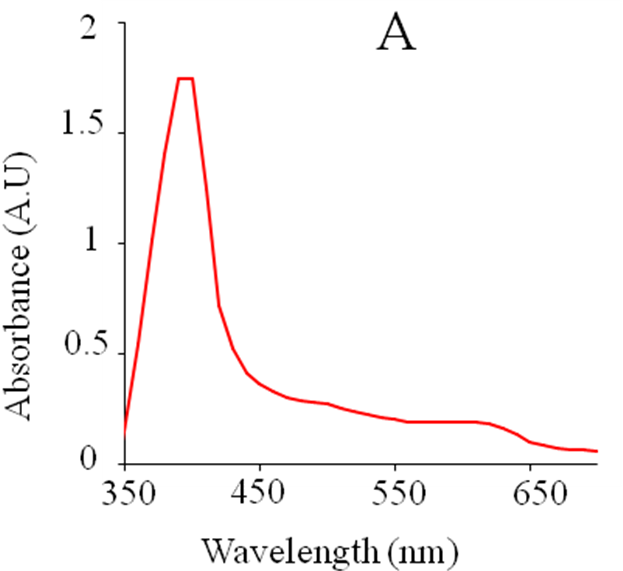

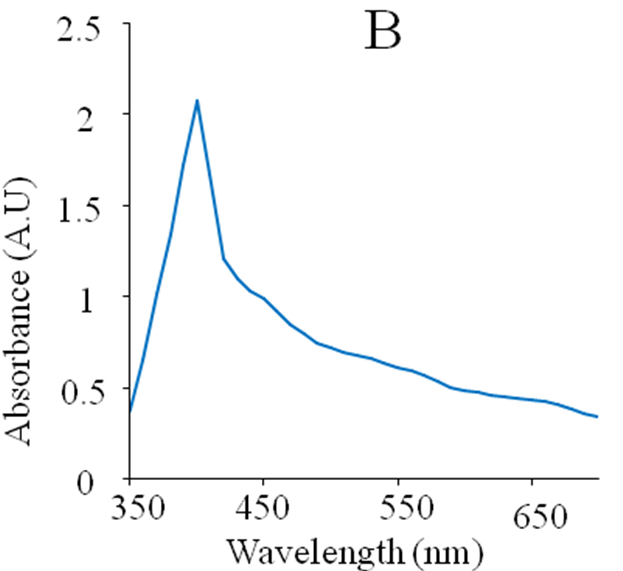
**

**
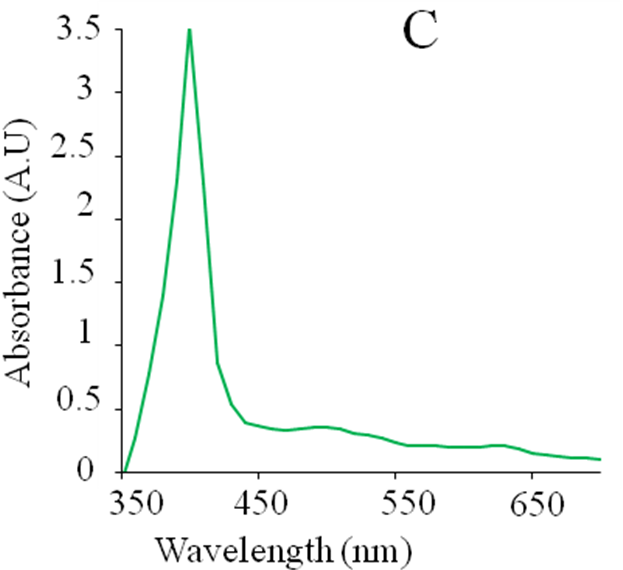
**

**Figure S11**. UV-vis spectra of of T383A, H386V mu COX-2 reaction having (A) no AA, (B) with AA, (C) after 5 min of AA addition.

**
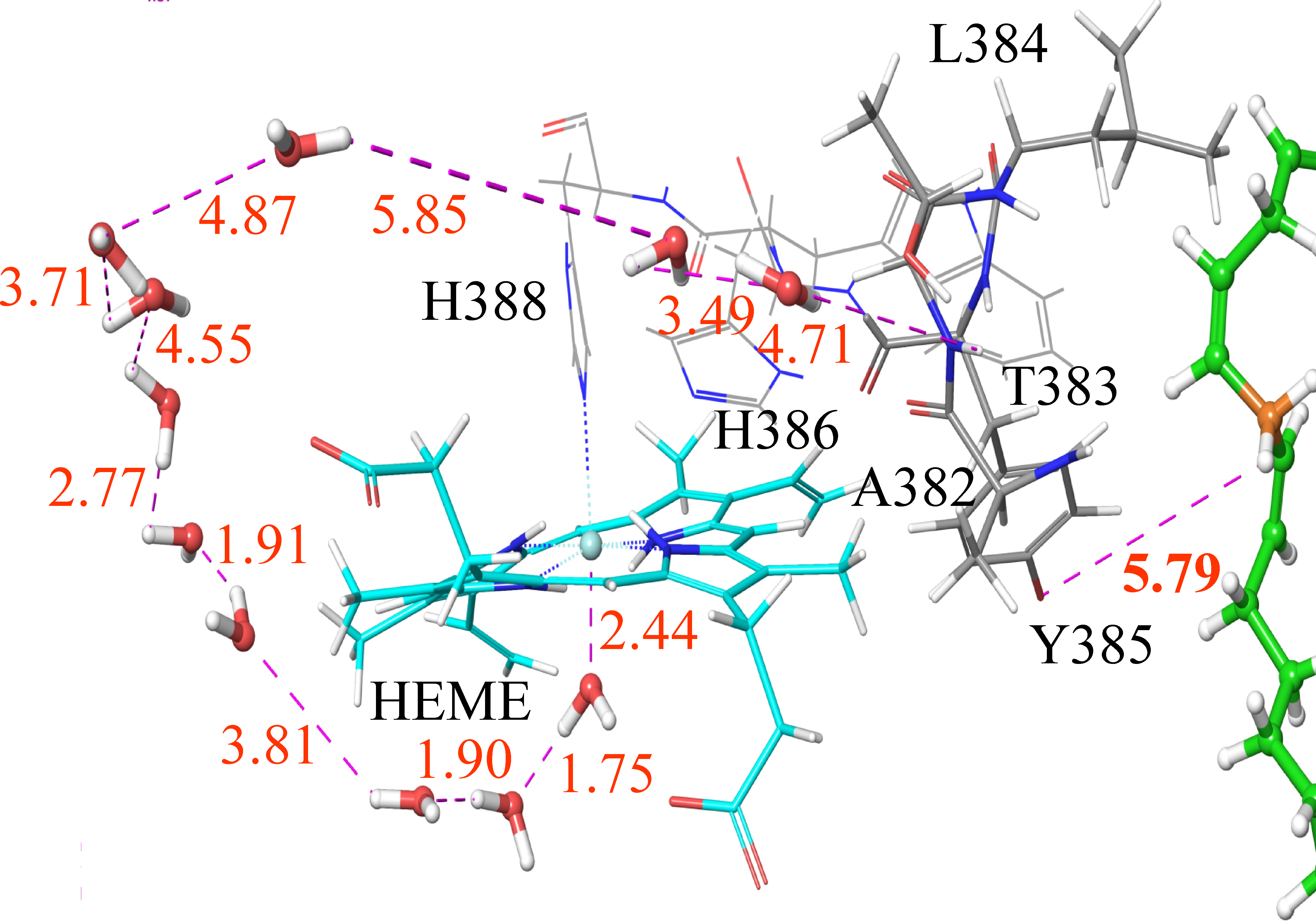
**

**Figure S12**. MD of N382A COX-2 – AA – heme complex showing the alteration in the H-bonding of W-11 with T383 on mutating N382. The distance between W-11 and NH of T383 is raised to 4.71 Å against the 3.30 Å in wt COX-2.

**
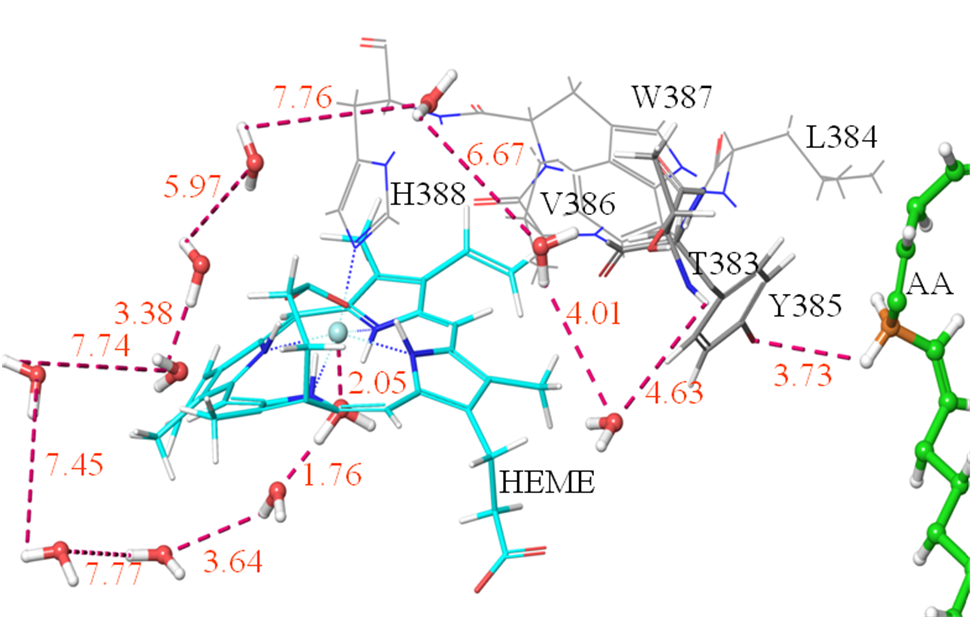
**

**Figure S13**. MD of H386V COX-2 – AA – heme complex showing the alteration in the H-bonding of W-11 with T383. The distance between W-11 and NH of T383 is raised to 4.63 Å against the 3.30 Å in wt COX-2 and the water channel is also disturbed.

**Enzyme activity of the wt and mu COX-2**. The arachidonic acid metabolic activity of the wt- as well as the mu- COX-2 was checked by the enzyme immunoassay kits.^2^ The recombinant proteins were purchased from Merck, Sigma and Cayman Chemical Company. All the reactions of the enzyme immunoassays were performed as per the kit protocol except the use of various mu COX-2. The activity of the mu COX-2 w.r.t. the wt COX-2 was determined by calculating the amount of PGE2 generated by each mu COX-2 and comparing with the PGE_2_ generation by wt COX-2. UV-vis spectra were recorded on BIOTEK Synergy H1 Hybrid Reader.

***K*_m_ calculations**. The Michaelis Menten constants of each of the mu COX-2 as well as the wt COX-2 for arachidonic acid were determined by monitoring the formation of prostaglandins as a function of arachidonic acid concentration. All the experiments were performed at pH 7.0 as per the protocol of enzyme immunoassay kit^2^ but the concentration of arachidonic was varied and the formation of prostaglandins was noted. 1/V_o_ and 1/[AA] were determined from the absorbance spectra.^3^ K_m_ was determined from the slop of the plot between 1/V_o_ and 1/[AA]. K_m_ = slope x V_max_ where V_max_ was intercept on y- axis.


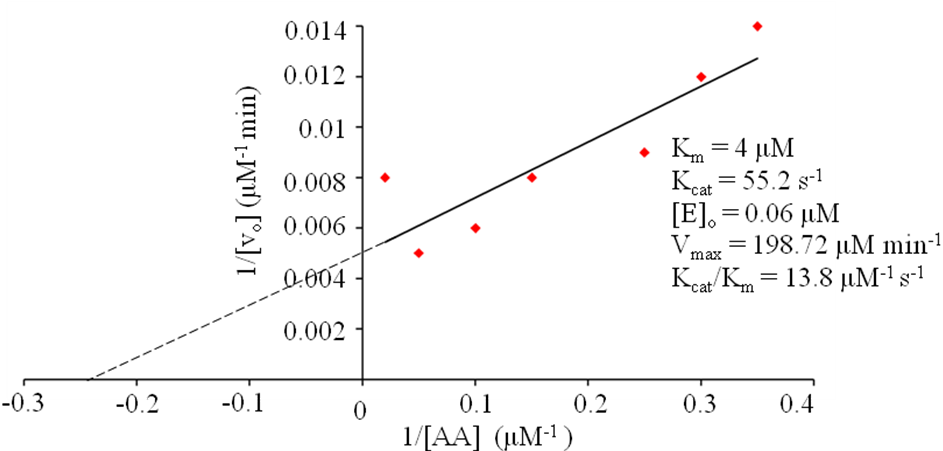


**Figure S14**. Kinetic parameters of wt COX-2.


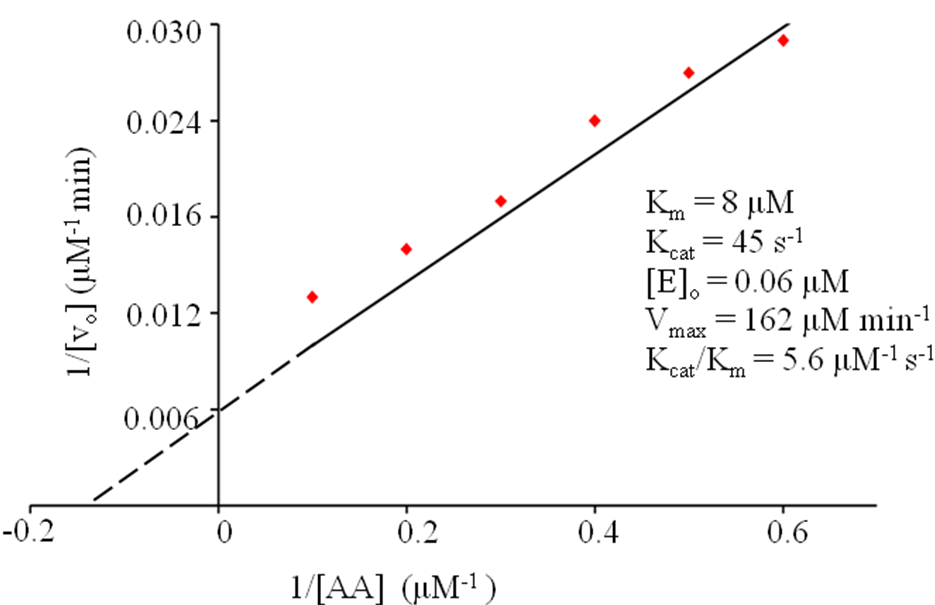


**Figure S15**. Kinetic parameters of F381A COX-2.


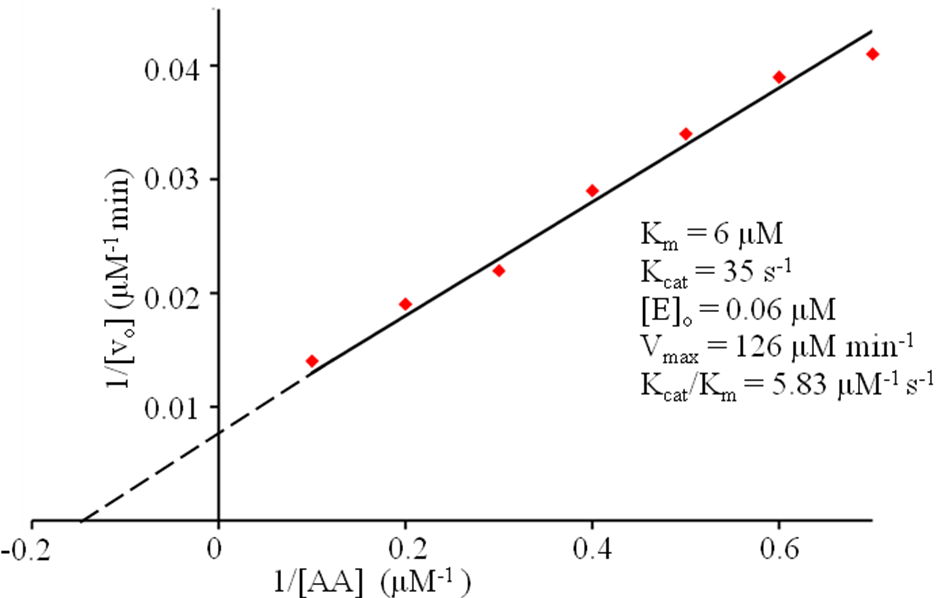


**Figure S16**. Kinetic parameters of N382V COX-2.


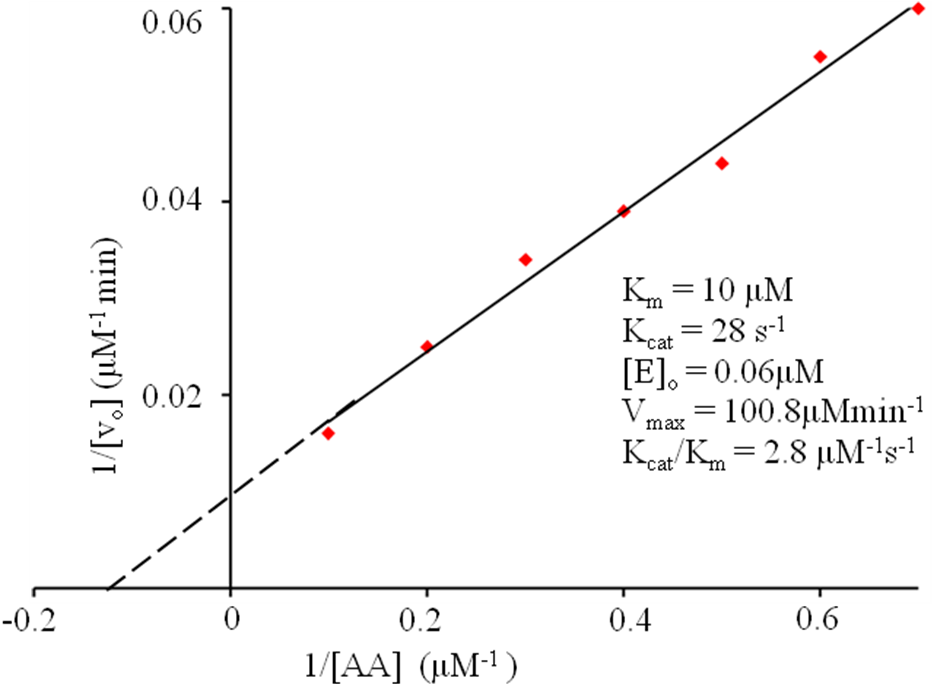


**Figure S17**. Kinetic parameters of T383A COX-2.

**
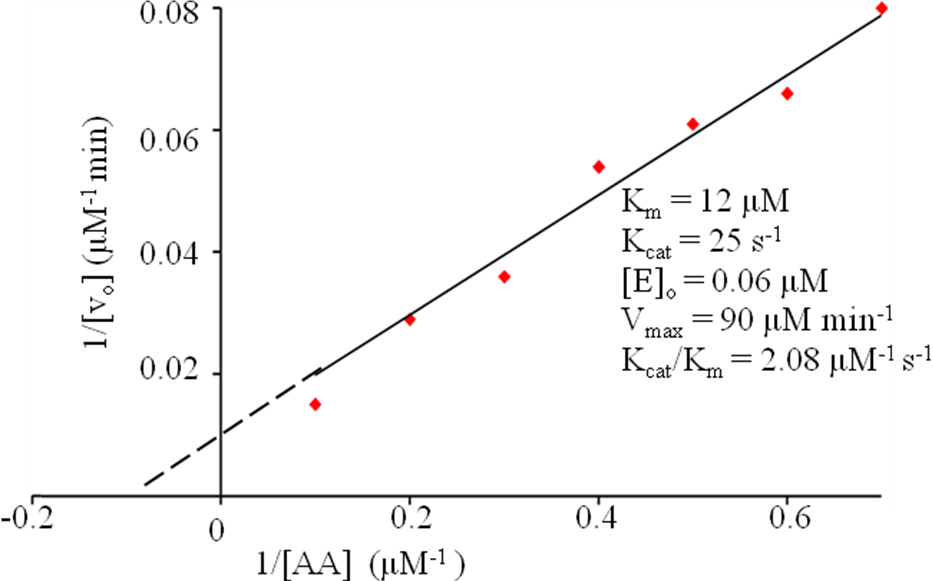
**

**Figure S18**. Kinetic parameters of L384F COX-2.

**
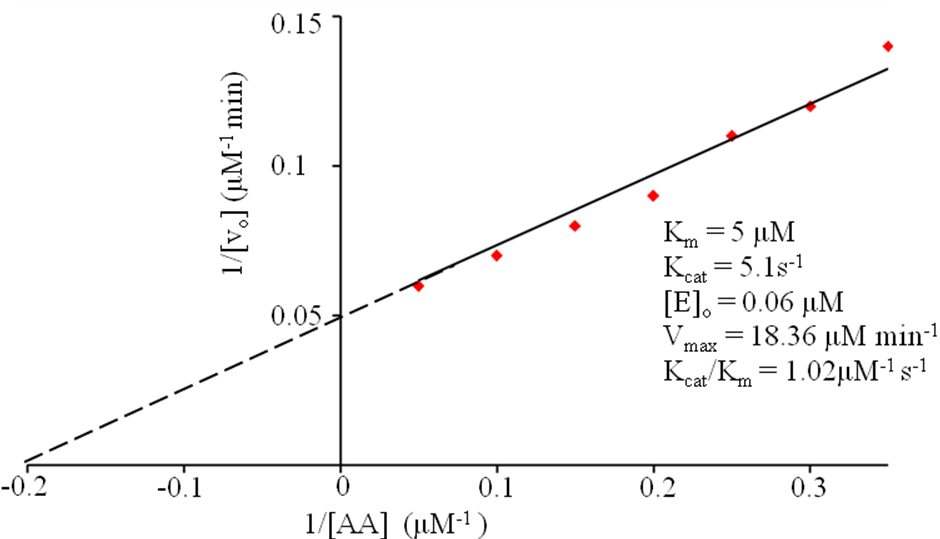
**

**Figure S19**. Kinetic parameters of Y385F COX-2.

**
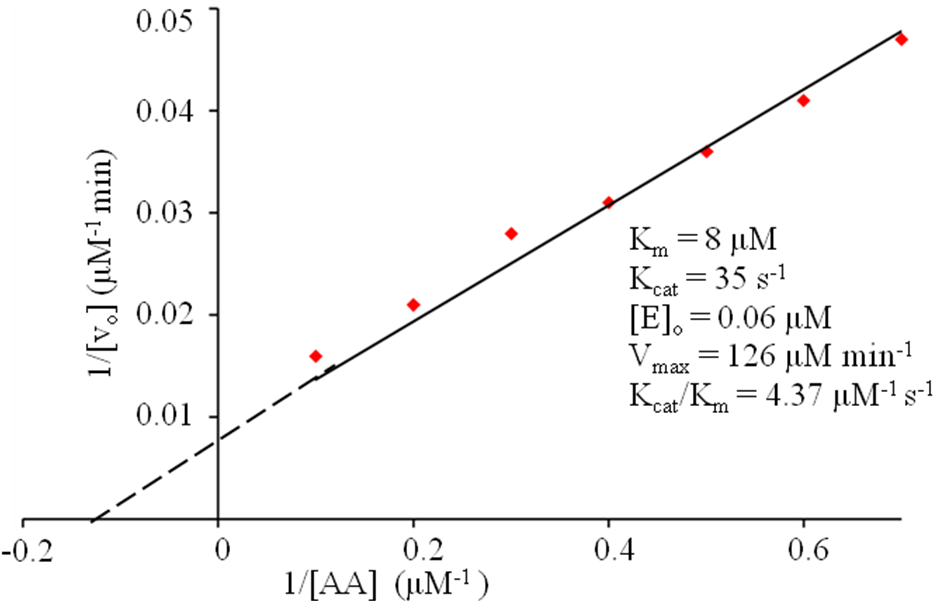
**

**Figure S20**. Kinetic parameters of H386V COX-2.

**
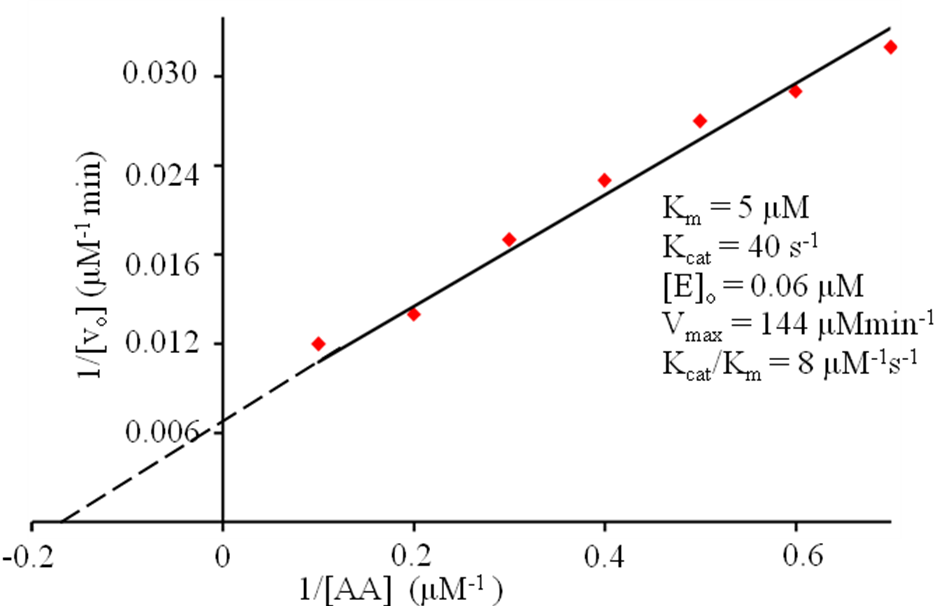
**

**Figure S21**. Kinetic parameters of H388V COX-2.

**
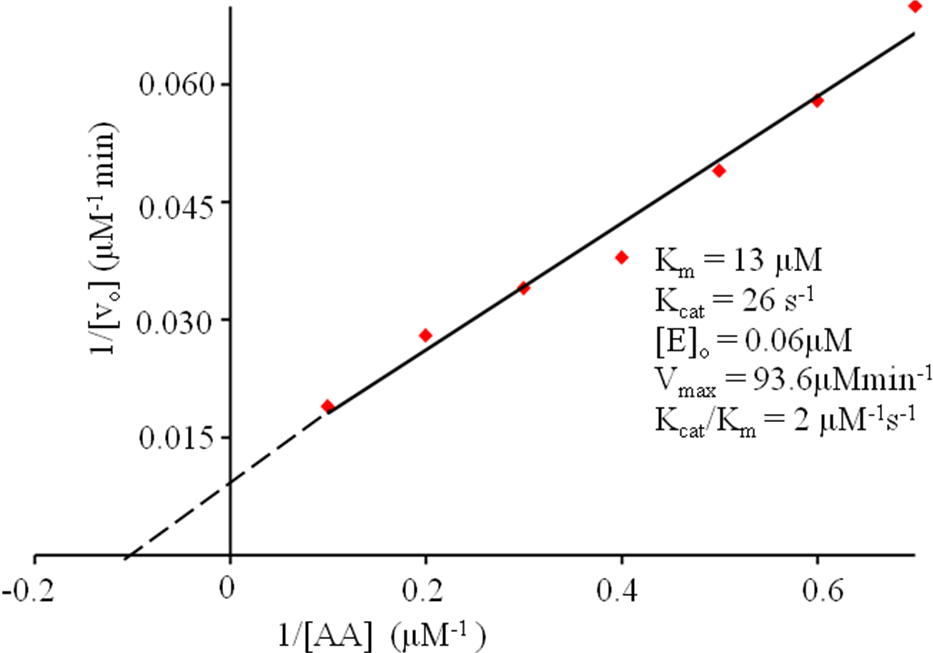
**

**Figure S22**. Kinetic parameters of N382V, H386V COX-2.

**
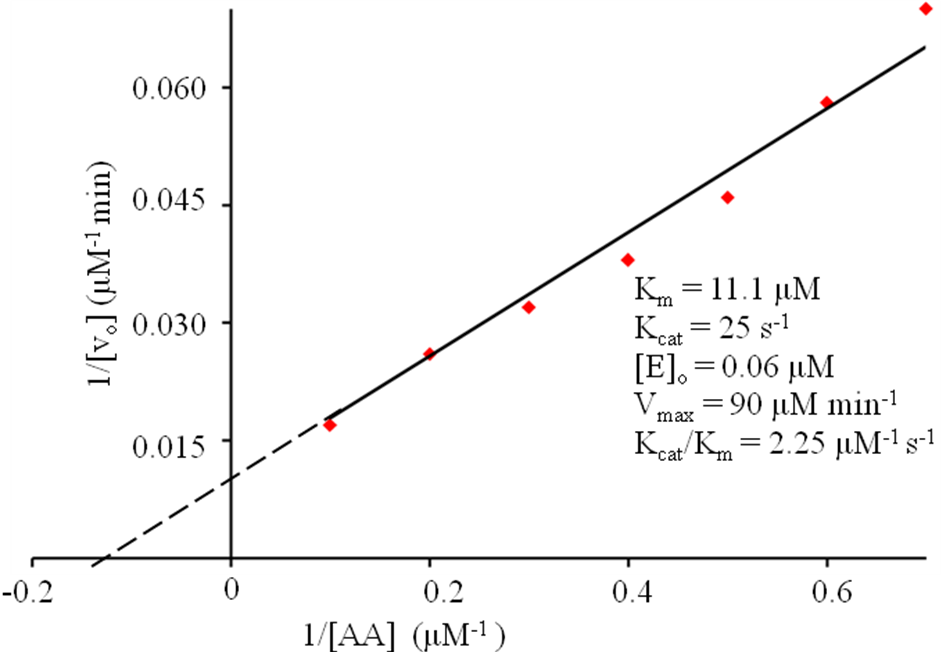
**

**Figure S23**. Kinetic parameters of H388V, H386V COX-2.

***K*_cat_/*K*_m_ vs Temperature studies**. The kinetic constants were determined as mentioned in the previus section but the reactions were performed at different temperatures.

**Table S3**. Change of kinetic constants with temperature.

| **Temp**  **(^o^C)** | ***K*_cat_/*K*_m_** | | | | |
| --- | --- | --- | --- | --- | --- |
|  | **Entry as per Table 1 in the main text** | | | | |
|  | **1** | **3** | **4** | **5** | **8** |
| 25 | 13.8 | 5.8 | 2.8 | 2.0 | 8.0 |
| 35 | 13.6 | 5.5 | 2.8 | 2.1 | 7.5 |
| 45 | 8.5 | 3.1 | 1.5 | 1.4 | 4.3 |
| 55 | 3.7 | 1.8 | 0.9 | 0.8 | 2.1 |
| 60 | 3.4 | 1.5 | 0.7 | 0.8 | 1.9 |


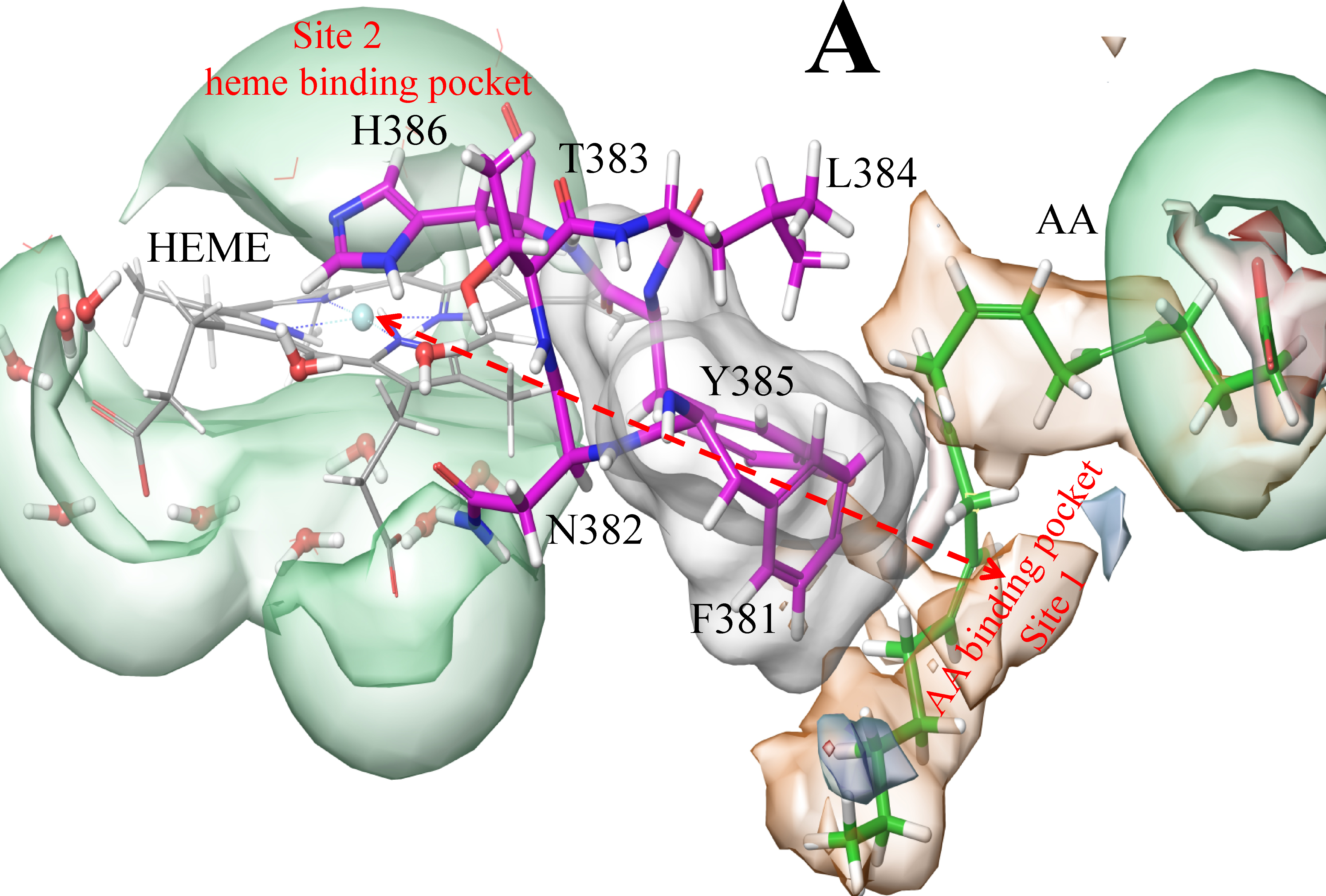
**
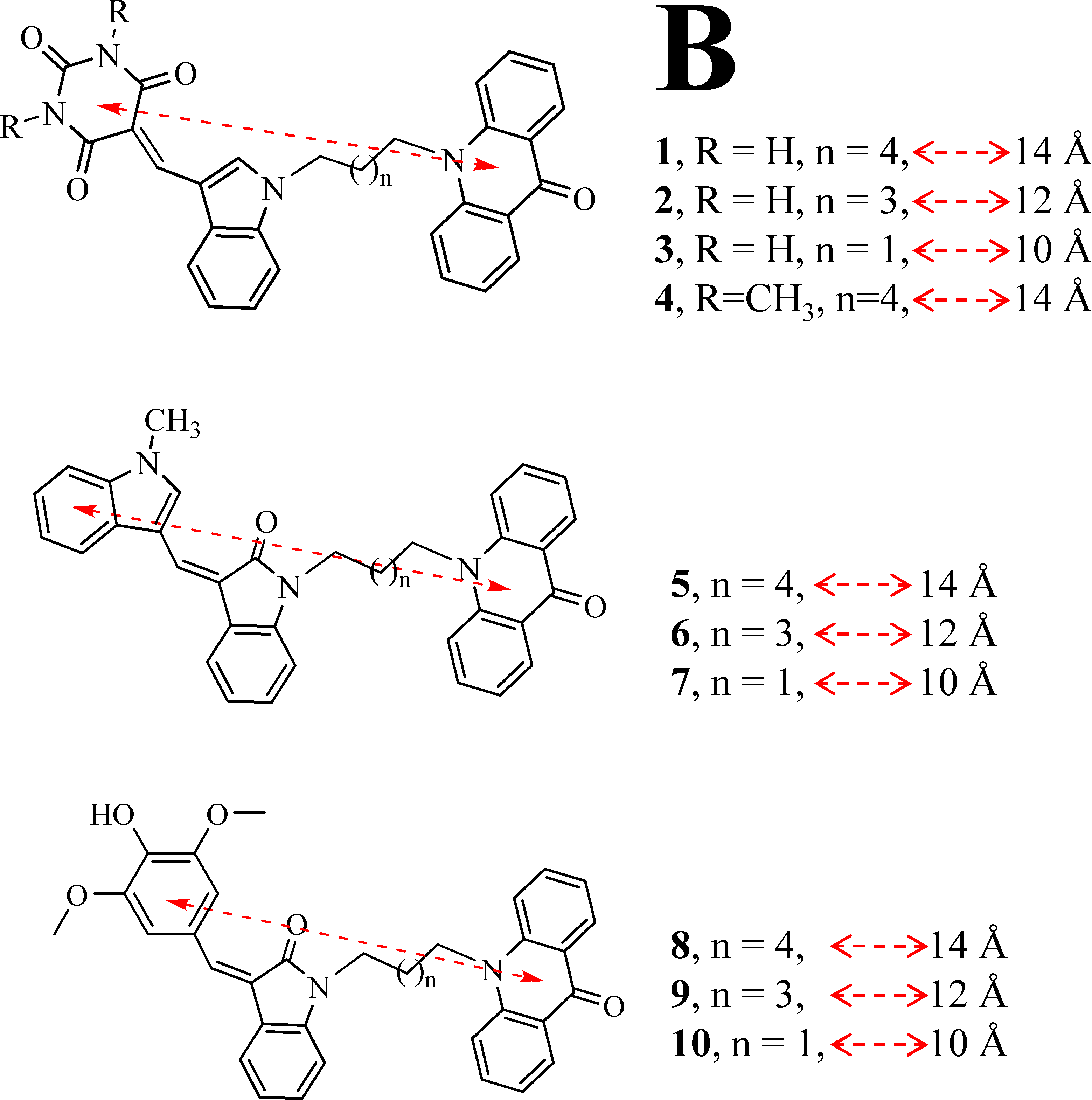
**


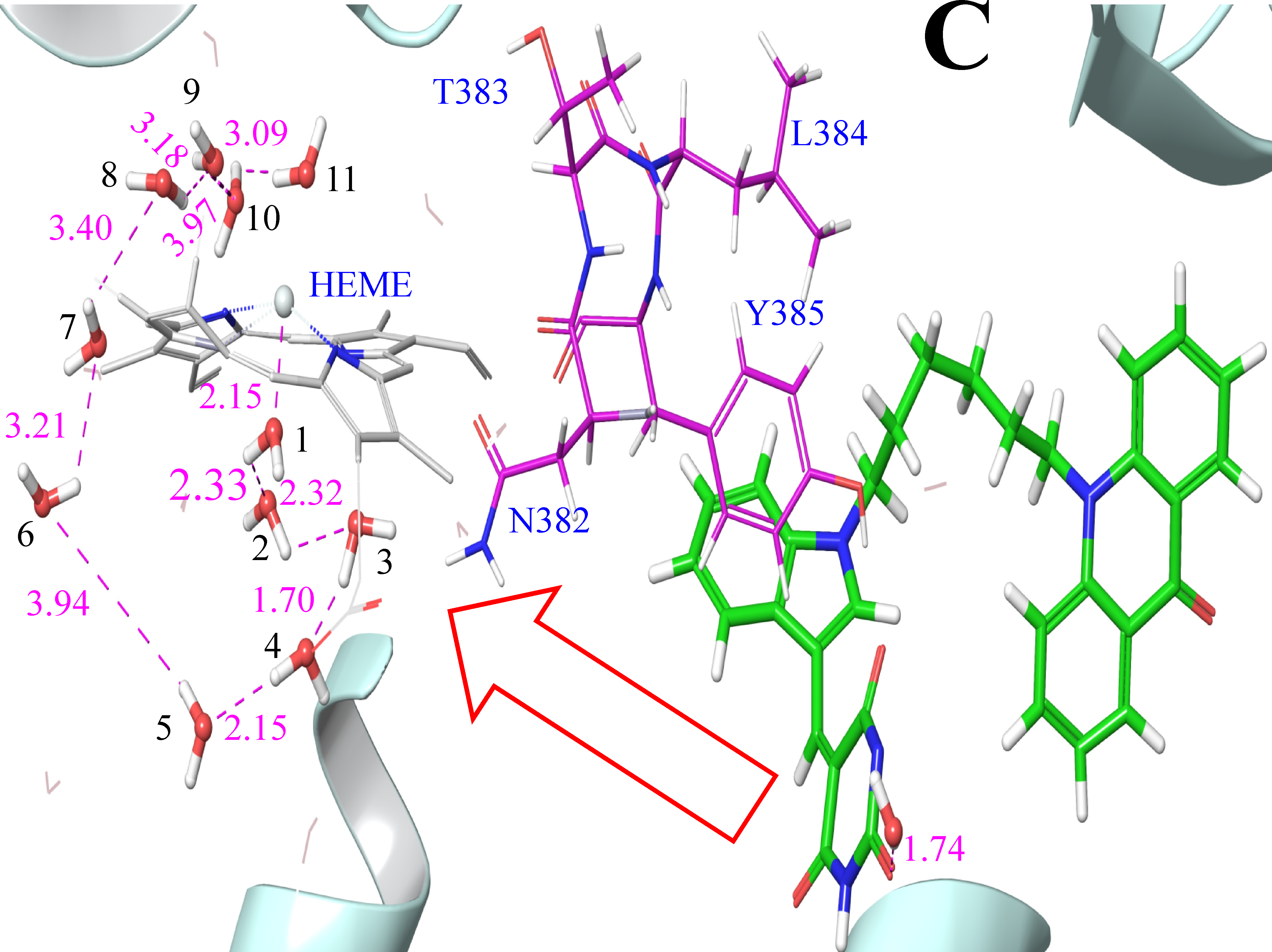

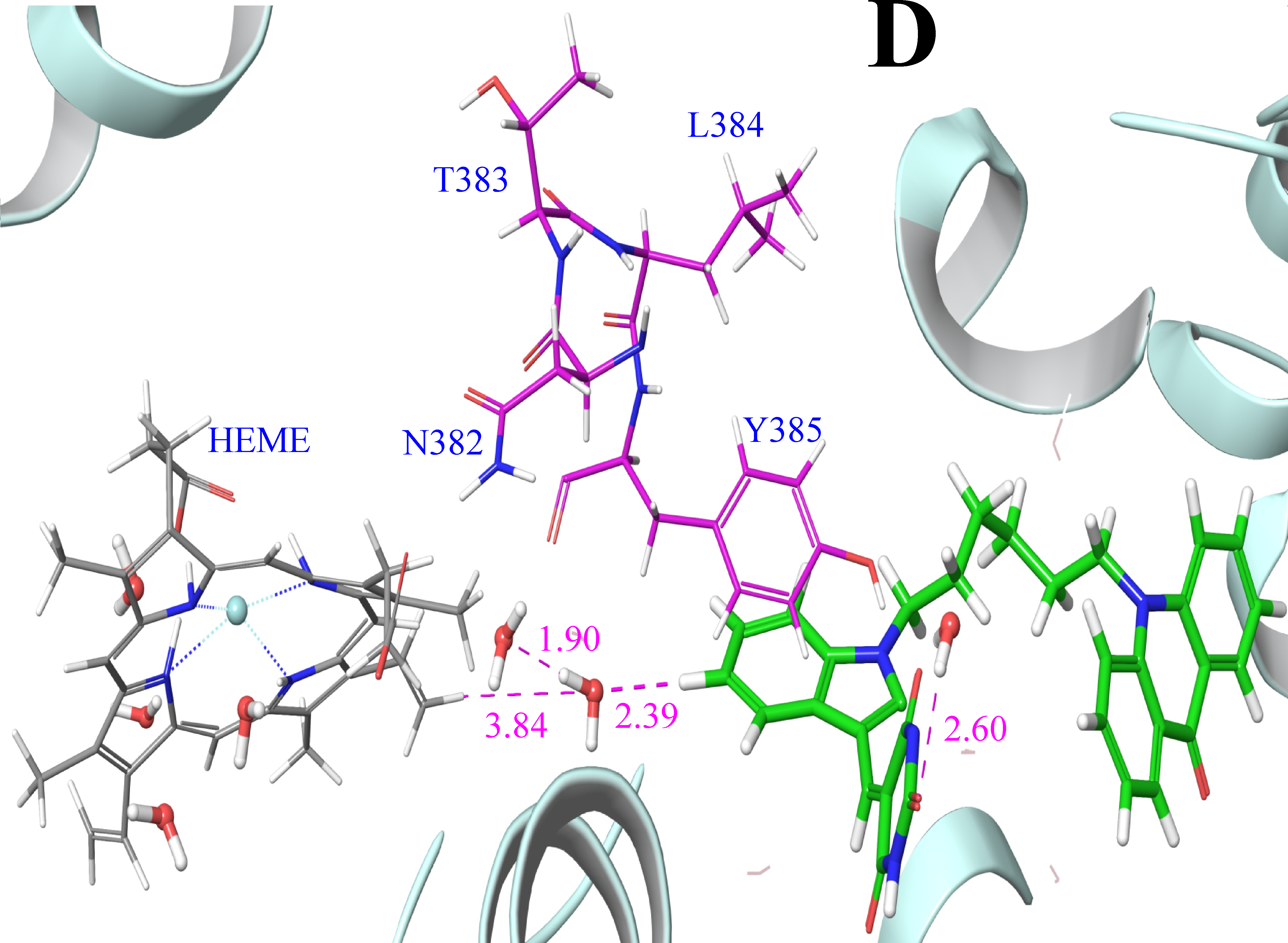


**Figure S24**. Distance between the arachidonic acid binding pocket and the heme binding site of COX-2 (red arrow) is 14 Å. The yellow part represents hydrophobic region whereas green is the hydrophilic region. (B) Rationally designed molecules against COX-2. MD of COX-2 – AA – heme – compound **1** complex showing: (C) initial placement of the compound w.r.t. the water loop and (D) after 5 ns of MD. The indole –pyrimidine part approached the water loop and water molecules 2 and 3 of the loop made H-bond interactions with compound **1** disturbing the water channel.

**HOMO-LUMO Analysis**. Keeping in view the hydrophobic and hydrophilic interactions of the molecule through acridone and pyrimidine moieties, respectively, the HOMO-LUMO analysis of the ligand and the enzyme–substrate/ligand complex was performed so that the change in fluorescence of compound **1**, **13d** and **15d** in the presence of COX-2 was justified. The geometry optimization and calculations for the compounds were performed by using density functional theory (DFT) level of Jaguar-Schrodinger. The highest occupied molecular orbital (HOMO) and the lowest unoccupied molecular orbital (LUMO) energies at B3LYP/6-31G** level were calculated. PBF solver was used for optimization of structure in both the gaseous and solution phase. In compound **1**, HOMO maps were located on the acridine part of the compound. Docking studies of compound **1** with COX-2 also showed the involvement of acridine moiety in protein-ligand interactions. The LUMO maps were located on the indole-pyrimidine moiety of the compound. As predicted from the molecular modelling studies, the acridone part of compound **1** is placed in the hydrophobic pocket of COX-2 and it exhibits π-π interactions with Y348, W385 and W387; the transfer of electron between HOMO of acridone and the Y348/W385/W387 may be responsible for the quenching of compound **1** fluorescence in the presence of the enzyme. Moreover, LUMO of **1** also interact with the water loop. While HOMO of **13d** and **15d** do interact with W387 but their LUMO did not interact with the water loop. The lowest unoccupied molecular orbital/highest occupied molecular orbital (LUMO/HOMO) energy gaps ΔEg for compound **1** were calculated (Table S4, Figure S26).

**Table S4. Calculated energy values of 1 in gas and solvent phase (water).**

| **SCF/B3LYP/6-31G **** | **Gas** | **H_2_O** |
| --- | --- | --- |
| E_total_ (Hartree) | -1755.75 | -1755.86 |
| E_HOMO_ (eV) | -2.08 | -5.55 |
| E_LUMO_ (eV) | -0.387 | -2.56 |
| ΔE_LUMO-HOMOgap_ (eV) | 1.693 | 2.99 |
| E_HOMO-1_ (eV) | -2.25 | -5.74 |
| E_LUMO+1_(eV) | 0.687 | -1.83 |
| ΔE_LUMO+1-HOMO-1gap_ (eV) | 2.937 | 3.91 |

**
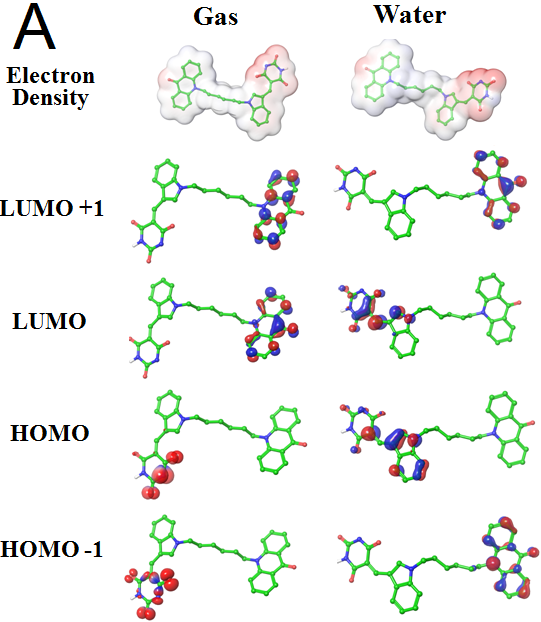

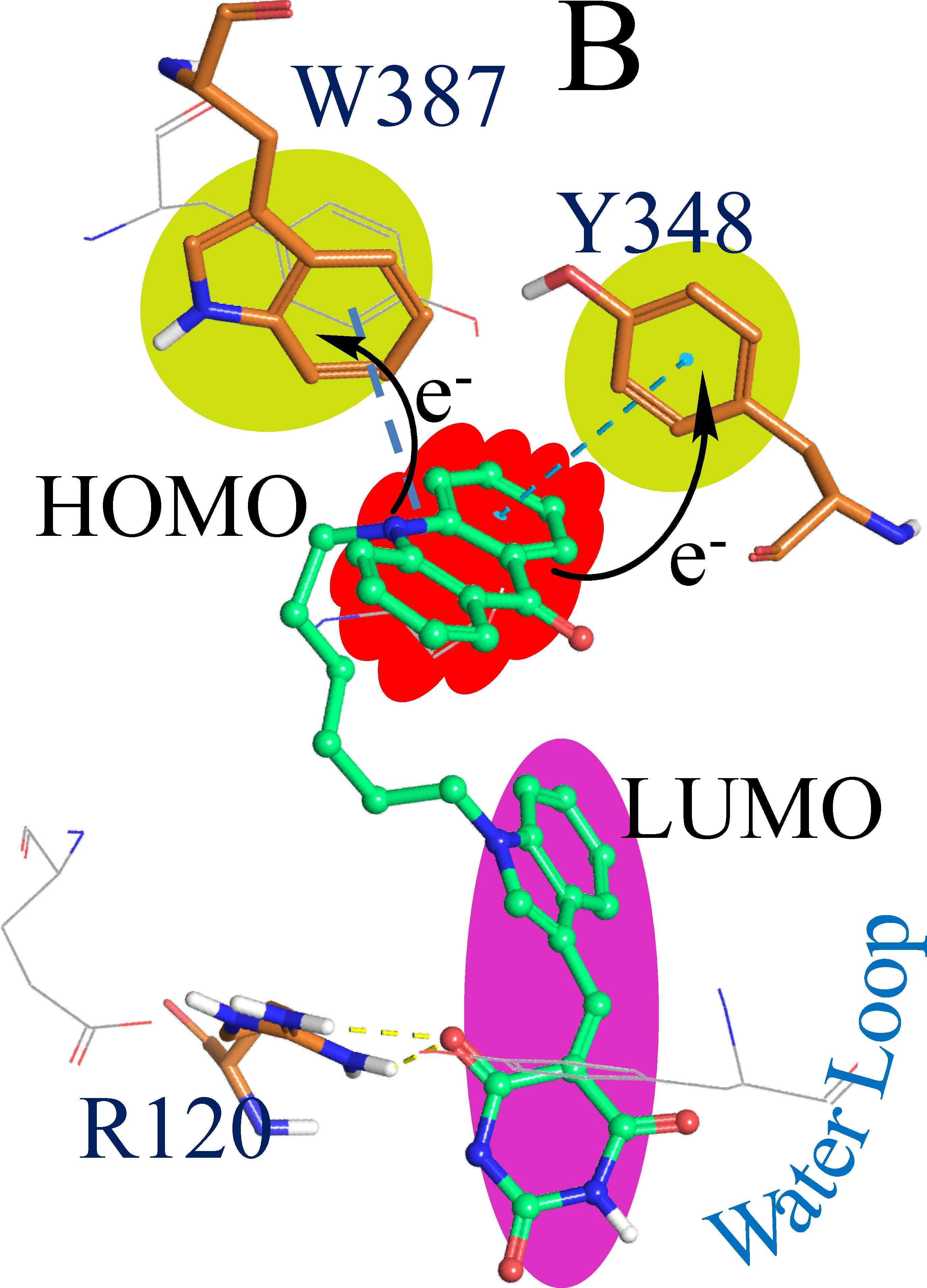
**

**Figure S25**. (A) HOMOs and LUMOs for compound **1** at the B3LYP level of calculation. (Isosurface = 0.05 a.u.) and (B) fluorescence quenching of compound **1** by COX-2.

**UV-Vis and fluorescence studies**

In order to support in-silico experiments, the interactions of **1**, **13d** and **15d** with COX-2 were checked with UV-vis and fluorescence spectral techniques. The UV-Vis spectrum of **1** at 1 µM concentration in Tris-HCl buffer (pH 7.25) exhibited absorption bands at 255 and 420 nm. Incremental addition of COX-2 to the solution of **1** resulted in the absorbance decrease at 255 and 420 nm indicating interactions of the compound with COX-2. The appearance of level-off in the visible region (500-700 nm) was attributed to the Mie scattering due to the formation of aggregates. The fluorescence spectrum of 0.5 µM solution of **1** in Tris-HCl buffer (pH 7.25) exhibited emission band at 567 nm when excited at 420 nm. Upon addition of COX-2 to the aqueous solution of the compound, there was significant quenching in fluorescence emission. Corroborating the results of molecular modelling studies, the changes in the UV-Vis spectra as well as the fluorescence spectrum of the compound on addition of COX-2 probably occurred due to the HOMO-LUMO interactions between **1** and the enzyme (Figure S27). The linear Stern-Volmer plot of decrease in fluorescence intensity at 567 nm on increasing COX-2 concentration gave Stern-Volmer constant Ksv 3.30x10^4^ M^-1^. The detection limit of the compound for COX-2 was 0.02 nM (Figure 27C, 27D).


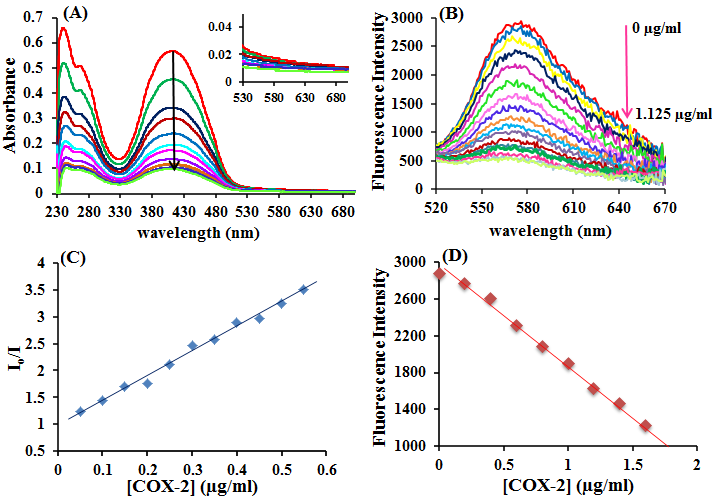


**Figure S26**. (A) Changes in the UV-Vis spectrum of compound **1** (1 µM, red trace) on incremental addition of COX-2. Inset: levelling off tail on addition of COX-2. (B) Emission spectra of compound **1** (0.5 µM) upon addition of increasing concentrations of COX-2 (0.05-1.125 µg/mL) in Tris-HCl buffer (0.1 mM, pH 7.25). (C) Stern-Volmer plot for the fluorescence quenching of compound **1** by COX-2 in Tris-HCl buffer (0.1 mM, pH 7.25). (D) Linear plot of change in fluorescence intensity of compound **1** at 567 nm Vs [COX-2] for determination of detection limit of the compound.

**
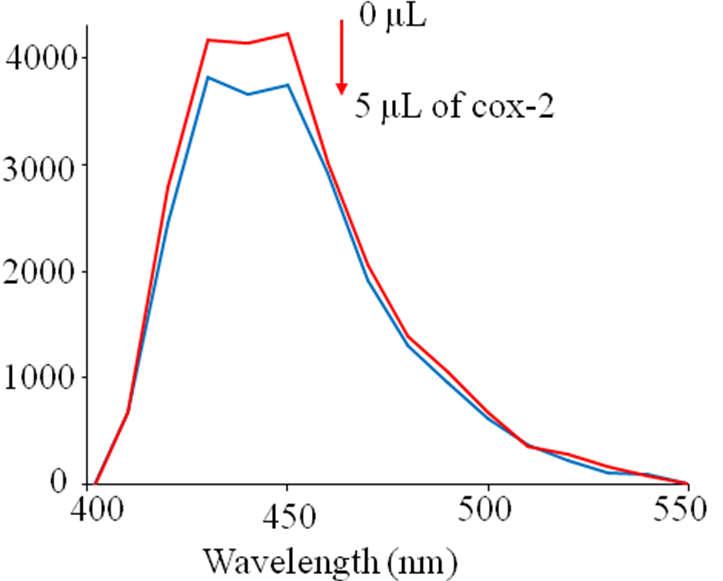
**

**Figure S27**. Small decrease in the fluorescence intensity of compound **13d** (red trace) (1 μM, DMSO-H_2_O, 1:9 v/v) on addition of even 5 μL of COX-2 (in Tris-HCl buffer, 0.1 mM, pH 7.25) (blue band). Excitation at 360 nm and emission at 450 nm.

**
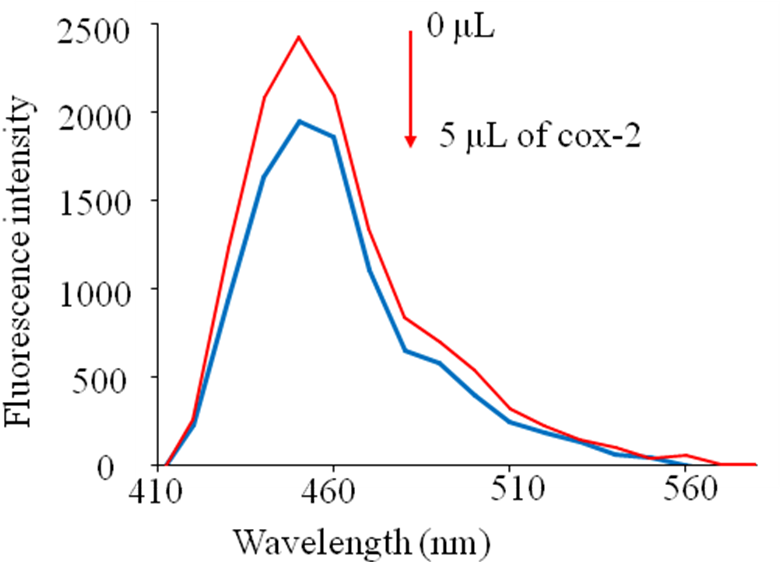
**

**Figure S28**. Small decrease in the fluorescence intensity of compound **15d** (red trace) (1 μM, DMSO-H_2_O, 1:9 v/v) on addition of even 5 μL of COX-2 (in Tris-HCl buffer, 0.1 mM, pH 7.25) (blue band) (blue band). Excitation at 360 nm and emission at 450 nm.

| **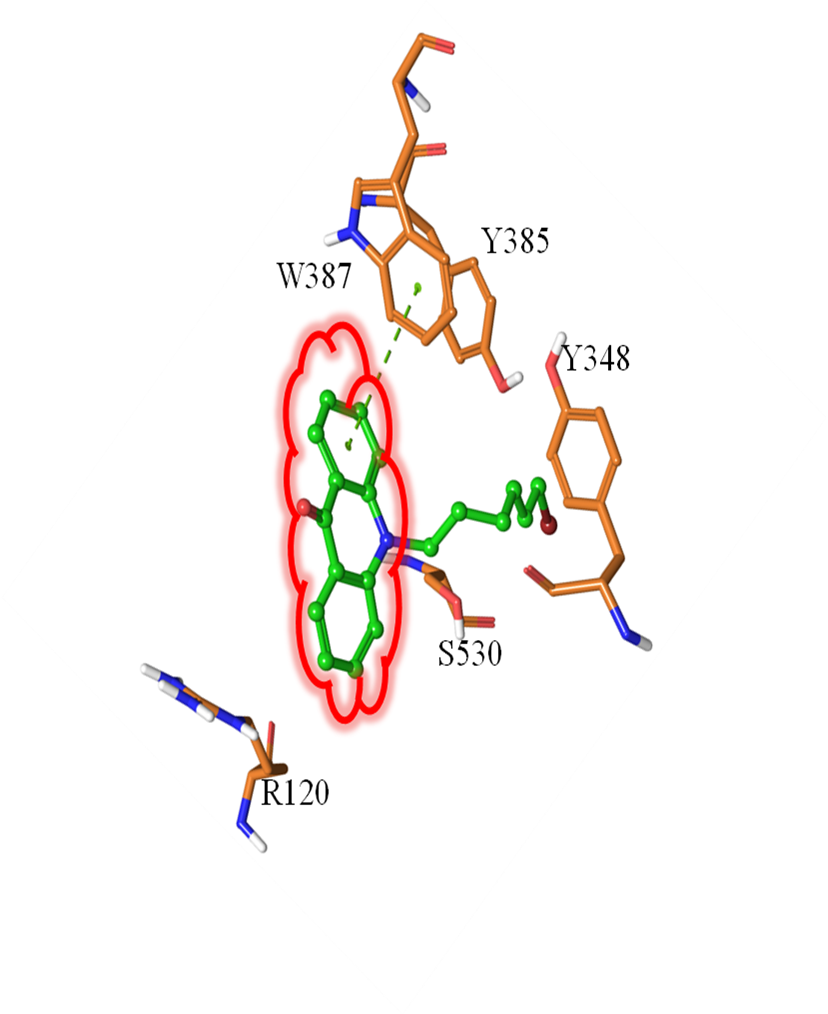** | 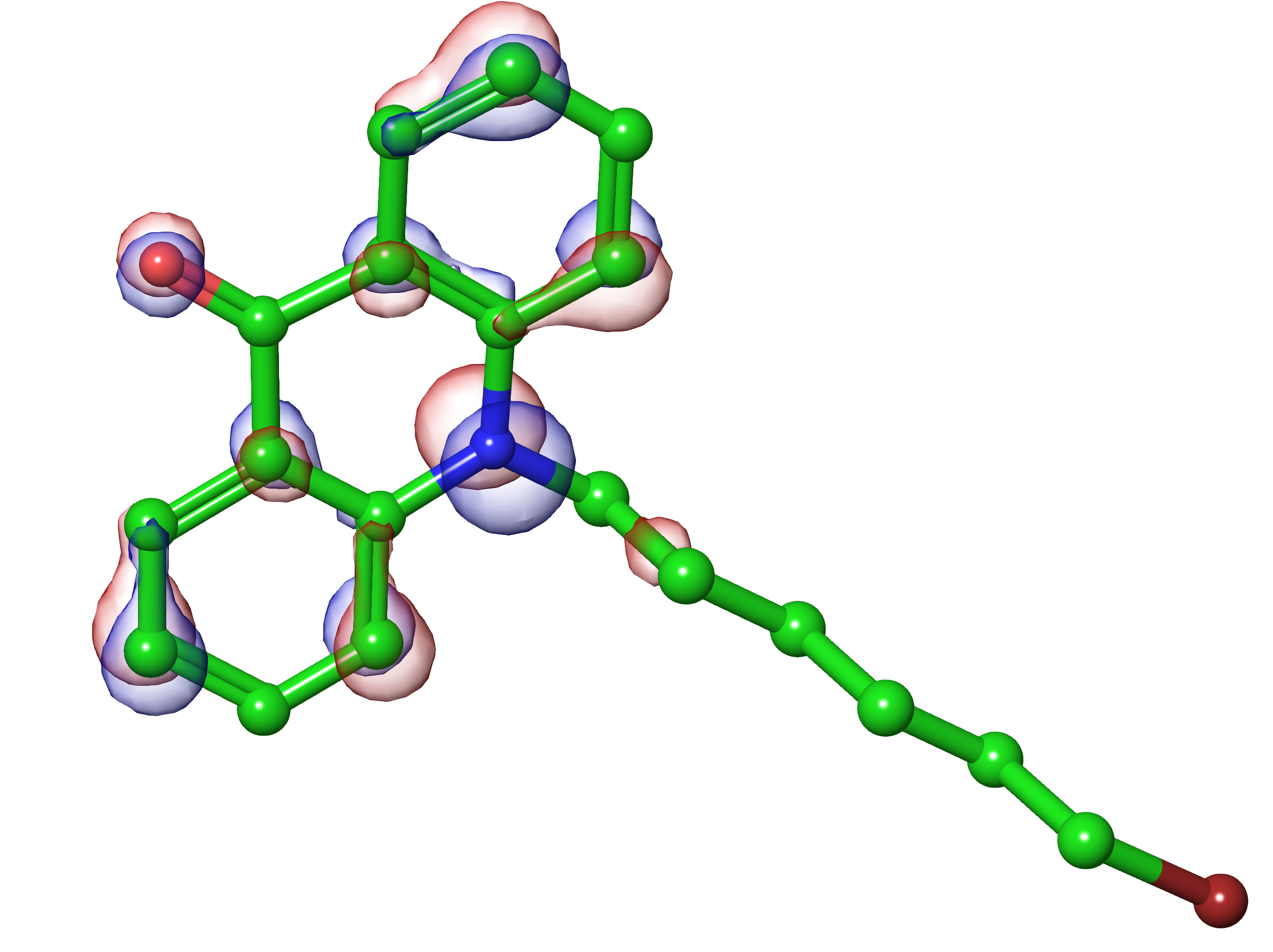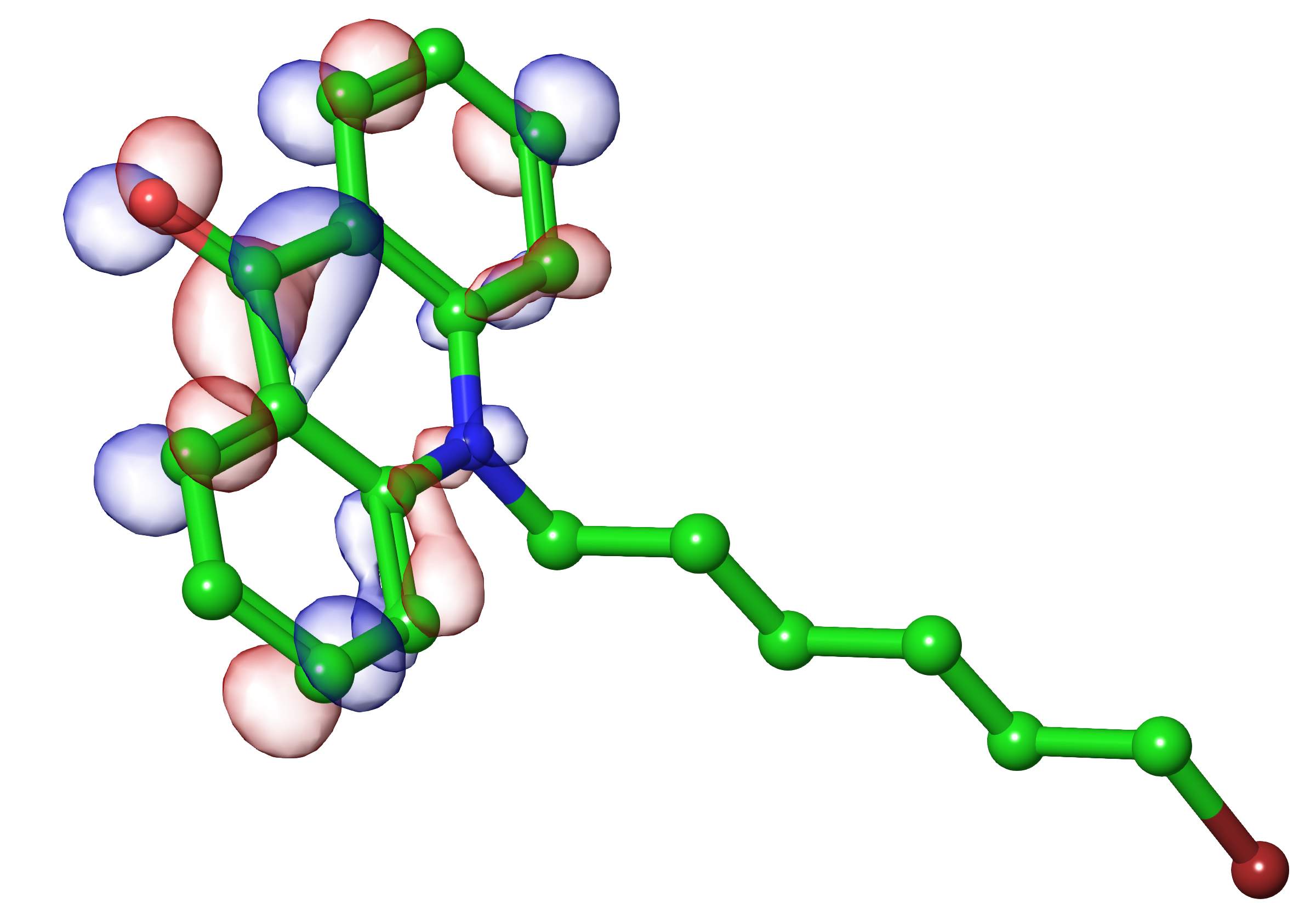 |
| --- | --- |

**Figure S29**. HOMO-LUMO of **13d** interacting with COX-2. Both the HOMO and LUMO were present on the acridone moiety.

| **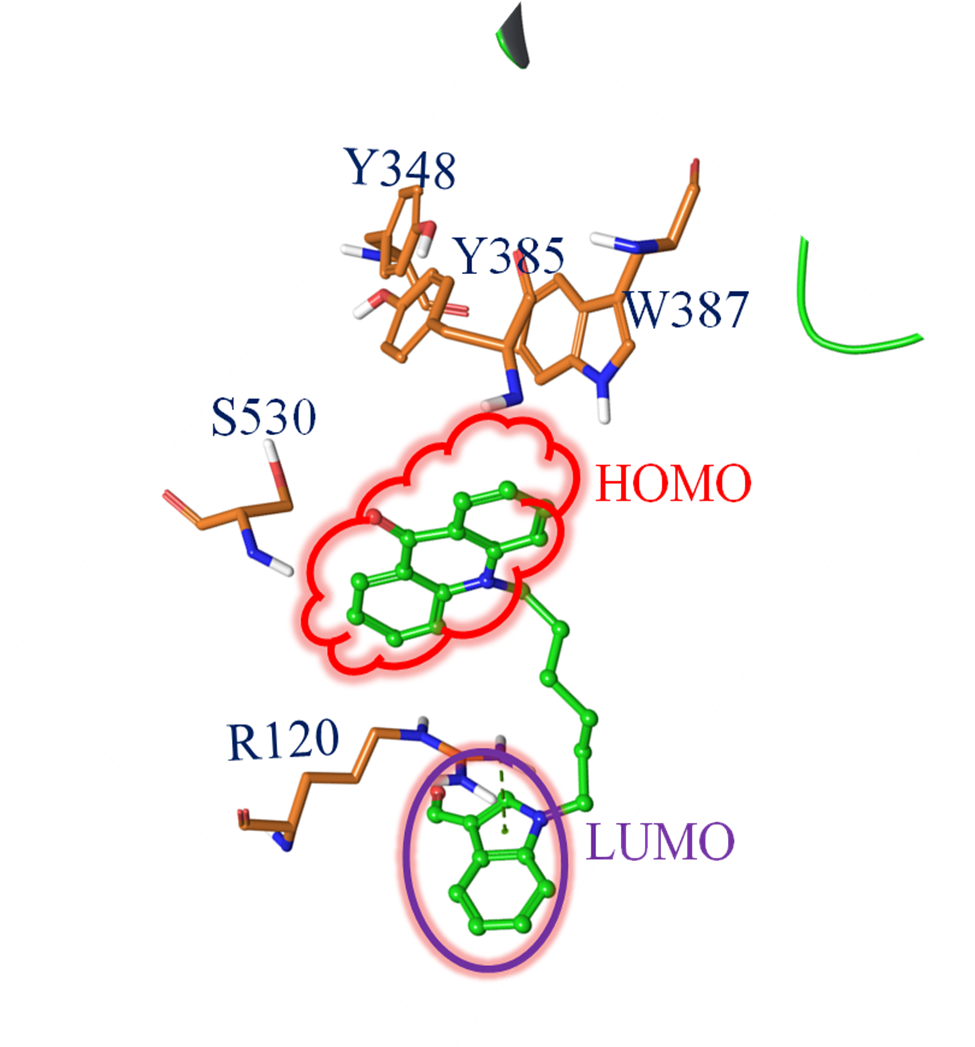** | **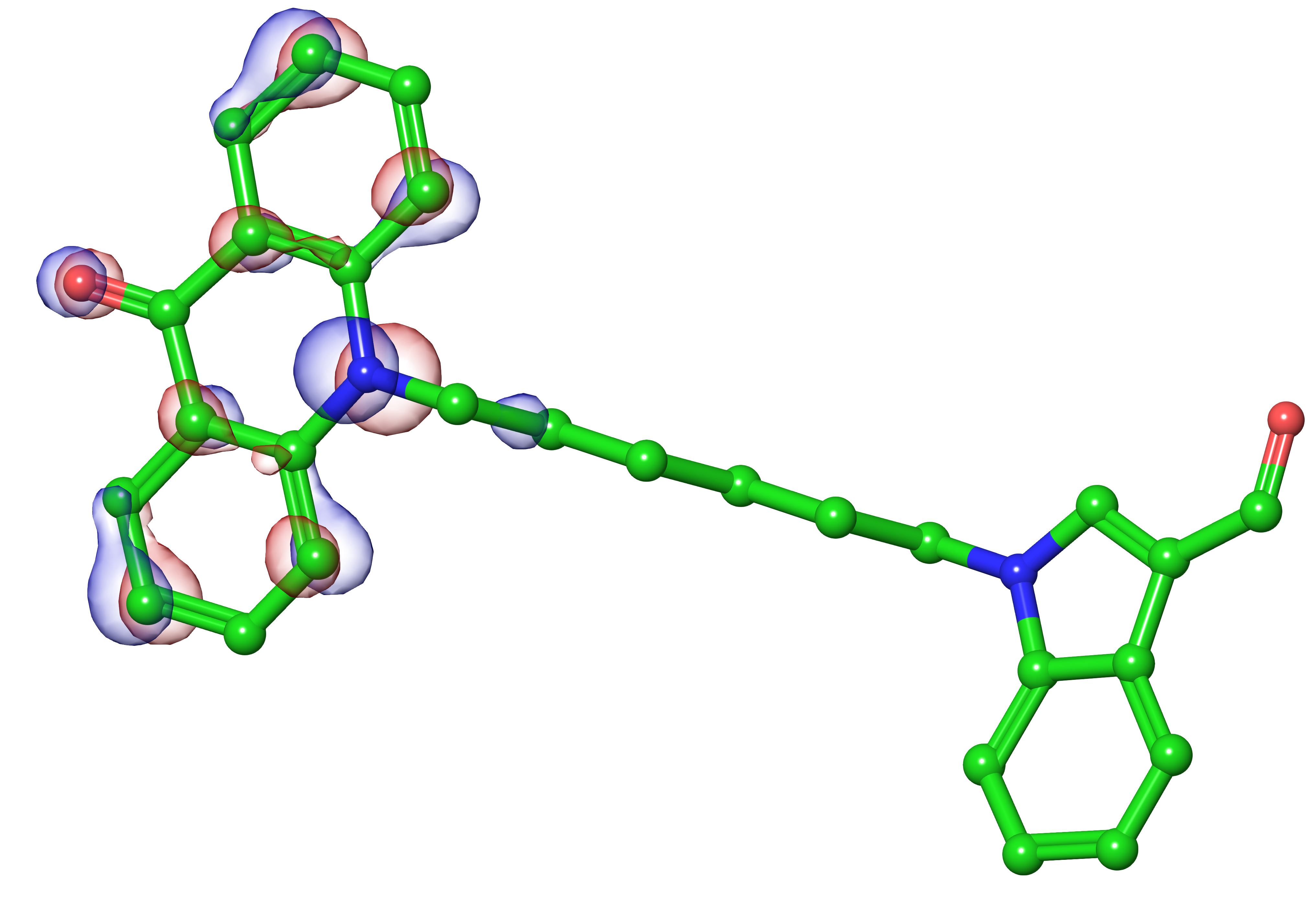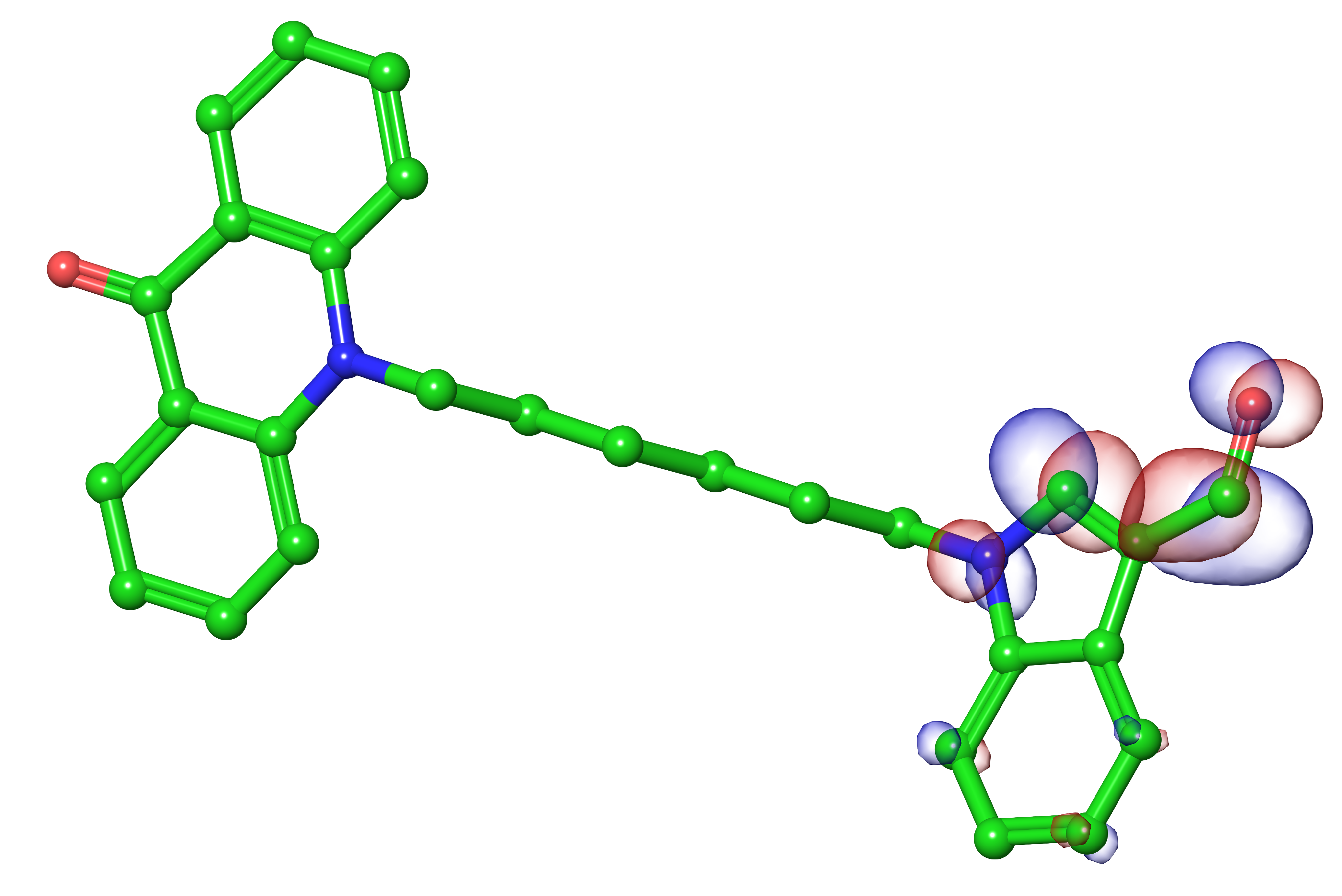** |
| --- | --- |

**Figure S30**. HOMO-LUMO of **15d** interacting with COX-2. The indole moiety was placed away from the water loop.

**
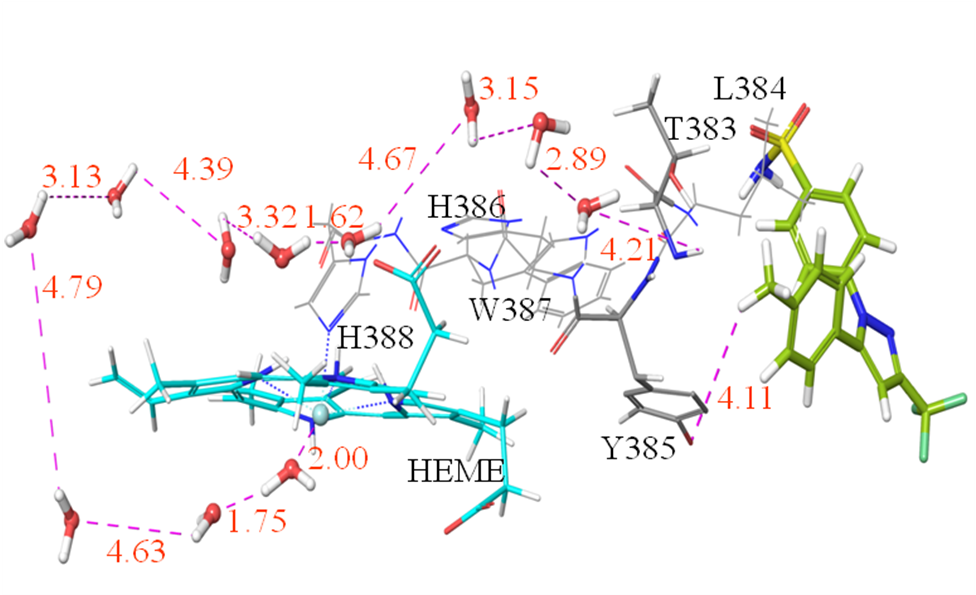
**

**Figure S31**. MD of COX-2 – AA – heme – celecoxib complex over 50 ns showing placement of celecoxib in the AA binding site and no disturbance to the water loop.

**
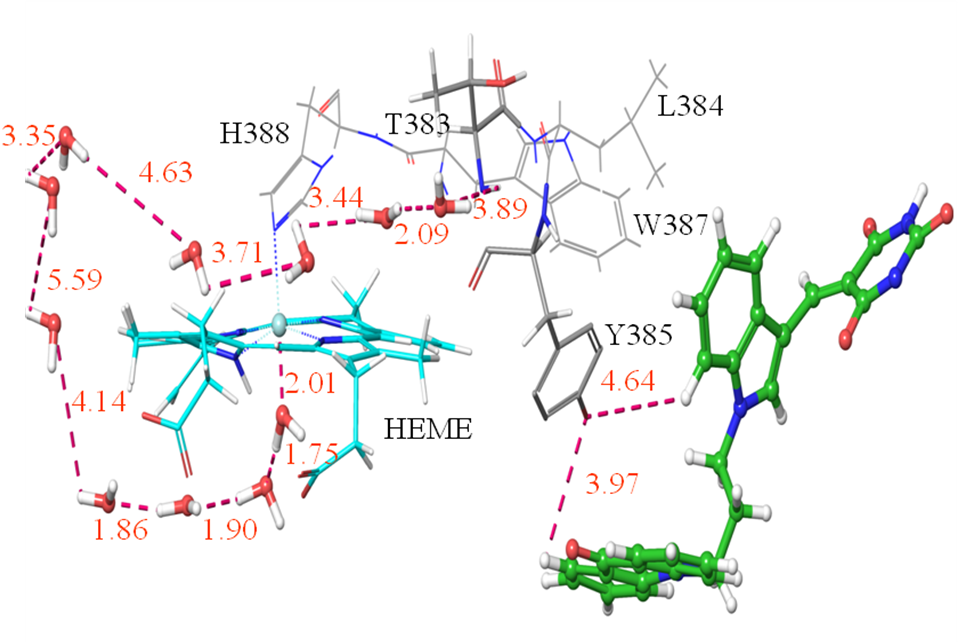
**

**Figure 32**. MD of COX-2 – AA – heme – compound **3** complex over 50 ns showing the placement of compound **3** in the AA binding site and no disturbance to the water loop.

**Synthesis of the compounds**

**General note**. Melting points were determined in capillaries and are uncorrected, ^1^H and ^13^C NMR spectra were recorded on JEOL 400 MHz, 100MHz and Bruker 500MHz and 125 MHz NMR spectrometer, respectively using CDCl_3_ and DMSO- *d_6_* as solvents. Chemical shifts are given in ppm with TMS as an internal reference. *J*  values are given in Hertz. Mass Spectra were recorded on Bruker micrOTOF QII Mass spectrometer. IR spectra were recorded on Agilent Technologies Cary 630 FTIR Spectrometer. The reactions under microwave conditions were performed in a microwave synthesizer (BIOTAGE INITIATOR EXP – RU) at 90 W and 120 ^o^C. Reactions were monitored by thin layer chromatography (TLC) on glass plates coated with silica gel GF-254. Column Chromatography was performed with 60- 120 mesh silica.

**Synthesis of Acridin-9(10*H*)-one (12**)

|  | 2-Chlorobenzoic acid (1 mmol) was dissolved in *iso*-amyl alcohol (50 mL) and treated with aniline (1 mmol) in the presence of K_2_CO_3_ (1.5 mmol) and |
| --- | --- |

CuO (0.25 mmol) by refluxing at 150 ^o^C for 36h. After the completion of the reaction, iso-amyl alcohol was evaporated under vacuum on rotary evaporator and hot water was added to the reaction mixture. Precipitates obtained on acidification were filtered via suction filteration and then dried in oven at 60 ^o^C. The precipitates were then dissolved in 10 mL of H_2_SO_4_ and heated at 100 ^o^C for 4h. The hot mixture was poured into cold water and the light yellow colored precipitates obtained were filtered and washed three times with water followed by acetone to get the pure product **12**. Light yellow solid; yield 48%, mp >300 ^o^C. ^1^H NMR (500 MHz, DMSO-*d_6_*) δ: 7.22-7.25 (m, 2H, ArH), 7.52 (d, *J* = 8.4 Hz, 2H, ArH), 7.69-7.73 (m, 2H, ArH), 8.21 (d, *J* = 8.05 Hz, 2H, ArH), 11.71 (s, NH); ^13^C NMR (125 MHz, normal/DEPT- 135) δ: 117.7 (+ve, ArCH), 120.9 (ArC), 121.4 (+ve, ArCH), 126.4 (+ve, ArCH), 133.9 (+ve, ArCH), 141.3 (ArC), 177.2 (C=O).

**General procedure for the synthesis of compounds 13 (a-d) (A).** Compound **12** (1 mmol) was dissolved in dimethylformamide (15 mL) and poured into the reaction vessel containning NaH (2 mmol, washed with dry hexane) at 0 ^o^C for the generation of anion followed by the addition of dibromopropane/dibromobutane/dibromopentane/dibromohexane (1.5 mmol). The reaction was continued for 4h at 40 ^o^C and monitored via TLC. After the completion of reaction; it was quenched by adding cold water (15 mL) and the reaction mixture was extracted using ethyl acetate (4 x 25 mL). The combined organic part was dried over anhydrous Na_2_SO_4_ and evaporated under vacuum and crude products **13(a-d)** were obtained which were then purified with column chromatography by using ethyl acetate and hexane as eluents.

**10-(2-Bromopropyl)acridin-9(10*H*)-one (13a).**

|  | Compound **13a** was synthesized by general procedure A using **12** and 1,3- dibromopropane. Light yellow crystalline solid, yield 40%, mp 98-100 ^o^C. ^1^H NMR (500 MHz, CDCl_3_) δ: 2.46-2.51 (m, 2H, CH_2_), 3.64 (t, *J* = 5.9 |
| --- | --- |

Hz, 2H, CH_2_), 4.58 (t, *J* = 8 Hz, 2H, CH_2_), 7.29-7.32 (m, 2H, ArH), 7.57 (d, *J* = 8.7Hz, 2H, ArH), 7.73-7.76 (m, 2H, ArH), 8.58-8.59 (dd, *J* = 7.9 Hz, 1.6 Hz, 2H, ArH); ^13^C NMR (125 MHz, normal/DEPT-135) δ: 29.5 (-ve, CH_2_), 30.1 (-ve, CH_2_), 44.5 (-ve, CH_2_), 114.2 (+ve, ArCH), 121.4 (+ve, ArCH), 122.6 (ArC), 128.1 (+ve, ArCH), 134.1 (+ve, ArCH), 141.7 (ArC), 177.9 (C=O).

**10-(4-Bromobutyl)acridin-9(10*H*)-one (13b).**

|  | Compound **13b** was synthesized by general procedure A using **12** and 1,4- dibromobutane. Light yellow crystalline solid, yield 60%, mp 149-150 ^o^C. ^1^H NMR (500 MHz, CDCl_3_) δ: 2.11-2.14 (m, 4H, 2 x CH_2_), 3.53 (t, *J* = 5.9 Hz, 2H, CH_2_), 4.39 (t, *J* = 7.5 Hz, 2H, CH_2_), 7.28-7.31 (m, 2H, ArH), |
| --- | --- |

7.50 (d, *J* = 8.7 Hz, 2H, ArH), 7.72-7.75 (m, 2H, ArH), 8.57-8.59 (dd, *J* = 8Hz, 1.6 Hz, 2H, ArH); ^13^C NMR (125 MHz, normal/DEPT-135) δ: 25.7 (-ve, CH_2_), 29.7 (-ve, CH_2_), 32.5 (-ve, CH_2_), 45.1 (-ve, CH_2_), 114.3 (+ve, ArCH), 121.3 (+ve, ArCH), 122.5 (ArC), 128.1 (+ve, ArCH), 133.9 (+ve, ArCH), 141.6 (ArC), 177.9 (C=O).

**10-(5-Bromopentyl)acridin-9(10*H*)-one (13c).**

|  | Compound **13c** was synthesized by general procedure A using **12** and 1,5- dibromopentane. Light yellow crystalline solid, yield 60%, mp 117-118 ^o^C. ^1^H NMR (400 MHz, CDCl_3_) δ: 1.70-1.78 (m, 2H, CH_2_), 1.94-2.05 (m, 4H, 2 x |
| --- | --- |

CH_2_), 3.48 (t, *J* = 6.4 Hz, 2H, CH_2_), 4.37 (t, *J* = 8 Hz, 2H, CH_2_), 7.29-7.33 (m, 2H, ArH), 7.49 (d, *J* = 8.4 Hz, 2H, ArH), 7.72-7.77 (m, 2H, ArH), 8.58-8.60 (dd, *J* = 8 Hz, 1.6 Hz, 2H, ArH); ^13^C NMR (100 MHz, normal/DEPT-135) δ: 25.4 (-ve, CH_2_), 26.4 (-ve, CH_2_), 32.1 (-ve, CH_2_), 33.3 (-ve, CH_2_), 45.9 (-ve, CH_2_), 114.3 (+ve, ArCH), 121.3 (+ve, ArCH), 122.5 (ArC), 128.1 (+ve, ArCH), 134.0 (+ve, ArCH), 141.7 (ArC), 178.0 (C=O).

**10-(6-Bromohexyl)acridin-9(10*H*)-one (13d).**

|  | Compound **13d** was synthesized by general procedure **A** using **12** and 1,6- dibromohexane. Light yellow crystalline solid, yield 62%, mp 107-108 ^o^C; ^1^H NMR (500 MHz, CDCl_3_) δ: 1.58-1.60 (m, 4H, 2 x CH_2_), 1.90-1.97 (m, 4H, 2 x |
| --- | --- |

CH_2_), 3.44 (t, *J* = 6.6 Hz, 2H, CH_2_), 4.33 (t, *J* = 8.2 Hz, 2H, CH_2_), 7.26-7.30 (m, 2H, ArH), 7.47 (d, *J* = 8.7 Hz, 2H, ArH), 7.70-7.73 (m, 2H, ArH), 8.56-8.58 (dd, *J* = 8Hz, 1.2 Hz, 2H, ArH); ^13^C NMR (125 MHz, normal/DEPT-135) δ: 26.1 (-ve, CH_2_), 27.0 (-ve, CH_2_), 27.8 (-ve, CH_2_), 32.5 (-ve, CH_2_), 33.4 (-ve, CH_2_), 45.9 (-ve, CH_2_). 114.4 (+ve, ArH), 121.2 (+ve, ArH), 122.5 (ArC), 128.0 (+ve, ArCH), 133.8 (+ve, ArCH), 141.7 (ArC), 177.9 (C=O).

**General procedure for the synthesis of compounds 15(a-d) (B)**. Compounds **13** (1 mmol) were treated with indole-3-carboxaldehyde (1 mmol) in the presence of NaH (1.5 mmol, after washing with dry hexane) in acetonitrile (15 mL). The progress of the reaction was monitored by TLC and after completion of the reaction, it was quenched by adding cold water. A creamish white solid was separated which was filtered via suction filteration. The solid obtained was further dissolved in chloroform and dried over anhydrous Na_2_SO_4_ and chloroform was distilled off to get the pure product.

**1-(3-(9-Oxoacridin-10(9*H*)-yl)propyl)-1*H*-indole-3-carbaldehyde (15a).**

|  | Compound **15a** was synthesized by the general procedure **B** by reacting **13a** with indole-3-carboxaldehyde. Creamish white solid; yield 65%; mp 172-174 ^o^C. ^1^H NMR (500 MHz, CDCl_3_) δ: 2.53-2.58 (m, 2H, CH_2_), 4.33 (t, *J* = 8.1 Hz, 2H, CH_2_), 4.46 (t, *J* = 5.6 Hz, 2H, CH_2_), 7.03-7.05 (d, *J*= 8.65 Hz, |
| --- | --- |

2H, ArH), 7.24-7.28 (m, 2H, ArH), 7.39-7.41 (m, 3H, ArH), 7.53 (t, *J* = 7.7 Hz, 2H, ArH), 7.82 (s, 1H, ArH), 8.40-8.42 (m, 1H, ArH), 8.53-8.55 (d, *J* = 7.9 Hz, 2H, ArH), 10.07 (s, CHO); ^13^C NMR (125 MHz, normal/DEPT-135) δ: 27.1 (-ve, CH_2_), 42.7 (-ve, CH_2_), 44.5 (-ve, CH_2_), 109.8 (+ve, ArCH), 113.6 (+ve, ArCH), 118.7 (ArC), 121.5 (+ve, ArCH), 122.5 (+ve, ArCH), 123.4 (+ve, ArCH), 124.5 (+ve, ArCH), 125.6 (ArC), 128.2 (+ve, ArCH), 134.0 (+ve, ArCH), 137.0 (ArC), 137.7 (+ve, ArCH), 141.3 (ArC), 177.7 (C=O), 184.4 (C=O).

**1-(4-(9-Oxoacridin-10(9*H*)-yl)butyl)-1*H*-indole-3-carbaldehyde (15b).**

|  | Compound **15b** was synthesized by the general procedure **B** by reacting **13b** with indole-3-carboxaldehyde. Creamish white solid; yield 70%; mp 168-169 ^o^C. ^1^H NMR (400 MHz, CDCl_3_) δ: 1.93-2.00 (m, 2H, CH_2_), 2.11-2.18 (m, 2H, CH_2_), 4.26-4.34 (m, 4H, CH_2_), 7.25-7.40 (m, 7H, ArH), 7.63-7.68 (m, 3H, ArH), 8.31-8.33 (m, 1H, ArH), 8.54-8.56 (dd, *J* = 8 Hz, 1.6 Hz, 2H, ArH), |
| --- | --- |

9.96 (s, 1H, CHO); ^13^C NMR (100 MHz, normal/DEPT-135) δ: 24.7 (-ve, CH_2_), 27.2 (-ve, CH_2_), 45.2 (-ve, CH_2_), 46.8 (-ve, CH_2_), 109.9 (+ve, ArCH), 114.2 (+ve, ArCH), 118.4 (ArC), 121.5 (+ve, ArCH), 122.4 (+ve, ArCH), 122.5 (ArC), 123.2 (+ve, ArCH), 124.3 (+ve, ArCH), 125.5 (ArC), 128.2 (+ve, ArCH), 134.1 (+ve, ArCH), 137.0 (ArC), 137.9 (+ve, ArCH), 141.5 (ArC), 177.9 (C=O), 184.6 (+ve, CHO).

**1-(5-(9-Oxoacridin-10(9*H*)-yl)pentyl)-1*H*-indole-3-carbaldehyde (15c).**

|  | Compound **15c** was synthesized by the general procedure **B** by reacting **13c** with indole-3-carboxaldehyde. Creamish white solid; yield 77%; mp 163-164 ^o^C. ^1^H NMR (400 MHz, CDCl_3_) δ: 1.51-1.57 (m, 2H, CH_2_), 1.89-2.05 (m, 4H, CH_2_), 4.21-4.30 (m, 4H, CH_2_), 7.24-7.29 (m, 3H, ArH), 7.32-7.39 (m, 4H, ArH), 7.66-7.70 (m, 3H, ArH), 8.30-8.33 (m, 1H, ArH), 8.55-8.57 (dd, *J* |
| --- | --- |

= 8 Hz, 1.6 Hz, 2H, ArH), 10.00 (s, 1H, CHO); ^13^C NMR (100 MHz, normal/DEPT-135) δ: 24.3 (-ve, CH_2_), 26.9 (-ve, CH_2_), 29.7 (-ve, CH_2_), 45.6 (-ve, CH_2_), 47.1 (-ve, CH_2_), 110.0 (+ve, ArCH), 114.3 (+ve, ArCH), 118.3 (ArC), 121.4 (+ve, ArCH), 122.3 (+ve, ArCH), 122.5 (ArC), 123.1 (ArC), 124.2 (+ve, ArCH), 125.5 (ArC), 128.2 (+ve, ArCH), 134.0 (+ve, ArCH), 137.1 (ArC), 138.1 (+ve, ArCH), 141.6 (ArC), 178.0 (C=O), 184.6 (CHO).

**1-(6-(9-Oxoacridin-10(9*H*)-yl)hexyl)-1*H*-indole-3-carbaldehyde (15d).**

|  | Compound **15d** was synthesized by the general procedure **B** by reacting **13d** with indole-3-carboxaldehyde. Creamish white solid, yield 60%, mp 159-160 ^o^C, ^1^H NMR (500 MHz, CDCl_3_) δ: 1.46-1.51 (m, 2H, CH_2_), 1.53-1.59 (m, 2H, CH_2_), 1.87-2.00 (m, 4H, 2 x CH_2_), 4.19 (t, *J* = 7 Hz, 2H, CH_2_), 4.27 (t, *J* = 8.2 Hz, 2H, CH_2_), 7.28-7.30 (m, 3H, ArH), 7.33-7.39 (m, 3H, ArH), |
| --- | --- |

7.41 (d, *J* = 8.7 Hz, 2H, ArH), 7.68-7.71 (m, 2H, ArH), 8.32-8.33 (m, 1H, ArH), 8.57-8.59 (dd, *J* = 7.9 Hz, 1.4 Hz, 2H, ArH), 10.0 (s, 1H, CHO); ^13^C NMR (125 MHz, normal/DEPT-135 NMR) δ: 26.5 (-ve, CH_2_), 26.8 (-ve, CH_2_), 27.1 (-ve, CH_2_), 29.7 (-ve, CH_2_), 45.7 (-ve, CH_2_), 47.1 (-ve, CH_2_), 109.9 (+ve, ArCH), 114.3 (+ve, ArCH), 118.1 (ArC), 121.2 (+ve, ArCH), 122.1 (+ve, ArCH), 122.5 (ArC), 122.9 (+ve, ArCH), 123.9 (+ve, ArCH), 128.0 (+ve, ArCH), 133.9 (+ve, ArCH), 137.1 (ArC), 137.9 (+ve, ArCH), 141.6 (ArC), 177.9 (C=O), 184.3 (C=O).

**General procedure for the synthesis of compounds 1-4 (C)**. An equivalent mixture of aldehyde **15(a-d)** (1 mmol) and active methylene compound– barbituric acid/1, 3-dimethyl barbituric acid (1 mmol) was taken in round bottom flask and dissolved in methanol. The reaction mixture was then refluxed at 90 ^o^C for 6 h. The yellow solid separated was filtered via suction filtration followed by washings with methanol and dried in air to get the pure products.

**5-((1-(3-(9-Oxoacridin-10(9*H*)-yl)propyl)-1*H*-indol-3-yl)methylene)pyrimidine-2,4,6 (1*H*, 3*H*, 5*H*)-trione (3)**.

Compound **3** was synthesized by the general procedure **C** by the reaction of **15a** and barbituric acid. Yellow solid; yield 80%; mp >300 ^o^C; IR (ATR, cm^-1^): 3145, 2832, 1729, 1550, 1110, 745; ^1^H NMR (500 MHz, DMSO-*d_6_*) δ: 2.38-2.40 (m, 2H, CH_2_), 4.55 (t, *J* = 8.1 Hz, CH_2_), 4.77 (t, *J* = 6.6 Hz, CH_2_), 7.31-7.32 (m, 2H, ArH), 7.38-7.43 (m, 2H, ArH), 7.75 (s, 3H, ArH), 7.87-7.88 (d, *J* = 7.1 Hz, 1H, ArH), 7.93-7.95 (d, *J* = 7.9 Hz, 2H, ArH), 8.70 (s, 1H, ArH), 9.68 (s, 1H, ArH), 11.07 (s, NH), 11.15 (s, NH); 13C NMR (125 MHz, normal/DEPT-135) δ: 27.2 (-ve, CH_2_), 43.0 (-ve, CH_2_), 44.7 (-ve, CH_2_), 111.2 (ArC), 112.3 (+ve, ArCH), 115.9 (+ve, ArCH), 118.5 (+ve, ArCH), 121.7 (+ve, ArCH), 122.1 (ArC), 123.6 (+ve, ArCH), 124.3 (+ve, ArCH), 127.2 (+ve, ArCH), 130.2 (ArC), 134.7 (+ve, ArCH), 137.0 (ArC), 141.8 (ArC), 142.1 (+ve, ArCH), 143.4 (+ve, ArCH), 150.9 (ArC), 163.6 (C=O), 164.9 (C=O), 176.9 (C=O); HRMS (ESI) *m/z* for C_29_H_22_N_4_O_4_ [M+Na]^+^ calcd. 513.1533, found 513.1516.

| **5-((1-(5-(9-Oxoacridin-10(9*H*)-yl)pentyl)-1*H*-indol-3-yl)methylene)pyrimidine-2,4,6(1*H*, 3*H*, 5*H*)-trione (2).**   |  | Compound **2** was synthesized by the general procedure **C** by the reaction of **15c** and barbituric acid. Yellow solid; yield 82%; mp >300 ^o^C; IR (ATR, cm^-1^): 3369, 3049, 2937, 1722, 1543, 1118, 745; ^1^H NMR (400 MHz, DMSO*-d_6_*) δ: 1.46-1.54 (m, 2H, CH_2_), 1.76-1.78 (m, | | --- | --- |   2H, CH_2_), 1.89-1.93 (m, 2H, CH_2_), 4.38-4.40 (m, 4H, CH_2_), 7.25-7.29 (m, 2H, ArH), 7.31-7.35 (m, 2H, ArH), 7.66-7.77 (m, 5H, ArH), 7.85-7.87 (m, 1H, ArH), 8.27-8.30 (dd, *J* = 8.4Hz, 1.6 Hz, 2H, ArH), 8.63 (s, 1H, ArH), 9.49 (s, 1H, ArH), 11.03 (s, 1H, NH), 11.12 (s, 1H, NH). ^13^C NMR (normal/DEPT-135) δ: 23.8 (-ve, CH_2_), 26.9 (-ve, CH_2_), 29.6 (-ve, CH_2_), 45.5 (-ve, CH_2_), 47.6 (-ve, CH_2_), 108.9 (ArC), 111.1 (ArC), 112.4 (+ve, ArCH), 116.3 (+ve, ArCH), 118.4 (+ve, ArCH), 121.7 (+ve, ArCH), 121.9 (ArC), 123.6 (+ve, ArCH), 124.3 (+ve, ArCH), 127.2 (+ve, ArCH), 130.3 (ArC), 134.8 (+ve, ArCH), 137.0 (ArC), 141.8 (ArC), 142.5 (+ve, ArCH), 143.5 (+ve, ArCH), 151.0 (ArC), 163.7 (C=O), 165.1 (C=O), 177.0 (C=O); HRMS (ESI) *m/z* for C_31_H_26_N_4_O_4_ [M+Na]^+^ calcd. 541.1846, found 541.1836.  **5-((1-(6-(9-Oxoacridin-10(9*H*)-yl)hexyl)-1*H*-indol-3-yl)methylene)pyrimidine-2,4,6(1*H*, 3*H*, 5*H*)-trione (1).**   |  | Compound **1** was synthesized by the general procedure **C** by the reaction of **15d** and barbituric acid. Yellow solid; yield 80%; mp >300 ^o^C; IR (ATR, cm^-1^): 3183, 3093, 2929, 1707, 1550, 1051, 745; ^1^H NMR (500 MHz, DMSO-*d_6_*) δ: 1.39-1.44 (m, 2H, CH_2_), 1.53-1.59 (m, 2H, CH_2_), | | --- | --- |   1.73-1.79 (m, 2H, CH_2_), 1.85-1.91 (m, 2H, CH_2_), 4.43 (t, *J* = 7 Hz, 4H, 2 x CH_2_), 7.31-7.34 (m, 2H, ArH), 7.36-7.37 (m, 2H, ArH), 7.72-7.74 (m, 1H, ArH), 7.77-7.82 (m, 4H, ArH), 7.89-7.91 (m, 1H, ArH), 8.33-8.35 (m, 2H, ArH),8.67 (s, 1H, ArH), 9.55 (s, 1H, ArH), 11.04 (s, 1H, NH), 11.13 (s, 1H, NH); ^13^C NMR (125 MHz, normal/DEPT-135) δ: 25.8 (-ve, CH_2_), 26.3 (-ve, CH_2_), 27.1 (-ve, CH_2_), 29.6 (-ve, CH_2_), 45.5 (-ve, CH_2_), 47.4 (-ve, CH_2_), 108.9 (ArC), 111.0 (ArC), 112.2 (+ve, ArCH), 116.2 (+ve, ArCH), 118.4 (+ve, ArCH), 121.6 (+ve, ArCH), 122.0 (ArC), 123.5 (+ve, ArCH), 124.2 (+ve, ArCH), 127.2 (+ve, ArCH), 130.2 (ArC), 134.7 (+ve, ArCH), 136.9 (ArC), 141.8 (ArC), 142.3 (+ve, ArCH), 143.4 (+ve, ArCH), 150.9 (ArC), 163.9 (C=O), 164.9 (C=O), 176.8 (C=O); HRMS (ESI) *m/z* for C_32_H_28_N_4_O_4_ [M+Na]^+^ calcd. 555.2002, found 555.2055.  **1,3-Dimethyl-5-((1-(6-(9-oxoacridin-10(9H)-yl)hexyl)-1H-indol-3-yl)methylene)pyrimidi- ne-2,4,6(1*H*,3*H*,5*H*)-trione (4).**   |  | Compound **4** was synthesized by the general procedure **C** by the reaction of **15d** and 1,3-dimethyl barbituric acid. Yellow solid, yield 90%, mp 240-241 ^o^C; IR (ATR, cm^-1^): 3748, 2049, 2937, 1632, 1334, 745; ^1^H NMR (400 MHz, CDCl_3_) δ: 1.50-1.57 (m, 4H, CH_2_), 1.88-1.92 (m, 2H, CH_2_), 1.99-2.04 (m, 2H, CH_2_), 3.37 (s, 3H, CH_3_), 3.41 (s, 3H, CH_3_), 4.29 (t, *J* = 7.2 Hz, 4H, CH_2_), 7.24-7.27 (m, 2H, ArH), 7.35-7.41 | | --- | --- |   (m, 5H, ArH), 7.64-7.68 (m, 2H, ArH), 7.98-7.99 (m, 1H, ArH), 8.54-8.56 (m, 2H, ArH), 8.98 (s, 1H, ArH), 9.56 (s, 1H, ArH); ^13^C NMR (125 MHz, normal/DEPT-135) δ: 26.5 (-ve, CH_2_), 26.8 (-ve, CH_2_), 27.2 (-ve, CH_2_), 28.1 (+ve, CH_3_), 28.9 (+ve, CH_3_), 29.7 (-ve, CH_2_), 45.8 (-ve, CH_2_), 47.8 (-ve, CH_2_), 108.2 (ArC), 110.8 (+ve, ArCH), 112.1 (ArC), 114.4 (+ve, ArCH), 119.0 (+ve, ArCH), 121.3 (+ve, ArCH), 122.5 (ArC), 123.4 (+ve, ArCH), 124.2 (+ve, ArCH), 128.1 (+ve, ArCH), 130.7 (ArC), 133.9 (+ve, ArCH), 136.6 (ArC), 141.7 (ArC), 142.2 (+ve, ArCH), 146.3 (+ve, ArCH), 151.8 (ArC), 162.3 (C=O), 163.9 (C=O), 178.0 (C=O); HRMS (ESI) *m/z* for C_34_H_32_N_4_O_4_ [M+H]^+^ calcd. 561.2496, found 561.2458. |
| --- | --- | --- | --- | --- | --- | --- |

**(*Z*)-3-((1-methyl-1*H*-indol-3-yl)methylene)indolin-2-one (11)**. Compound **11** was procured by subjecting an equivalent mixture of N-methylindole-3-carboxalehyde (1 mmol) and oxindole (1 mmol) in chloroform (2 mL) using catalytic amount of piperidine, to microwave radiations at 120 ^o^C for 20 min. The reaction was monitored by TLC after every 5 min. The orange solid separated was filtered and washed 2-3 times with diethylether and dried in air. Orange solid, yield 60%, mp 209-210 ^o^C; ^1^H NMR (500 MHz, DMSO-*d_6_*) δ: 3.90 (s, 3H, CH_3_), 6.80 (d, *J* = 7.6 Hz, 1H, ArH), 6.92-6.96 (m, 1H, ArH), 7.07-7.11 (m, 1H, ArH), 7.21-7.29 (m, 2H, ArH), 7.53-7.55 (m, 1H, ArH), 7.83 (d, *J* = 8 Hz, 1H, ArH), 8.07 (s, 1H, ArH), 8.14-8.16 (m, 1H, ArH), 9.35 (s, 1H, ArH), 10.48 (s, NH).

**General procedure for the synthesis of compounds 5-7 (D).** For the synthesis of compounds **5-7**, compound **11** (1 mmol) was made to react with compounds **13(a-d)** (1.2 mmol) in the presence of NaH (1.5 mmol, after washing with dry hexane) in acetonitrile (15 mL) at 70 ^o^C for 24h. After the completion of reaction, the reaction was quenched by adding ice-cold water (10 mL). The reaction mixture was filtered and in the filterate part yellow colored precipitates started separating which were further filtered followed by washing with ether to get the pure products.

**(Z)-10-(3-(3-((1-methyl-1H-indol-3-yl)methylene)-2-oxoindolin-1-yl)propyl)acridin-9(10H)-one (7).**

|  | Compound **7** was synthesized by general procedure **D** using compound **11** and **13a**. Yellow solid, yield 70%, mp 229-230 ^o^C; IR (ATR, cm^-1^): 2937, 1688, 1595, 1263, 935, 674; ^1^H NMR (500 MHz, CDCl_3_:DMSO-*d*_6_, 9:1) δ: 2.43 (br, 2H, CH_2_), 2.61 (s, DMSO), 3.93 (s, 3H, CH_3_), 4.20 (t, *J* = 7.2 Hz, 2H, CH_2_), 4.49 (t, *J* |
| --- | --- |

= 7.2 Hz, 2H, CH_2_), 6.90 (d, *J* = 7.1 Hz, 1H, ArH), 7.15-7.18 (m, 1H, ArH), 7.24-7.26 (m, 3H, ArH), 7.34-7.35 (m, 4H, ArH), 7.40 (m, 1H, ArH), 7.57 (t, *J* = 7.1 Hz, 2H, ArH), 7.74 (d, *J* = 7Hz, 1H, ArH), 7.95 (br, 1H, ArH), 8.08 (s, 1H, ArH), 8.54 (d, *J* = 7.3 Hz, 2H, ArH), 9.48 (s, 1H, ArH); ^13^C NMR (125 MHz, normal/DEPT-135) δ: 25.5 (-ve, CH_2_), 33.7 (-ve, CH_3_), 37.0 (-ve, CH_2_), 41.0 (+ve, DMSO), 43.6 (-ve, CH_2_), 107.8 (+ve, ArCH), 110.2 (+ve, ArCH), 111.0 (ArC), 114.2 (+ve, ArCH), 117.4 (ArC), 118.0 (+ve, ArCH), 118.1 (+ve, ArCH), 121.3 (+ve, ArCH), 121.6 (+ve, ArCH), 121.9 (+ve, ArCH), 122.5 (ArC), 123.0 (+ve, ArCH), 125.3 (ArC), 126.9 (+ve, ArCH), 127.7 (+ve, ArCH), 128.0 (+ve, ArCH), 129.1 (ArC), 134.0 (+ve, ArCH), 136.9 (ArC), 137.7 (+ve, ArCH), 139.2 (ArC), 141.5 (ArC), 167.6 (C=O), 177.9 (C=O); HRMS (ESI) *m/z* for C_34_H_27_N_3_O_2_ [M+H]^+^ calcd. 510.2176, found 510.2208.

**(*Z*)-10-(5-(3-((1-Methyl-1*H*-indol-3-yl)methylene)-2-oxoindolin-1-yl)pentyl)acridin-9(10*H*)-one (2).**

|  | Compound **2** was synthesized by general procedure **D** using **11** and **13c**. Yellow solid, yield 75%, mp 259-260 ^o^C; IR (ATR, cm^-1^): 2918, 1681, 1599, 1379, 1121, 670; ^1^H NMR (500 MHz, DMSO-*d*_6_) δ: 1.60-1.61 (m, 2H, CH_2_), 1.78-1.81 (m, 2H, CH_2_), 1.88-1.89 (m, 2H,CH_2_), 3.87-3.99 (m, 5H, CH_2_, CH_3_), 4.46 (t, *J* = 8.2 Hz, |
| --- | --- |

2H, CH_2_), 7.02-7.11 (m, 2H, ArH), 7.21-7.24 (m, 1H, ArH), 7.25-7.34 (m, 4H, ArH), 7.59-7.63 (m, 1H, ArH), 7.79-7.85 (m, 4H, ArH), 7.95-7.97 (m, 1H, ArH), 8.20-8.24 (m, 2H, ArH), 8.35 (d, *J* = 7.8 Hz, 2H, ArH), 9.47 (s, 1H, ArH); HRMS (ESI) *m/z* for C_36_H_31_N_3_O_2_ [M+H]^+^ calcd. 538.2489, found 538.2092.

**(*Z*)-10-(6-(3-((1-Methyl-1*H*-indol-3-yl)methylene)-2-oxoindolin-1-yl)hexyl)acridin-9(10H)-one (5).**

|  | Compound **5** was synthesized by general procedure **D** using **11** and **13d**. Yellow solid, yield 70%, mp 264-265 ^o^C; IR (ATR, cm^-1^): 2929, 1684, 1595, 1375, 920, 670; ^1^H NMR (400 MHz, DMSO-*d_6_*) δ: 1.40-1.45 (m, 2H, CH_2_), 1.52-1.79 (m, 6H, 3 x |
| --- | --- |

CH_2_), 3.81 (t, *J* = 6.8 Hz, 2H, CH_2_), 3.88 (s, 3H, CH_3_), 4.41 (t, *J* = 8 Hz, 2H, CH_2_), 6.99-7.03 (m, 2H, ArH), 7.15-7.29 (m, 6H, ArH), 7.54 (d, *J* = 8Hz, 1H, ArH), 7.76 (d, *J* = 3.2 Hz, 3H, ArH), 7.89-7.91 (m, 1H, ArH), 8.14-8.18 (m, 2H, ArH), 8.30 (d, *J* = 8 Hz, 2H, ArH), 9.42 (s, 1H, ArH). ^13^C NMR (100 MHz, normal/DEPT-135) δ: 26.1 (-ve, CH_2_), 26.7 (-ve, CH_2_), 27.2 (-ve, CH_2_), 27.8 (-ve, CH_2_), 33.9 (+ve, CH_3_), 39.5 (-ve, CH_2_), 45.7 (-ve, CH_2_), 108.5 (+ve, ArCH), 110.9 (ArC), 111.2 (+ve, ArCH), 116.3 (+ve, ArCH), 118.1 (ArC), 119.1 (+ve, ArCH), 121.5 (+ve, ArCH), 121.6 (+ve, ArCH), 121.7 (+ve, ArCH), 122.0 (ArC), 123.2 (+ve, ArCH), 125.1 (ArC), 127.2 (+ve, ArCH), 127.5 (+ve, ArCH), 129.2 (ArC), 134.7 (+ve, ArCH), 137.0 (ArC), 137.7 (+ve, ArCH), 140.1 (ArC), 141.9 (ArC), 166.6 (C=O), 176.9 (C=O); HRMS (ESI) *m/z* for C_37_H_33_N_3_O_2_ [M+H]^+^ calcd. 552.2645, found 552.2757.

**(Z)-3-(3,4,5-trimethoxybenzylidene)indolin-2-one (14).**

|  | The OH group of syringaldehyde (1 mmol) was protected by its reaction with CH_3_I (1.5 mmol) in the presence of K_2_CO_3_ (2 mmol) in DMF (10 ml) at 40 ^o^C for 6h. The reaction was quenched by |
| --- | --- |

adding ice followed by evaporating DMF and the protected syringaldehyde was extracted by ethyl acetate (4 x 25 mL) and then dried over anhydrous Na_2_SO_4_ followed by evaporation of ethyl acetate under vacuum to get the product. For the synthesis of **14**, equimolar quantities of protected syringaldehyde (1 mmol) and oxindole (1 mmol) were taken in the reaction vessel and were dissolved in methanol. The reaction mixture was refluxed at 90 ^o^C for 3 h and the yellow colored solid separated was filtered via suction filteration followed by washing with diethyl ether to get pure product **14**. Orange solid, yield 40%, mp 155-156 ^o^C; ^1^H NMR (500 MHz, CDCl_3_) δ: 3.94 (s, 3H, OCH_3_), 3.97 (s, 6H, OCH_3_), 6.83 (d, *J* = 7.7 Hz, 1H, ArH), 7.03-7.06 (m, 1H, ArH), 7.20-7.24 (m, 1H, ArH), 7.47 (s, 1H, ArH), 7.52 (d, *J* = 7.5 Hz, 1H, ArH), 7.82 (s, 2H, ArH), 8.22 (br, 1H, NH). ^13^C NMR (125 MHz, normal/DEPT-135) δ: 56.2 (+ve, OCH_3_), 60.9 (+ve, OCH_3_), 109.3 (+ve, ArCH), 110.0 (+ve, ArCH), 119.0 (+ve, ArCH), 121.8 (+ve, ArCH), 125.1 (ArC), 125.6 (ArC), 128.6 (+ve, ArCH), 129.3 (ArC), 138.0 (+ve, ArCH), 139.2 (ArC), 140.7 (ArC), 152.7 (ArC), 167.9 (C=O).

**General procedure for the synthesis of compounds 16-18 (E)**. For the synthesis of compounds 1**6-18**, compounds **13(a-d)** (1 mmol) were made to react with **14** (1 mmol) in the presence of K_2_CO_3_ (1.5 mmol) in dimethylformamide (15 mL) at 60 ^o^C for 24h. After 24h, reaction was quenched by adding ice and DMF was evaporated under vacuum. The product was extracted by using ethyl acetate (4 x 25 mL) and the combined organic part was dried over anhydrous Na_2_SO_4_ and evaporated under vacuum on rotary evaporator. The crude product obtained was purified with column chromatography using ethyl acetate and hexane as eluents.

**(*Z*)-10-(3-(2-Oxo-3-(3,4,5-trimethoxybenzylidene)indolin-1-yl)propyl)acridin-9(10*H*)-one (16).**

|  | Compound **16** was synthesized by general procedure **E** using **14** and **13a.** Yellow solid, yield 40%, mp 179-180 ^o^C; ^1^H NMR (500 MHz, CDCl_3_) δ: 2.36-2.42 (m, 2H, CH_2_), 3.89-3.97 (m, 9H, 3x OCH_3_), 4.09-4.13 (m, 2H, CH_2_), 4.45-4.51 (m, 2H, CH_2_), 6.82-6.86 (m, 1H, |
| --- | --- |

ArH), 6.94 (s, 1H, ArH), 6.99 (t, *J* = 7.7 Hz, 1H, ArH), 7.23-7.25 (m, 1H, ArH), 7.27-7.30 (m, 2H, ArH), 7.34-7.39 (m, 2H, ArH), 7.55-7.60 (m, 1H, ArH), 7.60-7.64 (m, 2H, ArH), 7.85 (s, 1H, ArH), 7.90 (d, *J* = 7.6 Hz, 1H, ArH), 8.54-8.56 (m, 2H, ArH). ^13^C NMR (125 MHz, normal/DEPT-135) δ: 25.2 (-ve, CH_2_), 37.3 (-ve, CH_2_), 43.4 (-ve, CH_2_), 56.2 (+ve, OCH_3_), 61.1 (+ve, OCH_3_), 106.8 (+ve, ArCH), 108.0 (+ve, ArCH), 108.4 (+ve, ArCH), 110.0 (+ve, ArCH), 114.1 (+ve, ArCH), 119.1 (+ve, ArCH), 121.4 (+ve, ArCH), 122.1 (+ve, ArCH), 122.5 (ArC), 123.3 (+ve, ArCH), 124.2 (ArC), 124.8 (ArC), 125.8 (ArC), 128.1 (+ve, ArCH), 128.8 (+ve, ArCH), 129.2 (ArC), 129.9 (+ve, ArCH), 134.0 (+ve, ArCH), 138.3 (+ve, ArCH), 139.5 (ArC), 140.4 (ArC), 141.5 (ArC), 142.7 (ArC), 152.7 (ArC), 153.3 (ArC), 168.9 (C=O), 177.9 (C=O).

**(*Z*)-10-(5-(2-Oxo-3-(3,4,5-trimethoxybenzylidene)indolin-1-yl)pentyl)acridin-9(10*H*)-one (17)**

|  | Compound **17** was synthesized by general procedure **E** using **14** and **13c**. Yellow solid, yield 68%, mp 194-195 ^o^C; ^1^H NMR (400 MHz, CDCl_3_) δ: 1.23-1.27 (m, 2H, CH_2_), 1.85-1.88 (m, 2H, CH_2_), 1.96-2.05 (m, 2H, CH_2_), 3.87-3.95 (m, 11H, 3 x OCH_3_, CH_2_), 4.33-4.37 (m, 2H, CH_2_), |
| --- | --- |

6.82-6.94 (m, 4H, ArH), 7.27-7.30 (m, 1H, ArH), 7.46-7.56 (m, 4H, ArH), 7.66-7.75 (m, 2H, ArH), 7.80-7.84 (m, 2H, ArH), 8.56-8.58 (m, 2H, ArH); ^13^C NMR (100 MHz, normal/DEPT-135) δ: 26.9 (-ve, CH_2_), 27.5 (-ve, CH_2_), 29.7 (-ve, CH_2_), 39.5 (-ve, CH_2_), 46.0 (-ve, CH_2_), 56.4 (+ve, OCH_3_), 61.2 (+ve, OCH_3_), 106.7 (+ve, ArCH), 108.0 (+ve, ArCH), 110.0 (+ve, ArCH), 114.5 (+ve, ArCH), 118.9 (+ve, ArCH), 121.3 (+ve, ArCH), 121.8 (+ve, ArCH), 122.5 (ArC), 123.1 (+ve, ArCH), 125.1 (ArC), 128.1 (+ve, ArCH), 129.8 (+ve, ArCH), 134.0 (+ve, ArCH), 137.7 (+ve, ArCH), 141.7 (ArC), 152.8 (ArC), 153.3 (ArC), 177.4 (C=O), 178.0 (C=O).

**(*Z*)-10-(6-(2-Oxo-3-(3,4,5-trimethoxybenzylidene)indolin-1-yl)hexyl)acridin-9(10*H*)-one (18**).

|  | Compound **18** was synthesized by general procedure **E** using **14** and **13d**. Yellow solid, yield 40%, mp 198-199 ^o^C ; ^1^H NMR (400 MHz, CDCl_3_) δ: 1.54-1.64 (m, 4H, 2x CH_2_), 1.75-1.82 (m, 2H, CH_2_), 1.90-1.98 (m, 2H, CH_2_), 3.85 (s, 6H, 2x OCH_3_), 3.91-3.94 (m, 5H, OCH_3_, CH_2_), 4.29-4.35 (m, 2H, |
| --- | --- |

CH_2_), 6.80-6.85 (m, 1H, ArH), 6.88-6.92 (m, 2H, ArH), 7.23-7.30 (m, 4H, ArH), 7.43-7.53 (m, 2H, ArH), 7.65-7.73 (m, 2H, ArH), 7.76-7.82 (m, 2H, ArH), 8.54-8.58 (m, 2H, ArH). ^13^C NMR (100 MHz, normal/DEPT-135) δ: 26.6 (-ve, CH_2_), 26.7 (-ve, CH_2_), 27.2 (-ve, CH_2_), 27.6 (-ve, CH_2_), 39.7 (-ve, CH_2_), 46.1 (-ve, CH_2_), 56.3 (+ve, OCH_3_), 61.1 (+ve, OCH_3_), 106.6 (+ve, ArCH), 108.0 (+ve, ArCH), 108.5 (+ve, ArCH), 110.0 (+ve, ArCH), 114.6 (+ve, ArCH), 118.9 (+ve, ArCH), 121.3 (+ve, ArCH), 121.6 (+ve, ArCH), 122.5 (ArC), 123.1 (+ve, ArCH), 124.8 (ArC), 126.4 (ArC), 128.1 (+ve, ArCH), 128.6 (+ve, ArCH), 129.7 (+ve, ArCH), 130.2 (ArC), 134.0 (+ve, ArCH), 137.5 (+ve, ArCH), 141.8 (ArC), 143.6 (ArC), 152.7 (ArC), 153.3 (ArC), 168.6 (C=O), 178.1 (C=O).

**General procedure for the synthesis of compounds 8-10 (F)**. To the solution of compounds **8-10** in dichloromethane, anhydrous AlCl_3_ (1.5 mmol) was added under N_2_ atmosphere at 25 ^o^C. The reaction mixture was stirred for 2h and after completion the reaction was quenched by adding cold water and the product was extracted using dichloromethane (4 x 25 mL) and the organic part was dried over anhydrous Na_2_SO_4_ and evaporated under vacuum to procure the product.

**(*Z*)-10-(3-(3-(4-Hydroxy-3,5-dimethoxybenzylidene)-2-oxoindolin-1-yl)propyl)acridin-9(10*H*)-one (10).**

|  | Compound **10** was synthesized by general procedure **F** using **16** and anhydrous AlCl_3_. Yellow solid, yield 82%, mp 229-230 ^o^C; IR (ATR, cm^-1^): 3507, 2926, 1669, 1595, 1334, 1121, 752; ^1^H NMR (500 MHz, CDCl_3_) δ: 2.38-2.41 (m, 2H, CH_2_), 3.93-4.01 (m, 6H, |
| --- | --- |

2 x OCH_3_), 4.10-4.15 (m, 2H, CH_2_), 4.46-4.51 (m, 2H, CH_2_), 5.88 (s, 1H, OH), 6.83-6.87 (m, 1H, ArH), 6.97-7.00 (m, 2H, ArH), 7.24-7.26 (m, 2H, ArH), 7.27-7.28 (m, 2H, ArH), 7.35-7.39 (m, 2H, ArH), 7.55-7.58 (m, 1H, ArH), 7.60-7.63 (m, 2H, ArH), 7.85 (s, 1H, ArH), 7.93-7.95 (m, 1H, ArH), 8.54-8.57 (m, 2H, ArH). ^13^C NMR (125 MHz, normal/DEPT-135) δ: 25.2 (-ve, CH_2_), 37.3 (-ve, CH_2_), 43.4 (-ve, CH_2_), 56.4 (-ve, CH_2_), 106.8 (+ve, ArCH), 107.9 (+ve, ArCH), 108.4 (+ve, ArCH), 110.1 (+ve, ArCH), 114.1 (+ve, ArCH), 118.8 (+ve, ArCH), 121.4 (+ve, ArCH), 121.6 (ArC), 122.0 (+ve, ArCH), 122.1 (+ve, ArCH), 122.5 (ArC), 122.9 (+ve, ArCH), 124.8 (ArC), 125.7 (ArC), 128.1 (+ve, ArCH), 128.3 (+ve, ArCH), 129.6 (+ve, ArCH), 134.0 (+ve, ArCH), 136.7 (ArC), 138.8 (+ve, ArCH), 140.2 (ArC), 141.5 (ArC), 142.6 (ArC), 146.6 (ArC), 147.1 (ArC), 169.0 (C=O), 177.9 (C=O); HRMS (ESI) *m/z* for C_33_H_28_N_2_O_5_ [M+Na]^+^calcd. 555.1890, found 555.1943.

**(*Z*)-10-(5-(3-(4-Hydroxy-3,5-dimethoxybenzylidene)-2-oxoindolin-1-yl)pentyl)acridin-9(10*H*)-one (9).**

|  | Compound **9** was synthesized by general procedure **F** using **17** and anhydrous AlCl_3_. Yellow solid, yield 82%, mp 238-239 ^o^C; IR (ATR, cm^-1^): 3198, 2929, 1677, 1595, 1371, 1103, 745; ^1^H NMR (500 MHz, CDCl_3_) δ: 1.25-1.33 (m, 2H, CH_2_), 1.86-1.89 (m, |
| --- | --- |

2H, CH_2_), 2.00-2.04 (m, 2H, CH_2_), 3.87-3.99 (m, 8H, CH_2_, 2 x OCH_3_), 4.33-4.36 (m, 2H, CH_2_), 5.89 (s, OH), 6.83-6.96 (m, 3H, ArH), 7.26-7.30 (m, 3H, ArH), 7.47-7.56 (m, 3H, ArH), 7.67-7.75 (m, 2H, ArH), 7.85-7.95 (m, 2H, ArH), 8.58 (d, *J* = 7.2 Hz, 2H, ArH); ^13^C NMR (125 MHz, normal/DEPT) δ: 24.2 (-ve, CH_2_), 26.8 (-ve, CH_2_), 27.4 (-ve, CH_2_), 39.6 (-ve, CH_2_), 46.0 (-ve, CH_2_), 56.4 (+ve, OCH_3_), 106.7 (+ve, ArCH), 108.4 (+ve, ArCH), 110.1 (+ve, ArCH), 114.5 (+ve, ArCH), 118.6 (+ve, ArCH), 121.2 (ArC), 121.5 (+ve, ArCH), 122.5 (ArC), 122.7 (+ve, ArCH), 125.3 (ArC), 125.9 (ArC), 128.0 (+ve, ArCH), 129.4 (+ve, ArCH), 133.9 (+ve, ArCH), 136.6 (ArC), 138.1 (+ve, ArCH), 141.7 (ArC), 143.3 (ArC), 146.6 (ArC), 147.0 (ArC), 168.8 (C=O), 178.0 (C=O); HRMS (ESI) *m/z* for C_35_H_32_N_2_O_5_ [M+H]^+^ calcd. 561.2383, found 561.2318.

**(*Z*)-10-(6-(3-(4-Hydroxy-3,5-dimethoxybenzylidene)-2-oxoindolin-1-yl)hexyl)acridin-9(10*H*)-one (8).**

|  | Compound **8** was synthesized by general procedure **F** using **18** and anhydrous AlCl_3_. Yellow solid, yield 83%, mp 244-245 ^o^C; IR (ATR, cm^-1^): 3369, 2929, 1669, 1599, 1338, 1129, 749; ^1^H NMR (400 MHz, CDCl_3_) δ: 1.54-1.62 (m, 4H, 2 x CH_2_), 1.75-1.82 (m, 2H, CH_2_), 1.98-1.97 (m, 2H, CH_2_), 3.81-3.85 (m, 2H, CH_2_), |
| --- | --- |

3.89-3.97 (m, 6H, 2x OCH_3_), 4.31-4.35 (m, 2H, CH_2_), 5.84 (s, 1H, OH), 6.80-6.92 (m, 3H, ArH), 7.22-7.30 (m, 3H, ArH), 7.43-7.52 (m, 3H, ArH), 7.65-7.73 (m, 2H, ArH), 7.76-7.84 (m, 1H, ArH), 7.92 (s, 1H, ArH), 8.56-8.58 (m, 2H, ArH). ^13^C NMR (100 MHz, normal/DEPT-135) δ: 26.6 (-ve, CH_2_), 26.7 (-ve, CH_2_), 27.2 (-ve, CH_2_), 27.6 (-ve, CH_2_), 39.7 (-ve, CH_2_), 46.1 (-ve, CH_2_), 56.5 (+ve, OCH_3_), 106.7 (+ve, ArCH), 108.0 (+ve, ArCH), 108.5 (+ve, ArCH), 110.0 (+ve, ArCH), 114.6 (+ve, ArCH), 118.6 (+ve, ArCH), 121.3 (+ve, ArCH), 121.5 (+ve, ArCH), 121.6 (+ve, ArCH), 122.5 (ArC), 122.8 (+ve, ArCH), 125.4 (ArC), 126.0 (ArC), 128.1 (+ve, ArCH), 129.4 (+ve, ArCH), 134.0 (+ve, ArCH), 136.5 (ArC), 138.0 (+ve, ArCH), 141.8 (ArC), 143.4 (ArC), 146.6 (ArC), 147.0 (ArC), 168.7 (C=O), 178.1 (ArC); HRMS (ESI) *m/z* for C_36_H_34_N_2_O_5_ [M+H]^+^ calcd. 575.2540, found 575.2193.

**NMR Spectra**


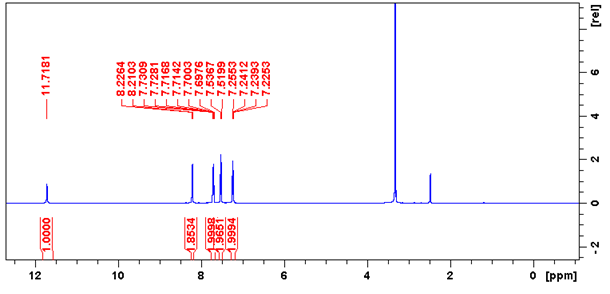


**Figure S33**. ^1^H NMR spectrum of compound **12** in DMSO-*d_6_*.


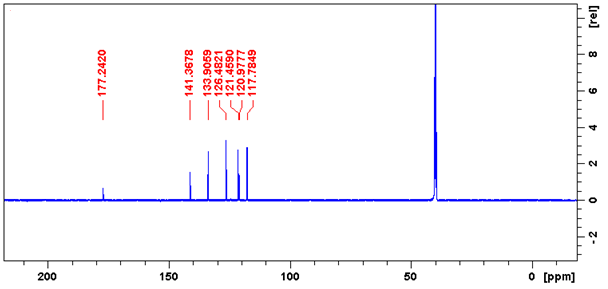


**Figure S34**. ^13^C NMR spectrum of compound **12** in DMSO-*d_6_*.


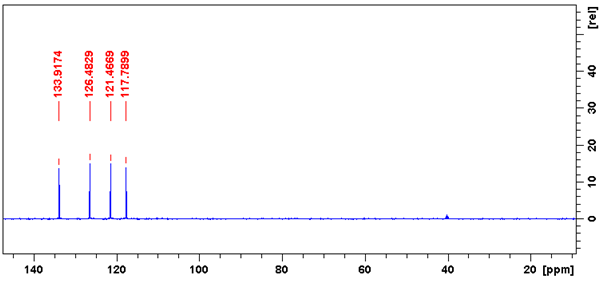


**Figure S35.** DEPT-135 spectrum of compound **12** in DMSO-*d*_6_.

**
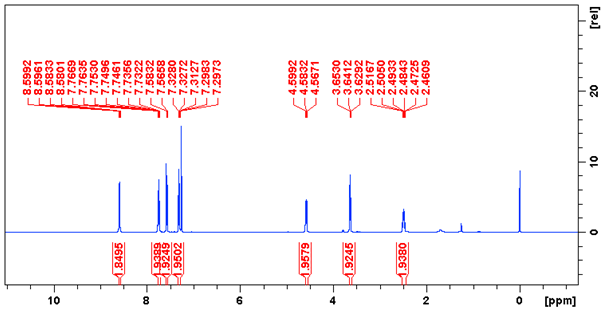
**

**Figure S36**. ^1^H NMR spectrum of compound **13a** in CDCl_3_.

**
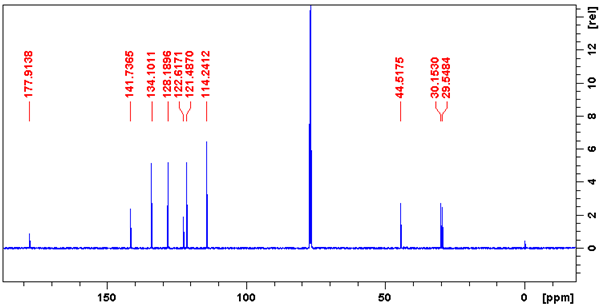
**

**Figure S37**. ^13^C NMR spectrum of compound **13a** in CDCl_3_.

**
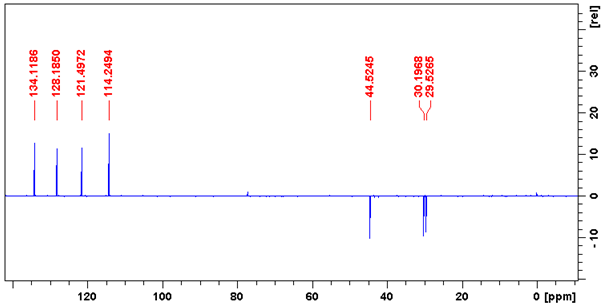
**

**Figure S38**. DEPT-135 spectrum of compound **13a** in CDCl_3_.

**
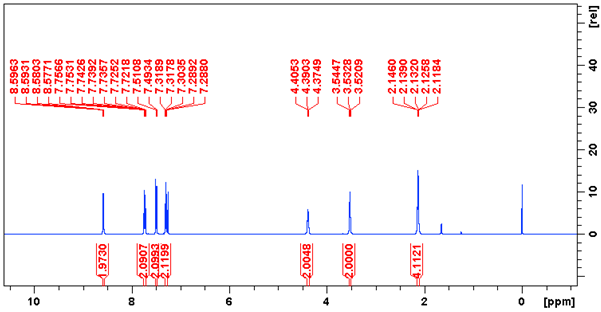
**

**Figure S39**. ^1^H NMR spectrum of compound **13b** in CDCl_3_.

**
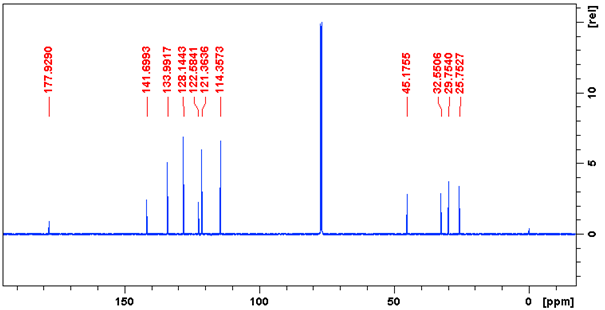
**

**Figure S40**. ^13^C NMR spectrum of compound **13b** in CDCl_3_.

**Figure S41**. DEPT-135 spectrum of compound **13b** in CDCl_3_.

**Figure S42**. ^1^H NMR spectrum of compound **13c** in CDCl_3_.

**Figure S43**. ^13^C NMR spectrum of compound **13c** in CDCl_3_.

**Figure S44**. DEPT-135 spectrum of compound **13c** in CDCl_3_.

**Figure S45**. ^1^H NMR spectrum of compound **13d** in CDCl_3_.

**Figure S46**. ^13^C NMR spectrum of compound **13d** in CDCl_3_.

**Figure S47**. DEPT-135 spectrum of compound **13d** in CDCl_3_.

**Figure S48**. ^1^H NMR spectrum of **15a** in CDCl_3_.

**Figure S49**. ^13^C NMR spectrum of **15a** in CDCl_3_.

**Figure S50**. DEPT-135 NMR spectrum of **15a** in CDCl_3_.

**Figure S51**. ^1^H NMR spectrum of **15b** in CDCl_3_.

**Figure S52**. ^13^C NMR spectrum of **15b** in CDCl_3_.

**Figure S53**. DEPT-135 NMR spectrum of **15b** in CDCl_3_.

**Figure S54**. ^1^H NMR spectrum of **15c** in CDCl_3_.

**Figure S55**. ^13^C NMR spectrum of **15c** in CDCl_3_.

**Figure S56**. DEPT-135 NMR spectrum of **15c** in CDCl_3_.

**Figure S57**. ^1^H NMR spectrum of **15d** in CDCl_3_.

**Figure S58**. Expansion of Aromatic region of ^1^H NMR spectrum of **15d** in CDCl_3_.

**Figure S59**. ^13^C NMR spectrum of **15d** in CDCl_3_.

**Figure S60**. DEPT-135 NMR spectrum of **15d** in CDCl_3_.

**Figure S61**. ^1^H NMR spectrum of **3** in DMSO-*d_6_*.

**Figure S62**. Expansion of Aromatic region of ^1^H NMR spectrum of **3** in DMSO-*d_6_*.

**Figure S63**. ^13^C NMR spectrum of **3** in DMSO-*d_6_*.

**Figure S64**. DEPT-135 NMR spectrum of **3** in DMSO-*d_6_.*

**Figure S65**. ^1^H NMR spectrum of **2** in DMSO-*d_6_*.

**Figure S66**. ^13^C NMR spectrum of **2** in DMSO-*d_6_*.

**Figure S67**. DEPT-135 NMR spectrum of **2** in DMSO-*d_6_.*

**Figure S68**. ^1^H NMR spectrum of **1** in DMSO-*d_6_*.

**Figure S69**. Expansion of Aromatic region of ^1^H NMR spectrum of **1** in DMSO-*d_6_*.

**Figure S70**. ^13^C NMR spectrum of **1** in DMSO-*d_6_*.

**Figure S71**. DEPT-135 NMR spectrum of **1** in DMSO-*d_6_.*

**Figure S72**. ^1^H NMR spectrum of **4** in CDCl_3_.

**Figure S73**. ^13^C NMR spectrum of **4** in CDCl_3_.

**Figure S74**. DEPT-135 NMR spectrum of **4** in CDCl_3_.

**Figure S75**. ^1^H NMR spectrum of compound **11** in DMSO-*d_6_*.

**Figure S76**. ^1^H NMR spectrum of compound **7** in CDCl_3_+DMSO-*d_6_* (9:1).

**Figure S77**. Expansion of aromatic region of ^1^H NMR spectrum of compound **7** in CDCl_3_+DMSO-*d*_6_ (9:1).

**Figure S78**. ^13^C NMR spectrum of compound **7** in CDCl_3_+DMSO-*d*_6_ (9:1).

**Figure S79**. DEPT-135 NMR spectrum of compound **7** in CDCl_3_+DMSO-*d*_6_ (9:1

**Figure S80**. ^1^H NMR spectrum of compound **6** in DMSO-*d*_6_.

**Figure S81**. Expansion of aromatic region of^1^H NMR spectrum of compound **6** in DMSO-*d*_6_.

**Figure S82**. ^1^H NMR spectrum of compound **5** in DMSO-*d*_6_.

**Figure S83**. ^13^C NMR spectrum of compound **5** in DMSO-*d*_6_.

**Figure S84**. DEPT-135 spectrum of compound **5** in DMSO-*d*_6_.

**Figure S85**. ^1^H NMR spectrum of compound **14** in CDCl_3_.

**Figure S86**. ^13^C NMR spectrum of compound **14** in CDCl_3_.

**Figure S87**. DEPT-135 spectrum of compound **14** in CDCl_3_.

**Figure S88**. ^1^H NMR spectrum of compound **16** in CDCl_3_.

**Figure S89**. Expansion of aromatic region of^1^H NMR spectrum of compound **16** in CDCl_3_.

**Figure S90**. ^13^C NMR spectrum of compound **16** in CDCl_3_.

**Figure S91**. DEPT-135 spectrum of compound **16** in CDCl_3_.

**Figure S92**. ^1^H NMR spectrum of compound **17** in CDCl_3_.

**Figure S93**. Expansion of aromatic region of ^1^H NMR spectrum of compound **17** in CDCl_3_.

**Figure S94**. ^13^C NMR spectrum of compound **17** in CDCl_3_.

**Figure S95**. DEPT-135 spectrum of compound **17** in CDCl_3_.

**Figure S96**. ^1^H NMR spectrum of compound **18** in CDCl_3_.

**Figure S97**. ^13^C NMR spectrum of compound **18** in CDCl_3_.

**Figure S98**. DEPT-135 spectrum of compound **18** in CDCl_3_.

**Figure S99**. ^1^H NMR spectrum of compound **19** in CDCl_3_.

**Figure S100**. ^13^C NMR spectrum of compound **19** in CDCl_3_.

**Figure S101**. DEPT-135 spectrum of compound **19** in CDCl_3_.

**Figure S102**. ^1^H NMR spectrum of **10** in CDCl_3_.

**Figure S103**. Expansion of aromatic region of ^1^H NMR spectrum of compound **10** in CDCl_3_.

**Figure S104**. ^13^C NMR spectrum of compound **10** in CDCl_3_.

**Figure S105**. DEPT-135 spectrum of compound **10** in CDCl_3_.

**Figure S106**. ^1^H NMR spectrum of **9** in CDCl_3_.

**Figure S107**. ^13^C NMR spectrum of compound **9** in CDCl_3_.

**Figure S108**. DEPT-135 spectrum of compound **9** in CDCl_3_.

**Figure S109**. ^1^H NMR spectrum of **8** in CDCl_3_.

**Figure S110**. ^13^C NMR spectrum of compound **8** in CDCl_3_.

**Figure S111**. DEPT-135 spectrum of compound **8** in CDCl_3_.

**Mass spectra of compounds**

**Figure S112**. HRMS of compound **3**C_29_H_22_N_4_O_4_ [M+Na]^+^ found 513.1516, calcd. 513.1533.

**Figure S113**. HRMS of compound **2** C_31_H_26_N_4_O_4_ [M+Na]^+^ found 541.1836, calcd. 541.1846.

**Figure S114**. HRMS of compound **1** C_32_H_28_N_4_O_4_ [M+Na]^+^ found 555.2055, calcd *m/z* 555.2002.

**Figure S115**. HRMS of compound **4** C_34_H_32_N_4_O_4_ [M+H]^+^ found 561.2458, calcd *m/z* 561.2496.

**Figure S116**. HRMS of compound **7** C_34_H_27_N_3_O_3_ [M+H]^+^ found 510.2208, calcd *m/z* 510.2176.

**Figure S117**. HRMS of compound **6** C_36_H_31_N_3_O_2_ [M+H]^+^ found 538.2092, calcd *m/z* 538.2489.

**Figure S118**. HRMS of compound **5** C_37_H_33_N_3_O_2_ [M+H]^+^ found 552.2757, calcd *m/z* 552.2645.

**Figure** **S119**. HRMS of compound **10** C_33_H_28_N_2_O_5_ [M+Na]^+^ found 555.1943, calcd *m/z* 555.1890.

**Figure S120**. HRMS of compound **9** C_35_H_32_N_2_O_5_ [M+H]^+^ found 561.2318, calcd *m/z* 561.2383.

**Figure S121**. HRMS of compound **8** C_36_H_34_N_2_O_5_ [M+H]^+^ found 575.2193, calcd *m/z* 575.2540.

**IR spectra**

**Figure** **S122**. IR spectrum of compound **3.**

**Figure** **S123**. IR spectrum of compound **2**.

**Figure S124.** IR spectrum of compound **1**.

**Figure S125.** IR spectrum of compound **4.**

**Figure S126.** IR spectrum of compound **7.**

**Figure S127**. IR spectrum of compound **6**.

**Figure S128**. IR spectrum of compound **5**.

**Figure S129**. IR spectrum of compound **10**.

**Figure S130**. IR spectrum of compound **9**.

**Figure S131**. IR spectrum of compound **8**.

**Percentage purity by q^1^HNMR**

**Figure S132**. ^1^H qNMR of compound **1** in DMSO-d_6_ with the dimethylsulfone (DMSO_2_, 99.4% pure) as Internal Calibrant.

Absolute q^1^HNMR with internal calibration

Percentage purity was calculated using the formula

[P%] = n_IC_ **.** Int_t_ **.** MW_t_ **.** m_IC_**/** n_t_ **.** Int_IC_ **.** MW_IC_ **.** m_S_ * P_IC_ = 98.16%

MW = molecular weights

P = purity of internal calibrant

m_IC_ = amount of internal calibrant, m_s_ = amount of sample (compound)

Int = integral, n = no of protons giving rise to a given NMR signal

IC = internal calibrant, t = target analyte or compound

In the given q^1^HNMR experiment,

Ms = 4 mg, m_IC_ = 0.226 mg, P_IC_ = 99.4%

Int_t_ = 104.13^a^, n_t_ = 1, Int_IC_ = 202.03, n_IC_ = 6

MWt = 532 g/mol, MW_IC_ = 94.13 g/mol

^a^ The integral of compound was calculated as the average of integration of all signals.

Calculated percentage purity of compound **1** was 98.16%.

**Figure S133**. Compound **2** docked in the active site of COX-2 showing H-bond (pink lines) with R513 and R120 and π – π interactions (green lines) with W387 and Y385.

**Figure S134**. Compound **3** docked in the active site of COX-2 showing H-bond interactions (pink lines) with W387 and π – π interaction (green lines) with Y355 and Y385.

**Figure S135**. Compound **4** docked in the active site of COX-2 showing π – π interactions (green lines) with Y385, F381 and Y355.

**Figure S136**. Compound **5** docked in the active site of COX-2 showing π – π interaction (green lines) with Y355.

**Figure S137**. Compound **6** docked in the active site of COX-2 showing H-bond (pink line) with Y355 and π – π interaction (green lines) with Y355.

**Figure S138**. Compound 7 docked in the active site of COX-2 showing π – π interactions (lines) with Y385, W387 and R120.

**Figure S139**. Compound **8** docked in the active site of COX-2 showing π – π interactions (green lines) with Y355, W387 and R120.

**Figure S140**. Compound **9** docked in the active site of COX-2 showing H-bond interactions (pink lines) with Y385 and L352 and π – π interaction (green lines) with R120 residue.

**Figure S141**. Compound **10** docked in the active site of COX-2 showing H-bond interactions (pink line) with Y115, Y385 and S530 and π – π interactions (green lines) with Y355, R513 and W387.

**References**

1. Snoeyink, V. L.; Jenkins, D. *Water Chemistry*. John Wiley & Sons, New York, 1980.
2. COX inhibitor screening assay kit (item no. 560131) was purchased from Cayman Chemical Co. and the standard protocols, as supplied with assay kits, were followed for evaluating inhibitory activities.
3. Singh, H.; Kaur, B.; Kaur, H.; Singh, P. A bisubstrate reagent orchestrating adenosine triphosphate and L-tyrosine and making tyrosyl adenylate: partial mimicking of tyrosyl-tRNA synthetase. *Org. Biomol. Chem*. **2018**, *16*, 9446-9453.
